# Impact of age, sex, comorbidities and clinical symptoms on the severity of COVID-19 cases: A meta-analysis with 55 studies and 10014 cases

**DOI:** 10.1016/j.heliyon.2020.e05684

**Published:** 2020-12-15

**Authors:** Md. Abdul Barek, Md. Abdul Aziz, Mohammad Safiqul Islam

**Affiliations:** Department of Pharmacy, Noakhali Science and Technology University, Noakhali, 3814, Bangladesh

**Keywords:** Microbiology, Virology, Viral disease, Travel medicine, Critical care, Health informatics, Covid-19, Pneumonia, Meta-analysis, Severe, Nonsevere, Risk factor, Comorbidity, Clinical manifestation

## Abstract

**Purpose:**

Severe acute respiratory coronavirus 2 (SARS-CoV-2) cases are overgrowing globally and now become a pandemic. A meta-analysis was conducted to evaluate the impact of age, sex, comorbidities, and clinical characteristics on the severity of COVID-19 to help diagnose and evaluate the current outbreak in clinical decision-making.

**Methods:**

PubMed, ScienceDirect, and BMC were searched to collect data about demographic, clinical characteristics, and comorbidities of COVID-19 patients. Meta-analysis was conducted with Review Manager 5.3. Publication bias was assessed using Egger's test and Begg-Mazumdar's rank correlation.

**Results:**

Fifty-five studies were included in this meta-analysis, including 10014 patients with SARS-CoV-2 infection. Male cases and cases with an age of ≥50 years (OR = 2.41, p < 0.00001; RR = 3.36, p = 0.0002, respectively) were severely affected by SARS-CoV-2. Patients having age≥65 years are not associated (p = 0.110) with the severity of COVID-19. Presence of at least one comorbidity or hypertension, diabetes, cerebrovascular disease, cardiovascular diseases, respiratory disease, malignancy, chronic kidney disease and chronic liver diseases individually increased the severity of COVID-19 cases significantly (OR = 3.13, p < 0.00001; OR = 2.35, p < 0.00001; OR = 2.42, p < 0.00001; OR = 3.78, p < 0.00001; OR = 3.33, p < 0.00001; OR = 2.58, p < 0.00001; OR = 2.32, p < 0.00001; OR = 2.27, p = 0.0007; OR = 1.70, p = 0.003, respectively). Clinical manifestation such as fever, cough, fatigue, anorexia, dyspnea, chest tightness, hemoptysis, diarrhea and abdominal pain (OR = 1.68, p = 0.0001; OR = 1.41, p = 0.004; OR = 1.26, p = 0.03; OR = 2.38, p < 0.0001; OR = 4.30, p < 0.00001; OR = 2.11, p = 0.002; OR = 4.93, p < 0.0001; OR = 1.35, p = 0.03; OR = 2.38, p = 0.008, respectively) were significantly associated with the severity of cases. No association of severity was found with myalgia, pharyngalgia, nausea, vomiting, headache, dizziness and sore throat (p > 0.05). No publication bias was found in case of age (≥50 years, age≥65 years), comorbidities and clinical manifestations.

**Conclusions:**

Males patients and elderly or older patients (age ≥50 years) are at higher risk of developing severity, whereas comorbidities and clinical manifestations could significantly affect the prognosis and severity of COVID-19.

## 1 Introduction

The evolving coronavirus disease 2019 (COVID-19), caused by the novel coronavirus (2019-nCoV) or severe acute respiratory syndrome coronavirus 2 (SARS-CoV-2), emerged from Wuhan, Hubei Province, China in late December 2019, declared global pandemic from the World Health Organization (WHO) on 11^th^ March 2020 due to its worldwide potential and deathly outcomes [1, 2]. This deadly infection is mainly transmitting through large respiratory droplets of affected people during coughing or sneezing, though the virus's presence has also been traced from stool and urine of infected individuals [3]. The most common COVID-19 patients' symptoms are fever, dry cough, fatigue, nasal congestion, myalgia, sore throat, and diarrhea, whereas the comorbidities are diabetes, hypertension, respiratory disease, cardiovascular disease, malignancy and others [4, 5, 6].

Most coronaviruses can infect different animals, including humans. At present, there are seven classes of coronaviruses that have been isolated from humans, including α-coronaviruses (229E and NL63), β-coronaviruses (OC43), Middle East Respiratory Syndrome Coronavirus (MERS-CoV), HKU1, and Severe Acute Respiratory Syndrome Coronavirus (SARS-CoV) [7, 8, 9]. SARS-CoV2 was isolated from the lower respiratory tract of patients suffering from pneumonia in Wuhan, and it was named as 2019-nCoV [10]. The International Committee on the Taxonomy of Viruses (ICTV), on the other hand, officially renamed it SARS-CoV-2 [10,11]. It is very similar to the genome sequences of previously identified coronaviruses, most importantly, to the SARS-CoV [12, 13]. So, this novel coronavirus has been classified as a β-coronavirus which can be transmitted into humans.

Currently, more than 213 countries and territories have confirmed the infection of this contagious virus. The infection rate is rising globally, as confirmed by the WHO, according to an exponential trend. As of May 29, 2020, approximately 5,962,944 confirmed cases of COVID-19 were identified with a total death of 363,905 (6.10%) patients worldwide [14]. Accordingly, countries across the world have undertaken rapid regulatory and migratory activities in response to the COVID-19 attack to control major patient outbreaks and to level the demand for increased hospital beds, testing facilities, oxygen and mechanical ventilation supports while protecting the most vulnerable one from infection, including elderly or older people with comorbidities to reduce their mortalities [15]. However, severe patients need more intensive care that is somehow lacking in most of the countries.

The current knowledge about the characteristics of novel coronavirus is still limited, and this is transmitting rapidly. To understand both the situation and the seriousness of the disease, health workers and researchers have made remarkable efforts concerning new coronavirus infected patients. The healthcare providers have proposed numerous recommendations for overcoming both diagnostic and therapeutic challenges as there are no approved drugs for protecting this assailable population from contagious viral exposure. Moreover, as there are no established vaccines, researchers are trying to develop vaccines to tackle this pandemic [16, 17].

The global outbreak of highly contagious coronavirus has led the nations' medical, psychological, and socio-economic conditions to a challenging situation that they never thought before. COVID-19 portrays probably one of the greatest threats in this century that the countries have to tackle. Therefore, scientists are trying to understand the pathogenesis, clinical implications, and develop novel preventive strategies. To date, researches on this pandemic have produced many scientific results on the clinicopathological findings that are not consistent. We analyzed relevant data from published articles to conduct the present meta-analysis to identify epidemiological attributes, clinical features, the frequency of comorbidities, severity of the infection, the correlation of age, sex, comorbidities, clinical manifestations with the severity of COVID-19 cases for more accurate and precise outcomes. We hope this study will help the existing clinical practices on the prevention, treatment, and management of the pandemic.

## 2 Methods

### 2.1 Literature search strategy

The present meta-analysis was carried out following the guidelines of Preferred Reporting Items for Systematic Reviews and Meta-Analyses (PRISMA). The relevant studies written in any language were systematically searched on PubMed, ScienceDirect and BMC Journal database from January 1, 2020, to May 23, 2020. EndNote X 7.0 software was employed to exclude duplicate studies. The following keywords are used in search alone or in combination: ‘clinical characteristics of COVID-19’, ‘severity of COVID-19’, ‘clinical outcome’, ‘death or clinical features’, ‘comorbidities of COVID-19’, ‘signs and symptoms of SARS-CoV-2’ There was no country limitation to identify the studies and search was limited to humans, but only online literature was included. We have reviewed reference lists of included articles to identify missing studies.

### 2.2 Inclusion and exclusion criteria

The criteria need to be satisfied for inclusion studies are below:

1) Only study samples with confirmed COVID-19 infection; 2) Studies with age, sex, clinical signs & symptoms, comorbidities, disease severity, deaths, and survival as primary outcomes; 3) Cohort studies and case-control studies; 4) No language and geographical restriction; 5) Study with human samples; 6) Studies with sufficient data to calculate OR and 95% CI.

The criteria for exclusion are as follows:

1) Expert opinions, reviews, letters, and commentaries; 2) Studies with children and pregnant women case; 3) Overlapping or duplicate publications; 4) Irrelevant information for data extraction; 5) Animal studies.

### 2.3 Data extraction

Two investigators (MAB and MAA) independently extracted data with the inclusion criteria. They separately performed the literature search, evaluation, and data extraction to an excel database. Regarding the disagreements of the studies that emerged during the process were resolved by another investigator (MSI). Rayyan QCRI, a systematic review web app, was used to select the studies [18]. Data extraction included the author's name, country, age, sex, number of participants, comorbidities, clinical symptoms, and severe and nonsevere cases.

### 2.4 Methodological quality assessment

‘Newcastle-Ottawa Scale (NOS)’ was utilized for observational cohort studies to determine the methodological quality of the included studies, as described elsewhere [19]. Any disagreement between investigators was settled through discussion.

### 2.5 Statistical analysis, heterogeneity, and publication bias

The data analyses were performed by Microsoft Excel and Review Manager 5.3 (RevMan 5.3, the Cochrane Collaboration, Oxford, United Kingdom) software. Review Manager 5.3 was utilized to evaluate the heterogeneity (χ2 and I^2^) between studies, and heterogeneity in the forest plot was evaluated, applying both the Cochran's chi-square Q-test and I^2^ statistic. p < 0.1 or I^2^ > 50% indicated the presence of statistically significant heterogeneity. Accordingly, I^2^ values of 25%, 50%, and 75% represented low, moderate, and high heterogeneity. To determine any significant variations in risk across the studies for each parameter, we conducted a sensitivity analysis by omitting studies one-by-one in a certain order. The random-effect model was selected throughout the analysis. Publication biases were evaluated by the funnel plot along with Egger's regression test and Begg-Mazumdar's rank correlation. The level of significance selected for publication bias was p < 0.05, and the values higher than this were predicted as no publication bias.

## 3 Results

### 3.1 Study selection and quality assessment

**Figure 1:**
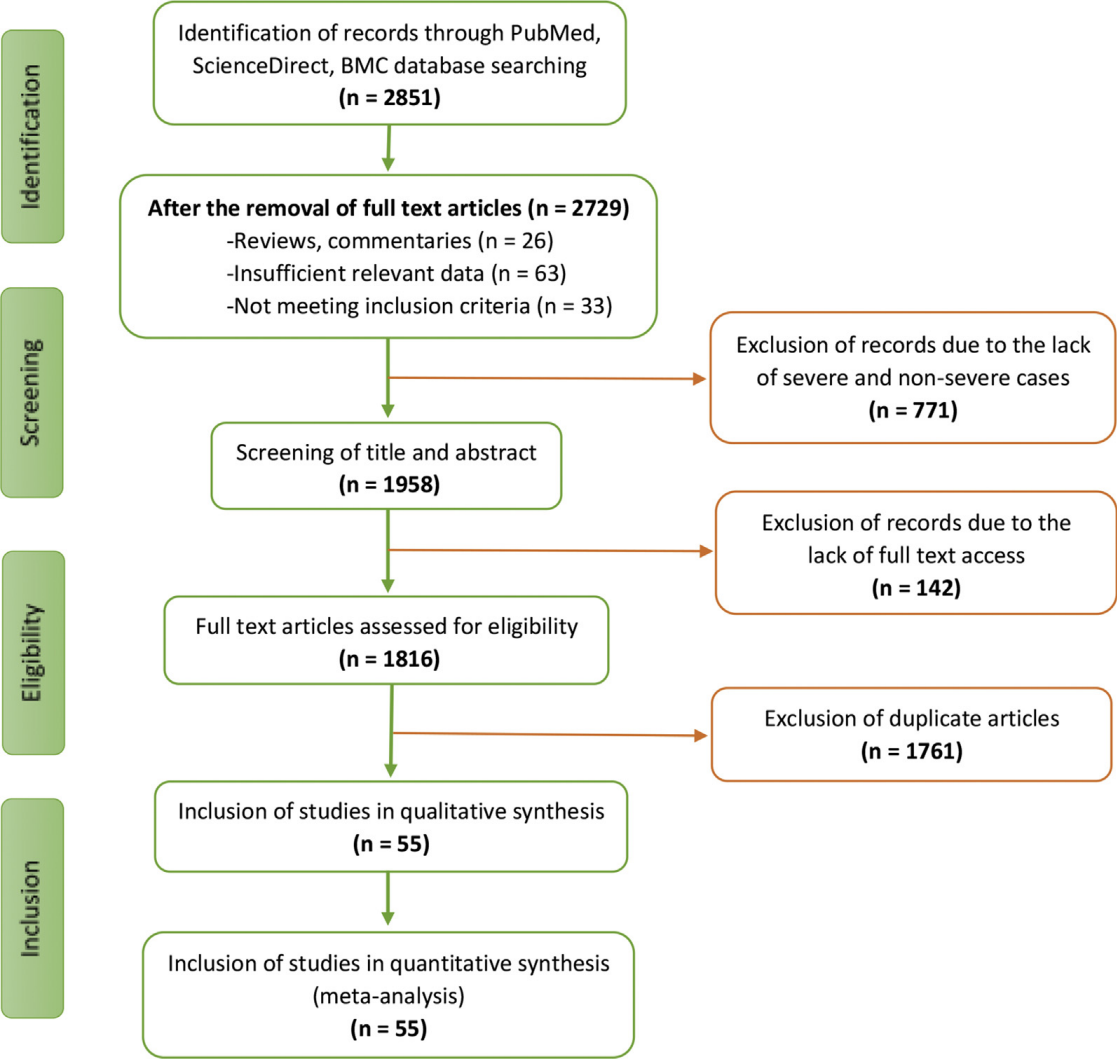
Flow chart illustrating the literature search and study selection.

**Figure 2:**
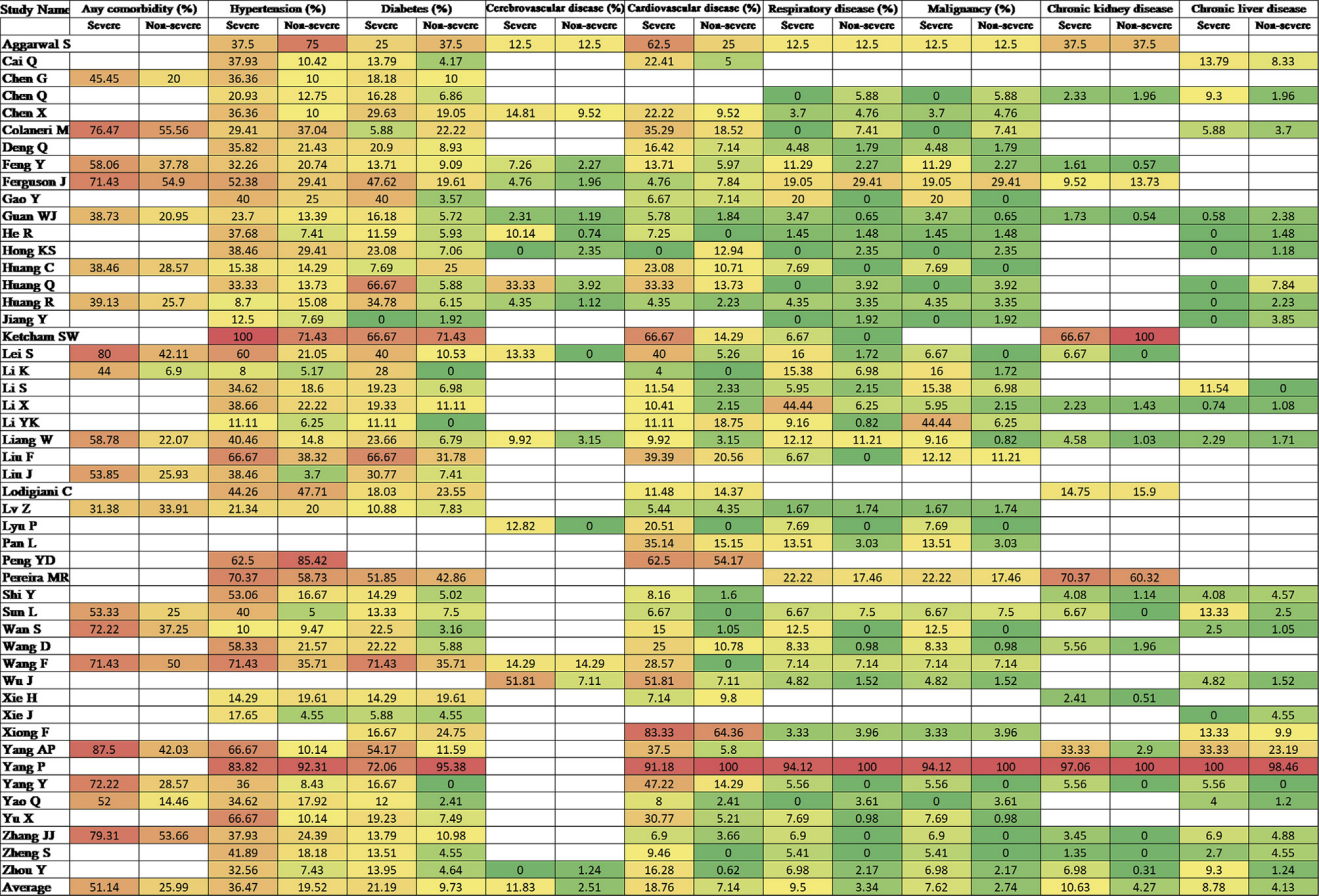
Comorbidities of COVID-19 cases of the included studies

**Figure 3:**
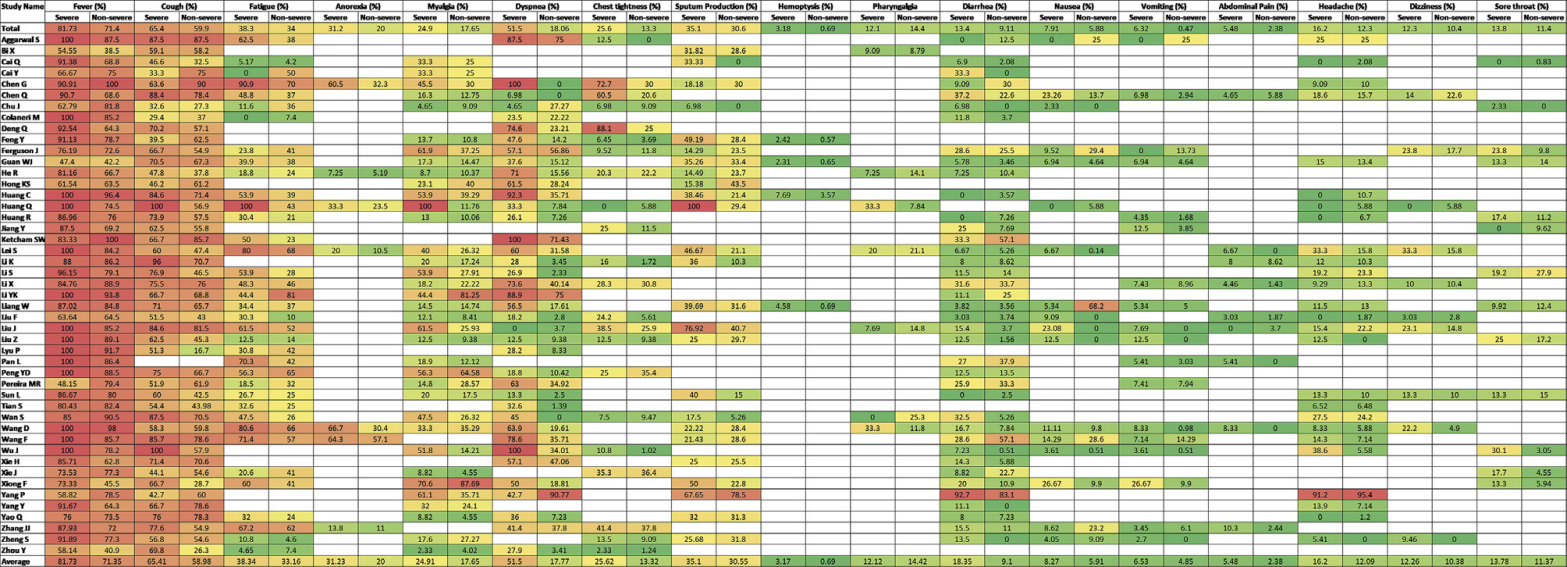
Clinical symptoms of COVID-19 cases of the included studies

Initially, 2851 articles were identified from three databases (PubMed, ScienceDirect, BMC) during the initial retrieval. A total of 1761 records were excluded because of duplication. Then, 771 articles were removed after reading the title and abstract, and 142 were excluded from the remaining 319 articles for various reasons. In the end, 55 full-text studies involving 10014 COVID-19 patients were included in this meta-analysis based on the detailed assessment and inclusion criteria (Figure 1) [20, 21, 22, 23, 24, 25, 26, 27, 28, 29, 30], [31, 32, 33, 34, 35, 36, 37, 38, 39, 40], [41, 42, 43, 44, 45, 46, 47, 48, 49, 50], [51, 52, 53, 54, 55, 56, 57, 58, 59, 60], [61, 62, 63, 64, 65, 66, 67, 68, 69, 70, 71, 72, 73, 74]. It was also found that most of these studies (n = 49) were based in China, although three studies were identified from the USA, two studies were from Italy, and one study from South Korea was included. The quality of most included studies was of high quality (score ranges between 6-8) assessed by the Newcastle Ottawa scale. Only two studies being of moderate quality (score 5), as shown in Supplementary Table S1. The baseline characteristics of all studies are presented in Table 1, and other results are presented in Figures 2, 3 and Tables 2, 3.

**Table 1:** Baseline characteristics of included studies.

Study ID	Year	Country	Study Design	Sample Size	Male (%)	Median Age/Mean ± SD	Follow-up/Observation period/Data collection period, days
Aggarwal S	2020	USA	retrospective study	16	12 (75%)	67/65.5	36
Bi X	2020	China	retrospective study	113	64 (56.6%)	46	25
Cai Q	2020	China	retrospective study	298	145 (48.66%)	47.5	55
Cai Y	2020	China	retrospective study	7	5 (71.43%)	60.29	-----
Chen G	2020	China	retrospective study	21	17 (81%)	56	-----
Chen X	2020	China	retrospective study	48	37 (77.1%)	64.6 ± 18.1	19
Chen Q	2020	China	single center retrospective observational study	145	79 (54.5%)	47.5	71
Chu J	2020	China	single center retrospective observational study	54	36 (66.7%)	39	35
Colaneri M	2020	Italy	retrospective study	44	28 (63.64%)	67.5	13
Deng Q	2020	China	retrospective study	112	57 (50.9%)	65.0	45
Feng Y	2020	China	multi-center retrospective study	476	271 (56.9%)	53.0	46
Ferguson J	2020	USA	retrospective study	72	38 (52.8%)	60.4	29
Gao Y	2020	China	retrospective study	43	26 (60.47%)	44.08	10
Guan WJ	2020	China	retrospective study	1099	637 (57.96%)	47.0	49
He R	2020	China	retrospective study	204	79 (38.73%)	49.0	34
Hong KS	2020	South Korea	Descriptive Study	98	38 (38.8%)	55.4 ± 17.1	90
Huang C	2020	China	prospective study	41	30 (73%)	49.0	17
Huang Q	2020	China	multi-center retrospective study	54	28 (51.9%)	41.0	24
Huang R	2020	China	multi-center retrospective study	202	116 (57.4%)	44.0	19
Jiang Y	2020	China	single center retrospective study	60	35 (58.33%)	41	17
Ketcham SW	2020	China	retrospective study	13	13 (100%)	61.0	46
Lei S	2020	China	retrospective study	34	14 (41.2%)	55.0	36
Li K	2020	China	retrospective study	83	44 (53%)	45.5	60
Li S	2020	China	retrospective study	69	40 (57.97%)	48.5	60
Li X	2020	China	retrospective study	548	279 (50.9%)	60.0	37
Li YK	2020	China	retrospective study	25	13 (52%)	61	51
Liang W	2020	China	retrospective study	1590	904 (57.4%)	48.9	26
Liu F	2020	China	retrospective study	140	49 (35.0%)	65.5	54
Liu J	2020	China	retrospective single-center study	40	15 (37.5%)	48.7 ± 13.9	19
Liu Z	2020	China	retrospective study	72	39 (54.2%)	46.2 ± 15.9	28
Lodigiani C	2020	Italy	retrospective study	388	264 (68%)	66.0	57
Lv Z	2020	China	retrospective cohort study	354	175 (49.44%)	62.0	24
Lyu P	2020	China	retrospective study	51	29 (56.86%)	54 ± 17	40
Pan L	2020	China	descriptive, cross-sectional, multicenter study	103	37 (35.92%)	48.2	60
Peng YD	2020	China	retrospective study	112	53 (47.32%)	62.0	26
Pereira MR	2020	USA	retrospective study	90	53 (59%)	57.0	20
Shi Y	2020	China	retrospective study	487	259 (53.2%)	46.0	15
Sun L	2020	China	retrospective study	55	31 (56.4%)	44.0	26
Tian S	2020	China	retrospective study	262	127 (48.5%)	47.5	21
Wan S	2020	China	retrospective study	135	72 (53.3%)	47	16
Wang D	2020	China	retrospective single-center study	138	75 (54.3%)	56	34
Wang R	2020	China	single-center, retrospective, descriptive study	125	54 (43.2%)	38.76 ± 13.80	29
Wang F	2020	China	retrospective study	28	21 (75.0%)	68.6 ± 9.0	24
Wu J	2020	China	retrospective study	280	151 (53.93%)	43.12 ± 19.02	31
Xie H	2020	China	retrospective study	79	44 (55.7%)	60.0	21
Xie J	2020	China	retrospective study	56	24 (42.86%)	56.5	10
Xiong F	2020	China	retrospective study	131	75 (57.3%)	63.3	70
Yang AP	2020	China	retrospective study	93	56 (60%)	46.4 ± 17.6	28
Yang P	2020	China	retrospective study	133	72 (54.14%)	50.60	90
Yang Y	2020	China	retrospective study	50	29 (58%)	62.0	39
Yao Q	2020	China	retrospective study	108	43 (39.8%)	52.0	12
Yu X	2020	China	descriptive study	333	172 (51.7%)	56.0	26
Zhang JJ	2020	China	descriptive study	140	71 (50.7%)	57.0	18
Zheng S	2020	China	retrospective study	96	58 (60%)	55.0	61
Zhou Y	2020	China	multi-center retrospective study	366	207 (56.6%)	43.0	37

**Table 2:** Results of the meta-analysis of the sex, age, comorbidity and clinical manifestation.

Overall Parameter	Individual Parameter	∗OR or RR	95% CI	p value	I^2^
Sex	Sex	2.41	1.93–3.02	<0.00001	67%
Age	Age≥50 vs. age<50	3.36	1.79–6.30	0.0002	89%
	Age≥65 vs. age<65	0.79	0.59–1.06	0.11	88%
Comorbidity	Any comorbidity	3.13	2.26–4.32	<0.00001	64%
	Hypertension	2.35	1.83–3.02	<0.00001	66%
	Diabetes	2.42	1.84–3.19	<0.00001	58%
	Cerebrovascular disease	3.78	2.22–6.43	<0.00001	35%
	Cardiovascular disease	3.33	2.47–4.47	<0.00001	47%
	Respiratory disease	2.58	1.76–3.77	<0.00001	33%
	Malignancy	2.32	1.63–3.32	<0.00001	9%
	Chronic kidney disease	2.27	1.41–3.65	0.0007	32%
	Chronic liver disease	1.70	1.19–2.42	0.003	0%
	Overall	2.59	2.31–2.89	<0.00001	49%
Symptoms	Fever	1.68	1.29–2.19	0.0001	54%
	Cough	1.41	1.11–1.77	0.004	63%
	Fatigue	1.26	1.03–1.55	0.03	36%
	Anorexia	2.38	1.60–3.54	<0.0001	0%
	Myalgia	1.30	0.98–1.71	0.07	58%
	Dyspnea	4.30	2.98–6.22	<0.00001	79%
	Chest tightness	2.11	1.30–3.42	0.002	72%
	Sputum production	1.35	1.00–1.82	0.05	55%
	Hemoptysis	4.93	2.43–10.02	<0.0001	0%
	Pharyngalgia	0.91	0.30–2.74	0.87	69%
	Diarrhea	1.35	1.03–1.78	0.03	30%
	Nausea	1.26	0.48–3.31	0.64	86%
	Vomiting	1.48	0.97–2.25	0.07	24%
	Abdominal pain	2.38	1.25–4.52	0.008	0%
	Headache	1.19	0.83–1.72	0.34	48%
	Dizziness	1.40	0.87–2.28	0.17	22%
	Sore throat	1.60	0.88–2.91	0.12	66%
	Overall	1.62	1.46–1.79	<0.00001	68%

∗ Risk Ratio (RR) was used only for age.

**Table 3:** Publication bias was examined by Egger's linear regression test and Begg and Mazumdar's rank correlation test.

Parameters	p-value (Egger's test_)	p-value (Begg-Mazumdar's test)
Sex	0.063	0.126
Age (≥50 vs. <50 years)	0.116	0.835
Age (≥65 vs. <65 years)	0.926	0.891
Any comorbidity	0.300	0.600
Hypertension	0.953	0.992
Diabetes	0.872	0.754
Cerebrovascular disease	0.207	0.656
Cardiovascular disease	0.731	0.683
Respiratory diseases	0.085	0.654
Malignancy	0.334	0.218
Chronic kidney disease	0.542	1.000
Chronic liver disease	0.751	0.779
Fever	0.035	0.819
Cough	0.125	0.977
Fatigue	0.638	0.657
Anorexia	0.208	0.805
Myalgia	0.793	0.563
Dyspnoea	0.803	0.780
Chest tightness	0.122	0.324
Sputum production	0.813	0.513
Haemoptysis	0.424	0.608
Pharyngalgia	0.675	0.543
Diarrhea	0.305	0.762
Nausea	0.304	0.742
Vomiting	0.287	0.472
Abdominal pain	0.684	0.677
Headache	0.914	0.235
Dizziness	0.164	0.531
Sore throat	0.474	0.625

### 3.2 Effect of sex on disease severity

**Figure 4:**
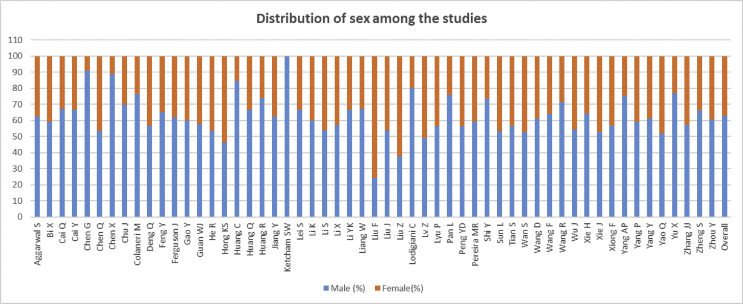
Distribution of sex of included studies to analyze the effect of sex on for the severity of COVID-19.

**Figure 5:**
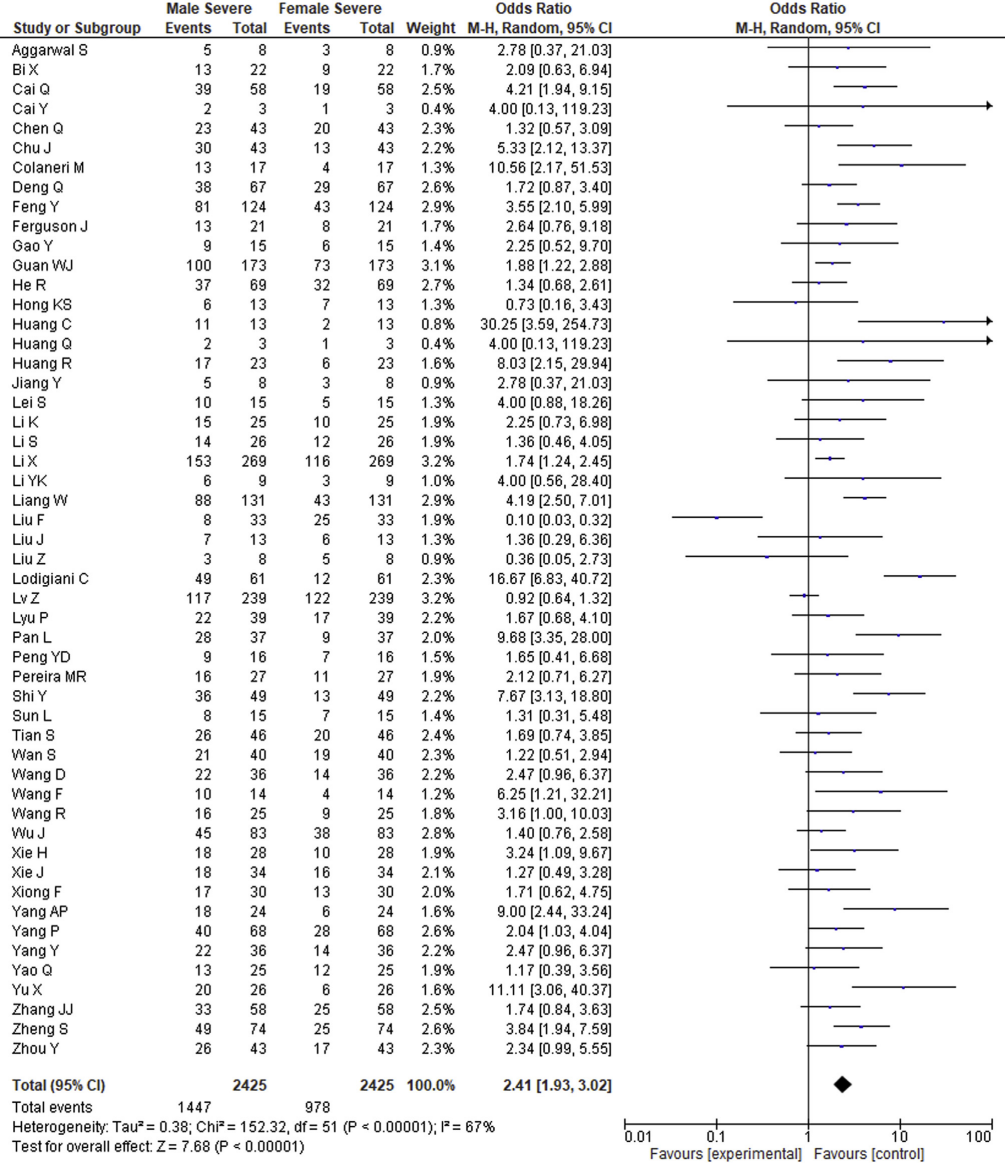
Meta-analysis for the effect of sex on the severity of COVID-19 cases. Forest plots depict the comparison of the incidences of male and female in severe and nonsevere patients.

Among the 10014 patients, 2469 were severe or critical cases, 7545 were nonsevere patients. Males (62.83%) were found more than females (37.17%) in severe cases (Figure 4), whereas the males were 53.04%, and 46.6 % were female in nonsevere cases. Significant heterogeneity was found when compared the severity among the male and female COVID-19 patients (I^2^ = 67%, p < 0.00001). The random-effect model was used in the meta-analysis, and the results showed that the proportion of severe patients in the males was significantly higher than the females and male patients showed 2.41 times more risk of the development severe COVID-19 than female patients (male Vs. female 59.67% vs. 40.33%, OR = 2.41, 95%CI = 1.93–3.02, p < 0.00001) (Table 2, Figure 5.

### 3.3 Effect of age on the severity

**Figure 6:**
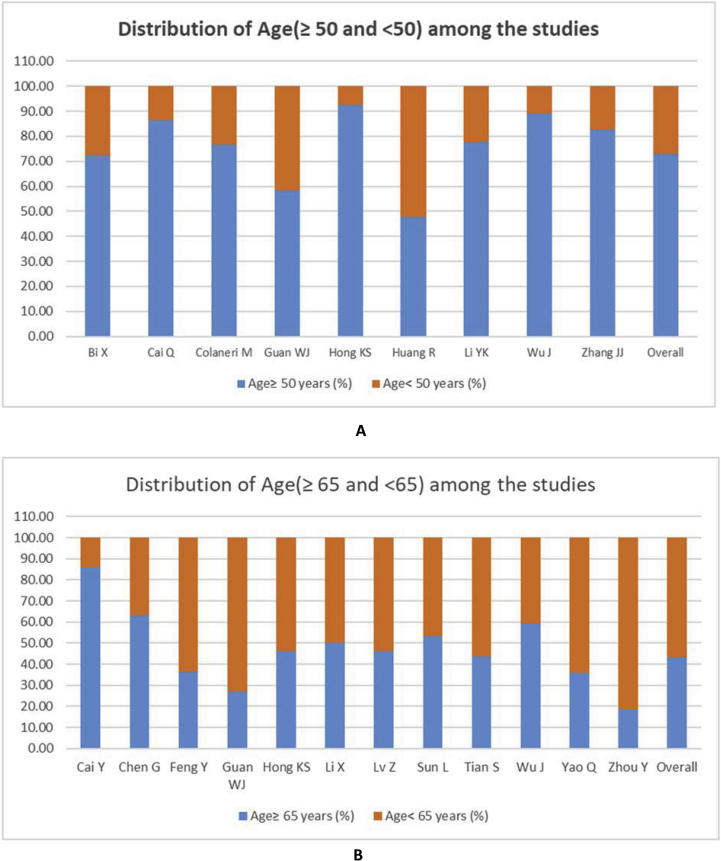
Distribution of age A. ≥ 50 Vs. <50 years and B. ≥ 65 and <65 years among the included studies to analyze the effect of age on for the severity of COVID-19.

**Figure 7:**
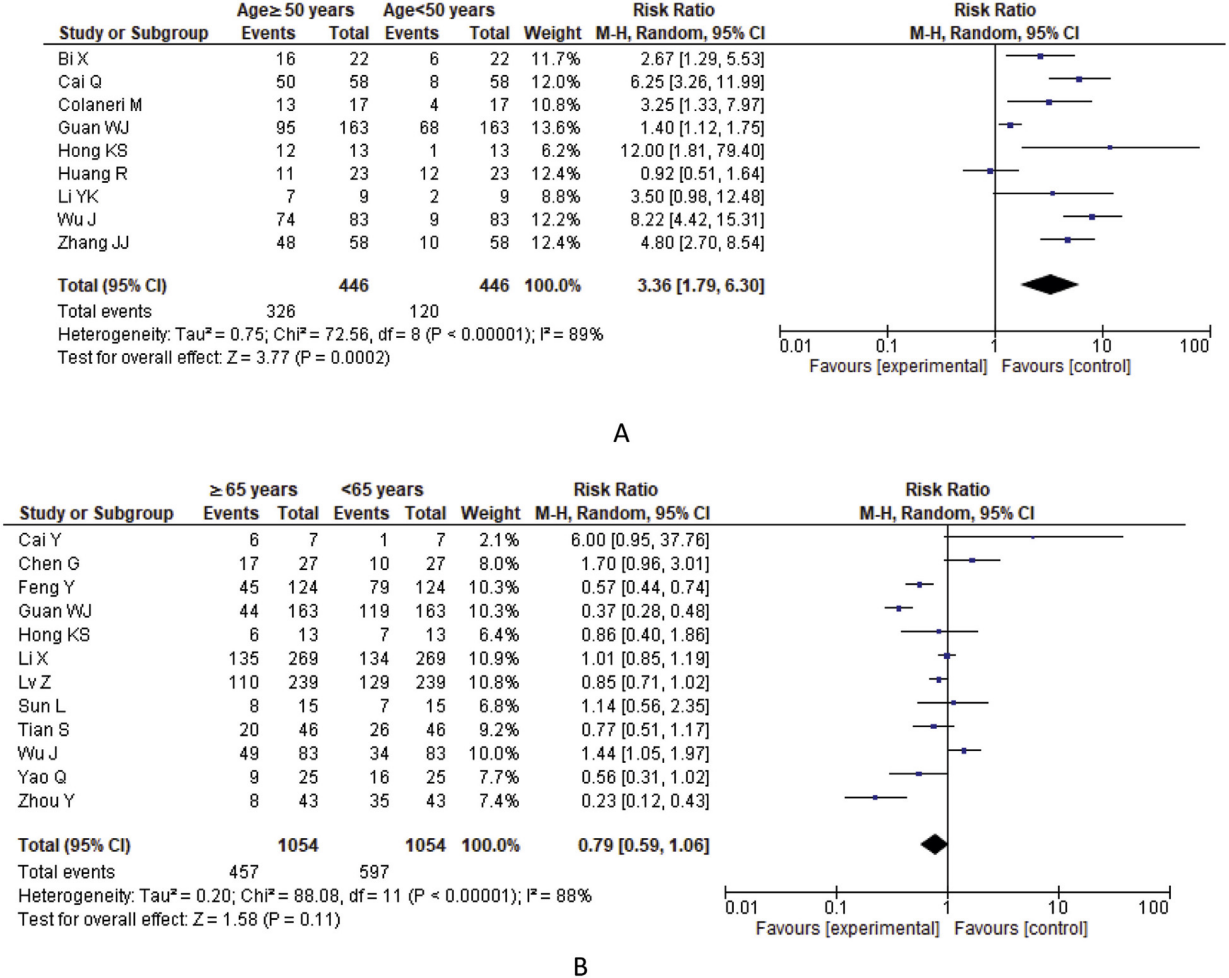
Meta-analysis for the effect of age on the severity of COVID-19 cases. Forest plots depict the comparison of the incidences of A) age ≥50 vs. age<50 years B) age ≥65 vs. age<65 years in severe patients.

Studies that provided only median or mean age were excluded from the analysis of the association of severity with age. Among the 55 studies, the severe patients of 8 studies were categorized as age ≥50 years (73.09%) and <50 years (26.91%), whereas 12 studies were categorized as age ≥65 years (43.36%) and <65 years (56.64%) (Figure 6). A higher significant heterogeneity also found in both age≥50 vs. age<50 years (I^2^ = 89%, p < 0.00001) and age≥65 vs. age<65 (I^2^ = 88%, p < 0.00001) groups. COVID-19 patients with age ≥50 years showed statistically significant 3.36 times more risk of severity in comparison with age below 50 years (age≥50 years Vs. age<50 years, RR = 3.36; 95% CI = 1.79–6.30, p = 0.0002) whereas patients with age ≥65 years showed 0.79 times risk compared to severe patients age below 65 years (age≥65 years Vs. age<65 years, RR = 0.79; 95% CI = 0.59–1.06, p = 0.110) (Table 2, Figure 7).

### 3.4 Effect of comorbidity on the disease severity

**Figure 8:**
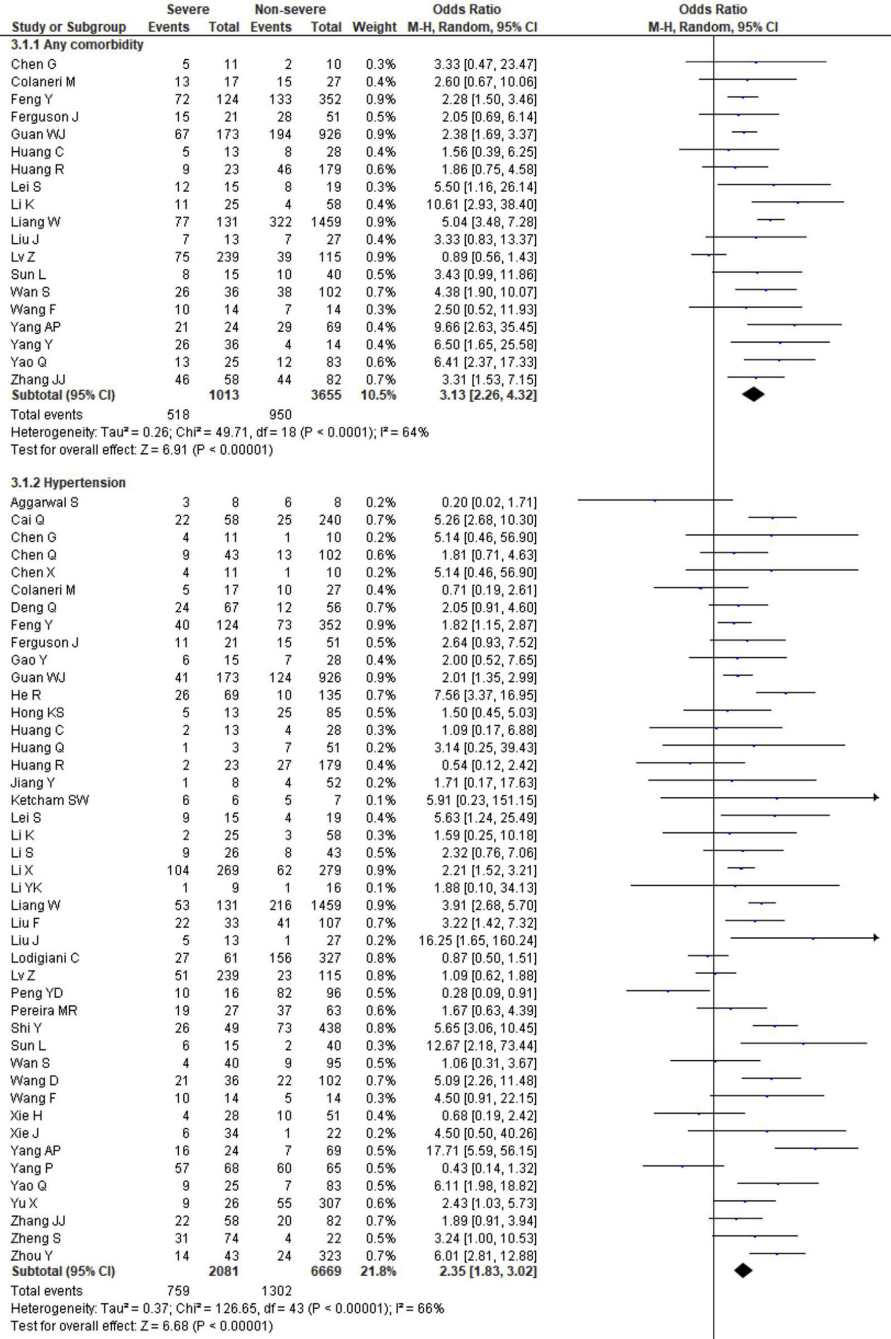

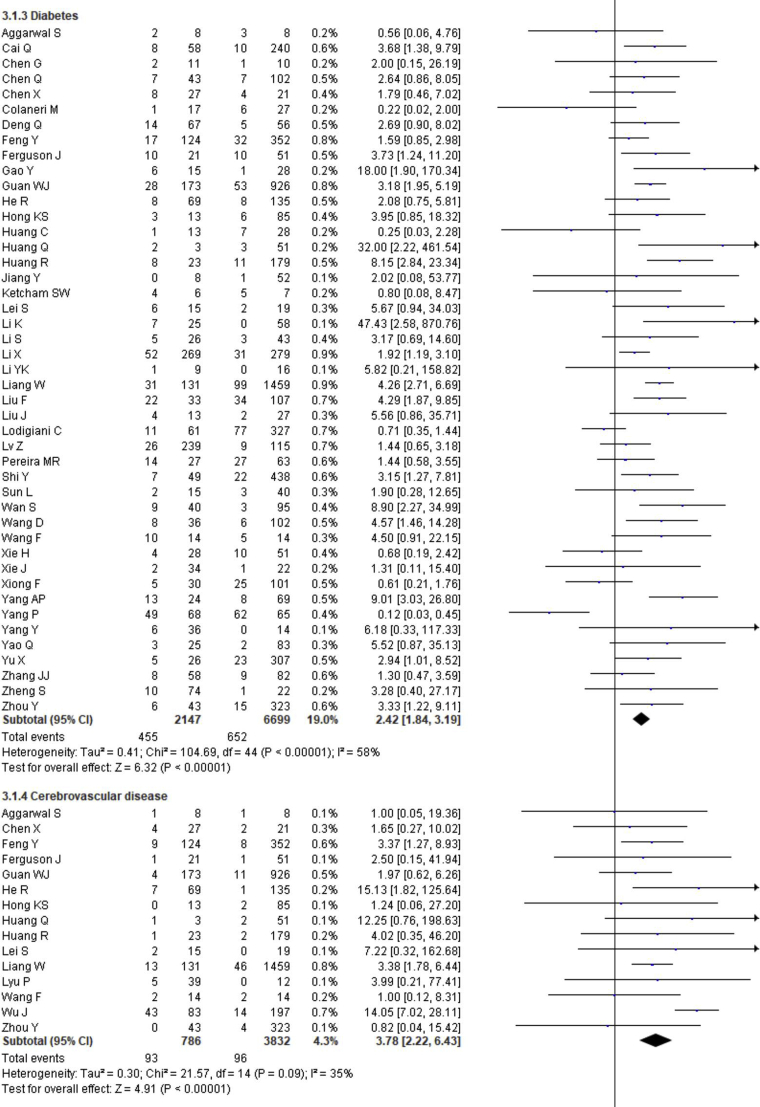

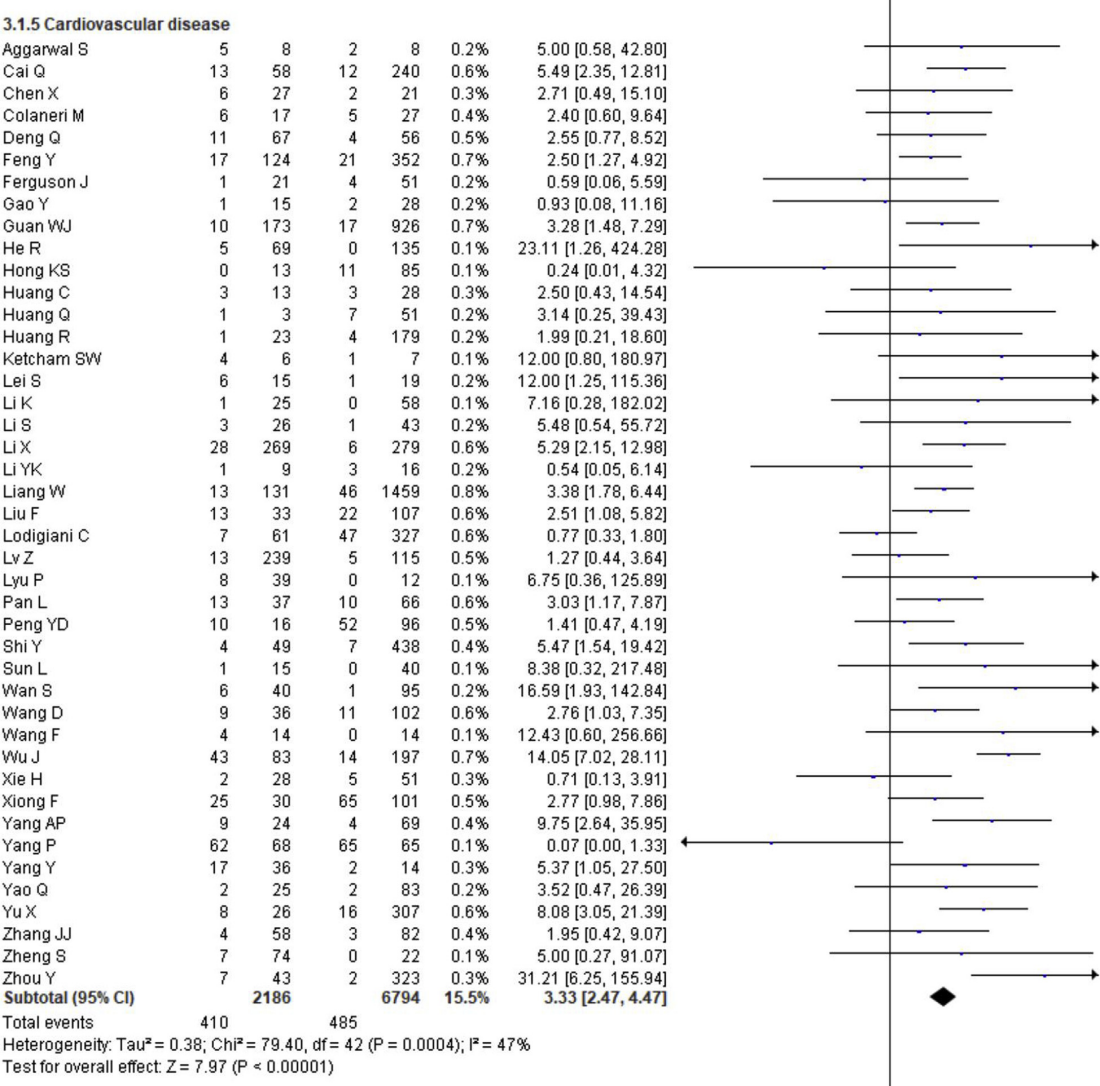

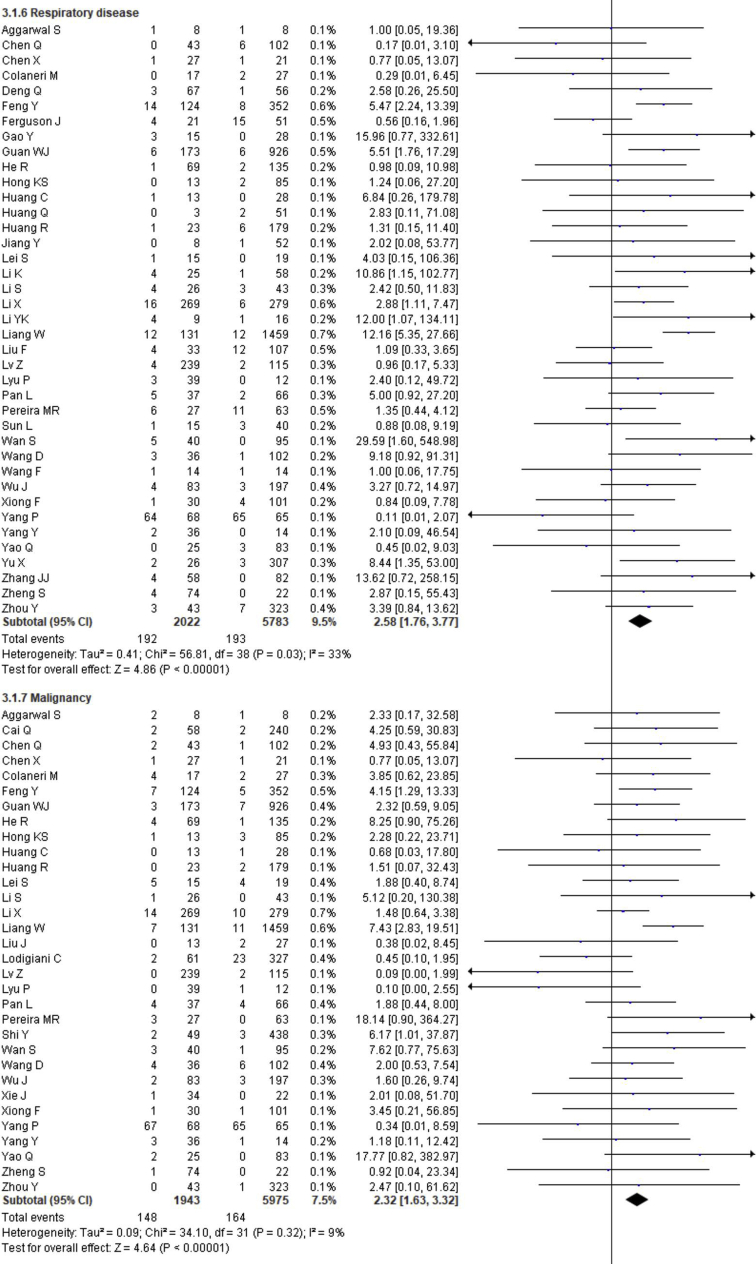

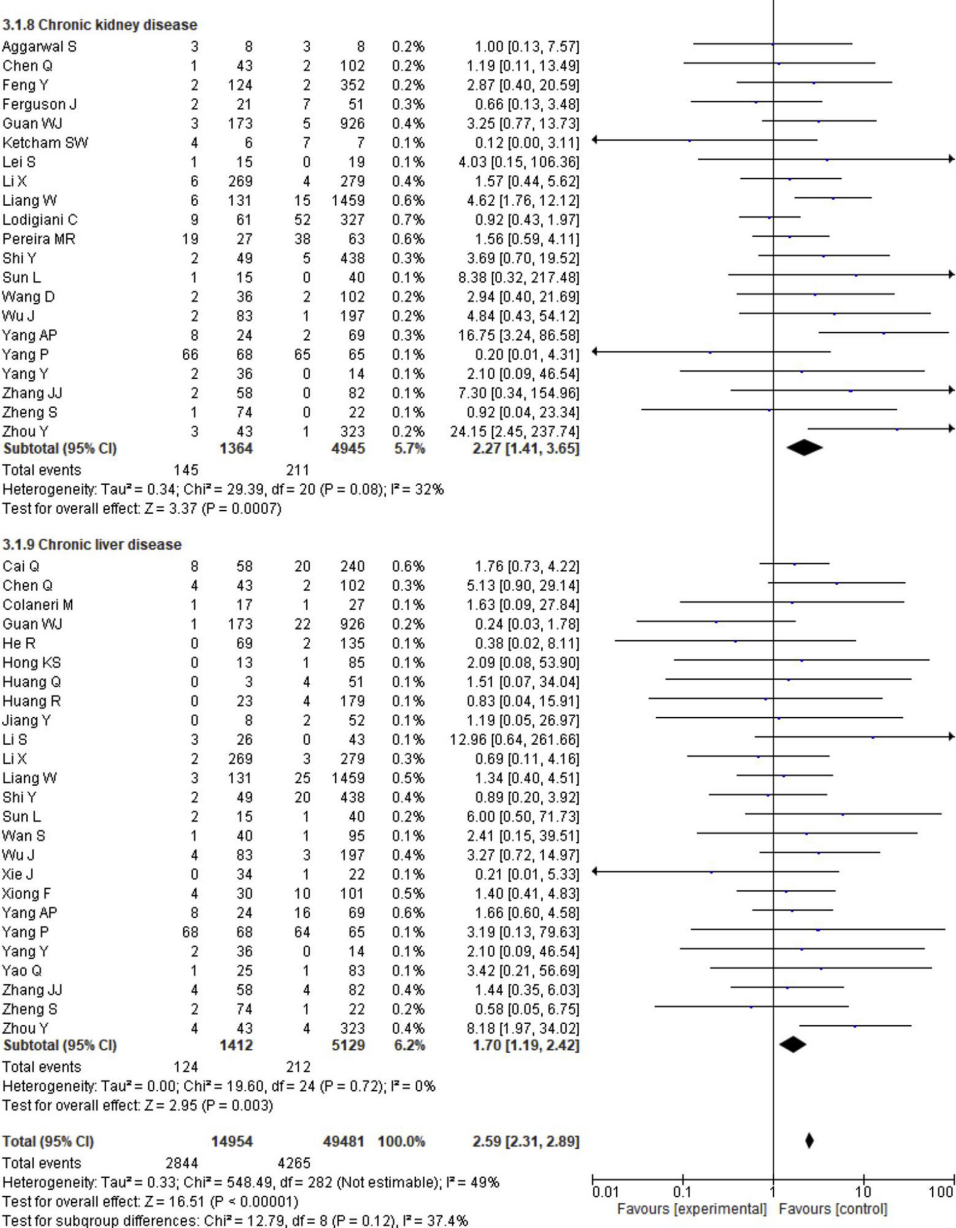
Meta-analysis for the effect of comorbidities on the severity of COVID-19 cases. Random effect model for any comorbidity, hypertension, diabetes, cerebrovascular disease, cardiovascular disease, respiratory disease, malignancy, chronic kidney disease and chronic liver disease.

The prevalence of comorbidities including the presence of at least one comorbidity, hypertension, diabetes, cerebrovascular disease, cardiovascular diseases, respiratory disease, malignancy, chronic kidney disease and chronic liver disease in severe and non-severe COVID-19 patients of the included studies is shown in Figures 2, 8 and Table 2. Among the different comorbidities, 51.14% of severe COVID-19 cases had at least one comorbidity and patients having at least one comorbidity had 3.13 times more risk of severe illness than nonsevere patients (severe vs. nonsevere: 51.14 vs. 25.99: OR = 3.13, 95% CI = 2.26–4.32, p < 0.00001, I^2^ = 64%). A total of 36.47 % of severe patients had hypertension as comorbidity, and the severity of illness was found 2.35 times higher in COVID-19 cases having preexisting hypertension (severe vs. nonsevere: 36.47% vs. 19.52%, OR = 2.35, 95% CI = 1.83–3.02, p < 0.00001, I^2^ = 66%). The proportion of severe illness in cerebrovascular disease (severe vs. nonsevere: 11.83% vs. 2.51%) and cardiovascular disease (severe vs. nonsevere: 18.76% vs. 7.14%) was also higher than the non-severe patients and the disease severity are strongly associated with preexisting cerebrovascular and cardiovascular diseases (cerebrovascular disease: OR = 3.78, 95% CI = 2.22–6.43, p < 0.00001, I^2^ = 35%; cardiovascular disease: OR = 3.33, 95% CI = 2.47–4.47, p < 0.00001, I^2^ = 47%). Preexisting diabetes (severe vs. nonsevere: 21.19% vs. 9.73%), respiratory disease (severe vs. nonsevere: 9.50% vs. 3.34%) and malignancy (severe vs. nonsevere: 7.62% vs. 2.74%) also significantly increased the severity of COVID-19 cases (diabetes: OR = 2.42, 95% CI = 1.84–3.19, p < 0.00001, I^2^ = 58%; respiratory disease: OR = 2.58, 95% CI = 1.76–3.77, p < 0.00001, I^2^ = 33%; malignancy: OR = 2.32, 95% CI = 1.63–3.32, p < 0.00001, I^2^ = 9%). Chronic liver disease (CLD, severe vs. nonsevere: 8.78% vs. 4.13%) and chronic kidney disease (CKD, severe vs. nonsevere: 10.63% vs. 4.27%) were also found as risk factors for increasing the severity of COVID-19 (CLD: OR = 1.70, 95% CI = 1.19–2.42, p = 0.003, I^2^ = 0; CKD: OR = 2.27, 95% CI = 1.41–3.65, p = 0.0007, I^2^ = 32%).

### 3.5 Effect of clinical symptoms on the disease severity

**Figure 9:**
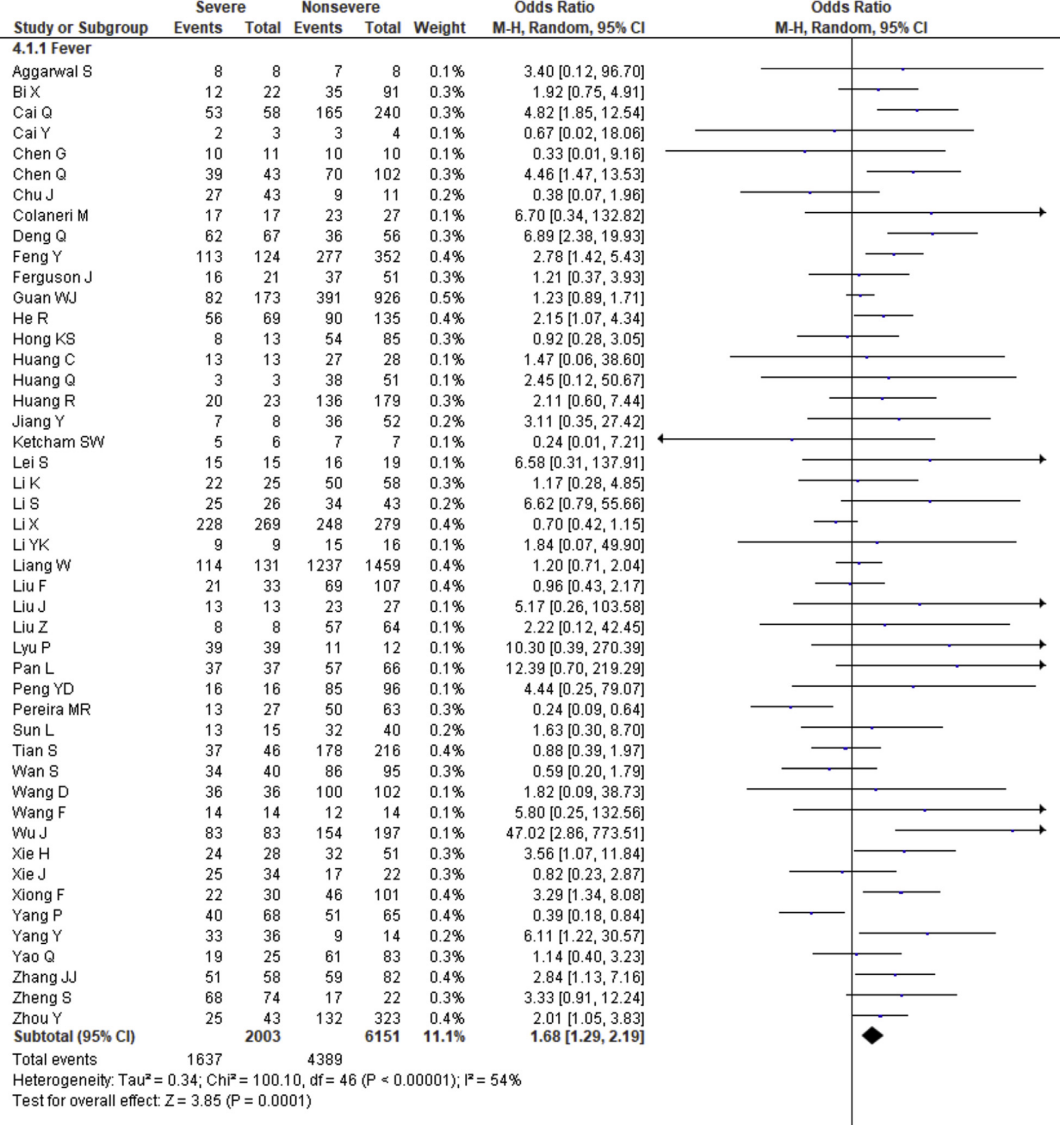

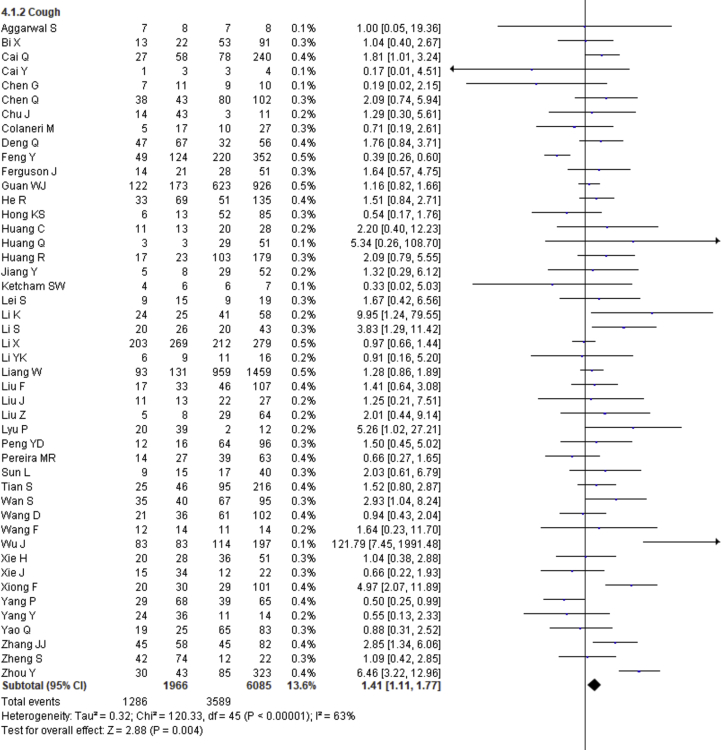

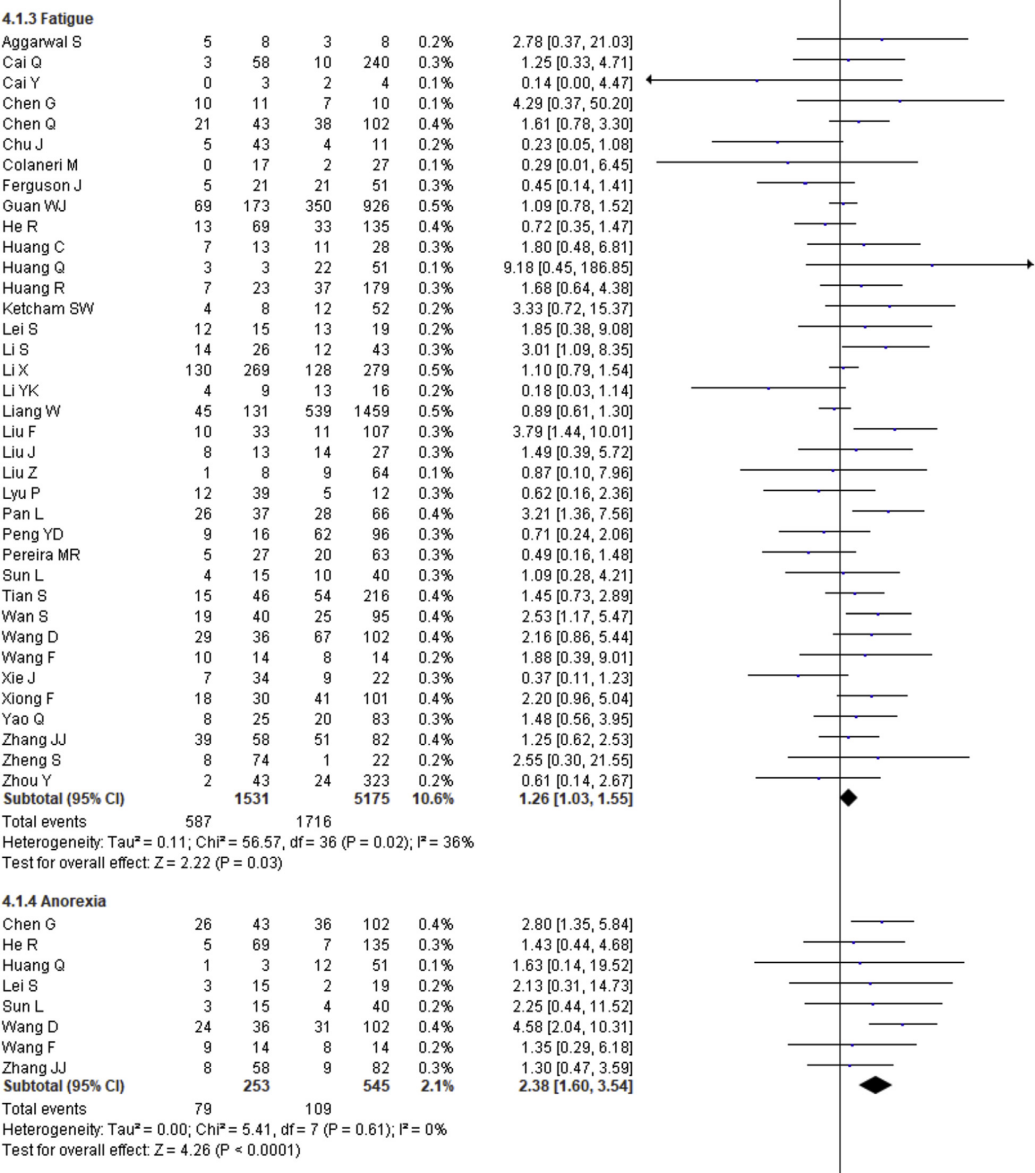

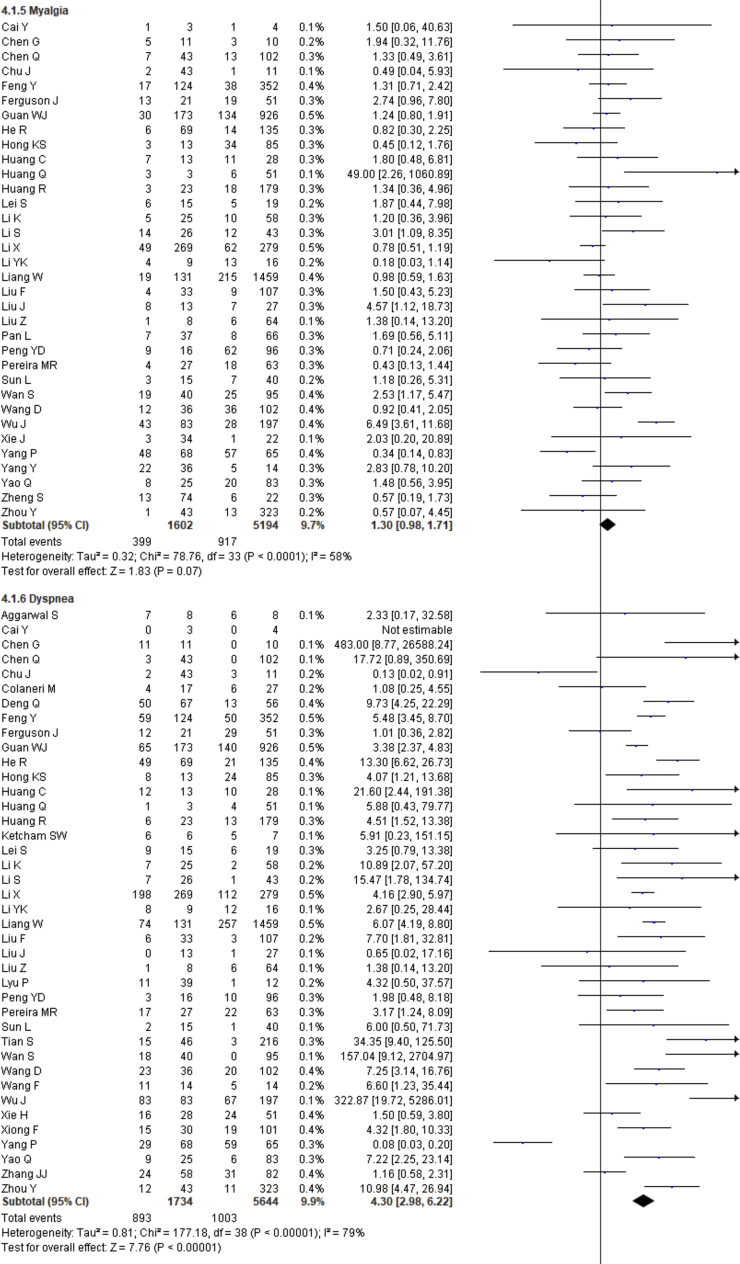

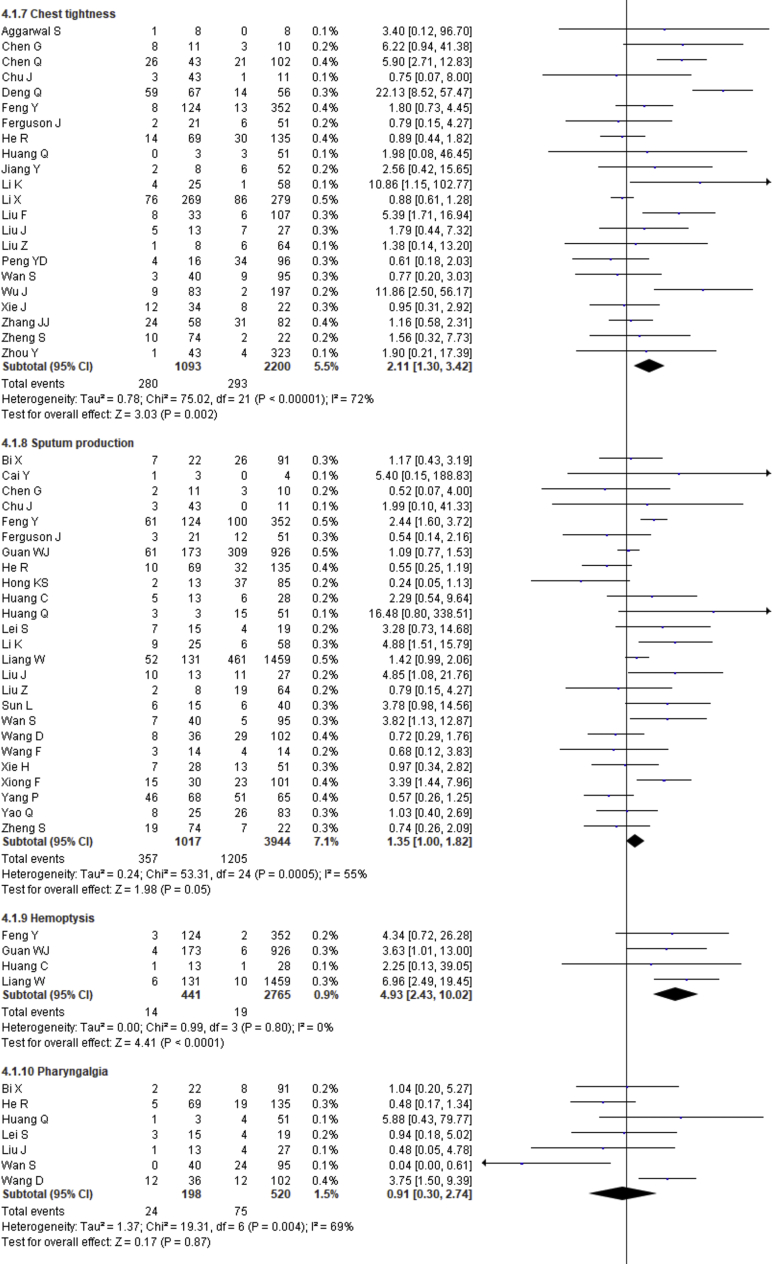

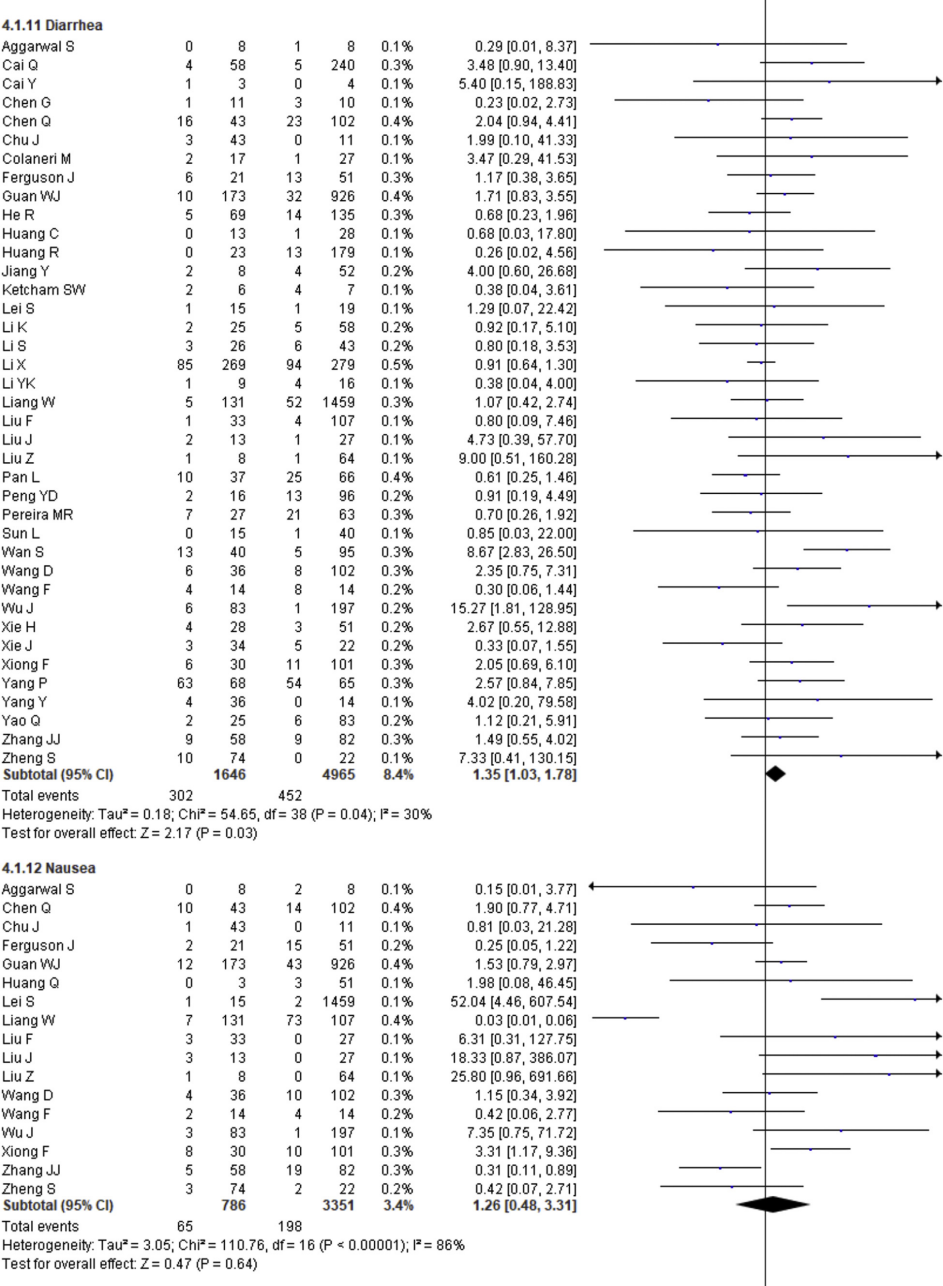

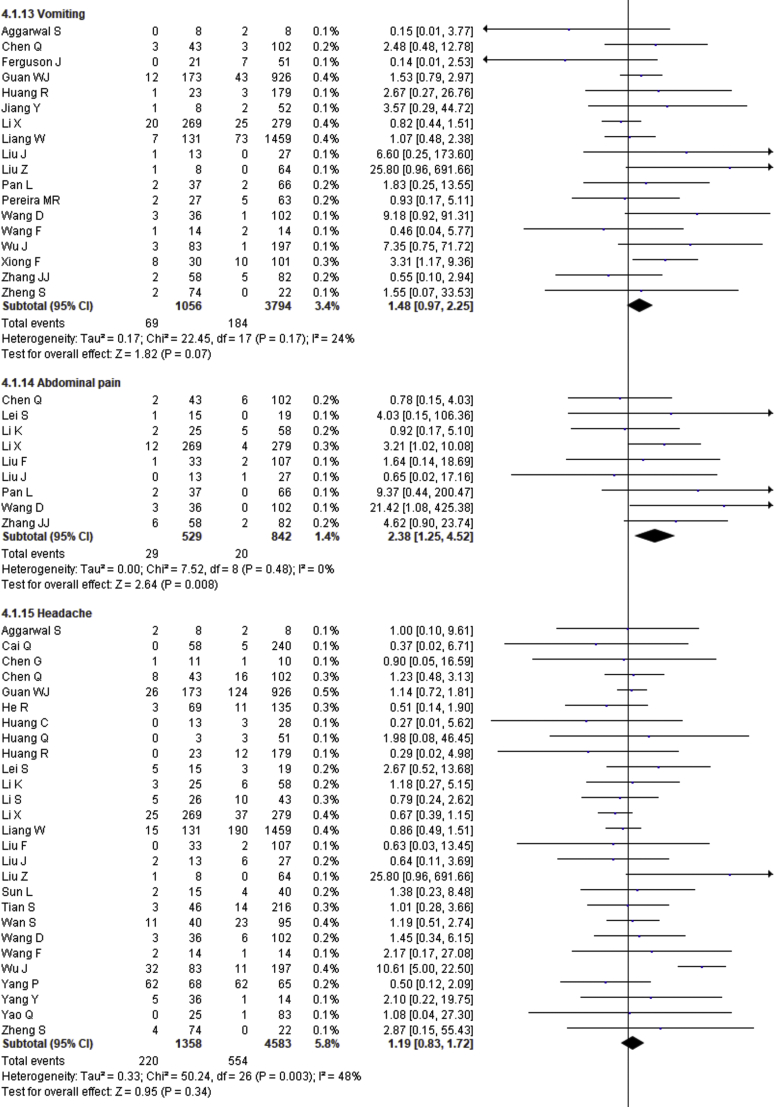

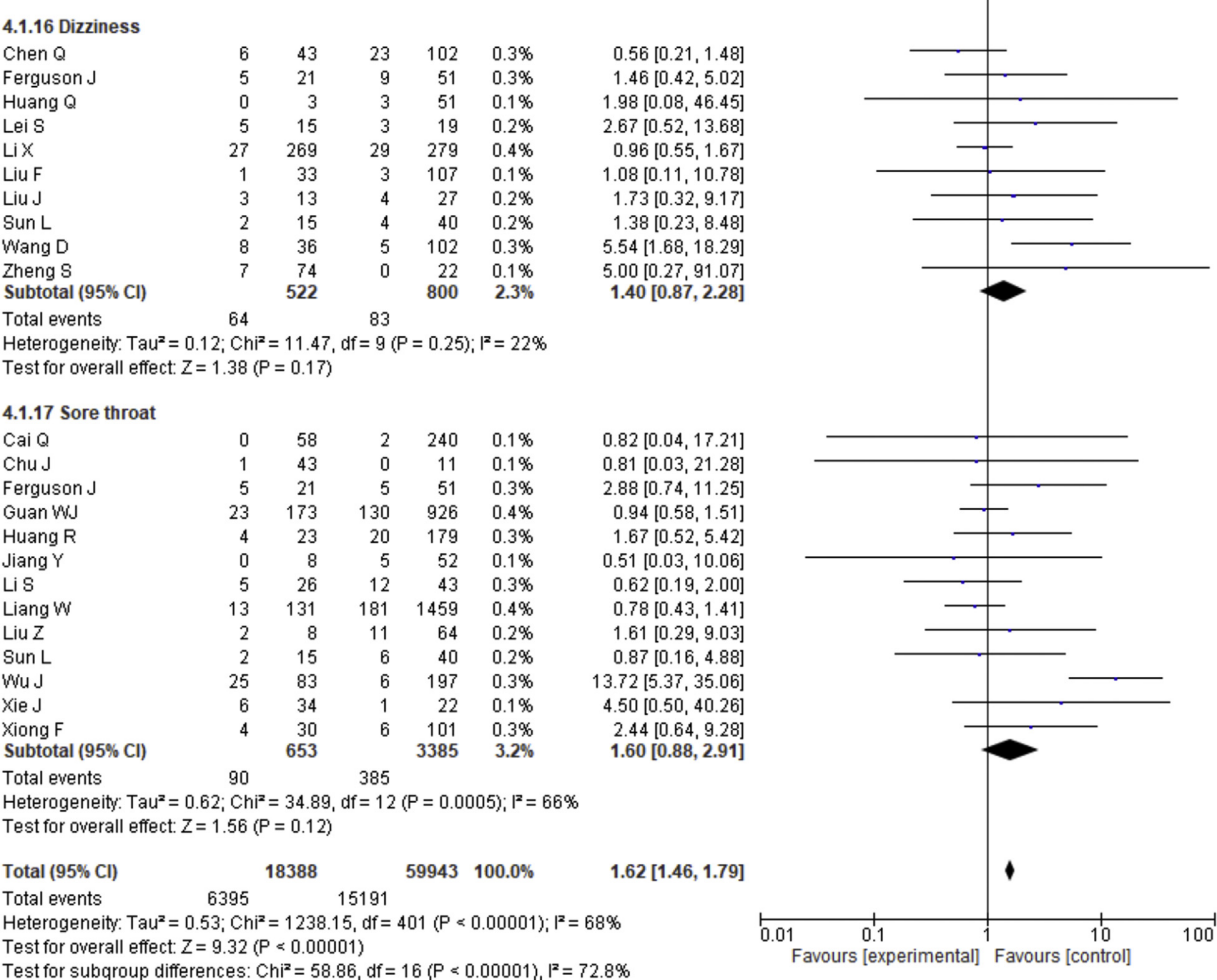
Meta-analysis for the effect of clinical symptoms on the severity of COVID-19 cases. Random effect model for fever, cough, fatigue, anorexia, myalgia, dyspnea, chest tightness, sputum production, hemoptysis, pharyngalgia, diarrhea, nausea, vomiting, abdominal pain, headache, dizziness and sore throat.

Fever, cough, fatigue, anorexia, myalgia, dyspnea, chest tightness, sputum production, hemoptysis, pharyngalgia, diarrhea, nausea, vomiting, abdominal pain, headache, dizziness and sore throat were reported in 47, 46, 37, 8, 34, 40, 22, 25, 4, 7, 39, 17, 18, 9, 27, 10 and 13 studies, respectively. The percentages of these symptoms in severe and nonsevere COVID-19 cases are presented in Figure 3 and Table 2, and forest plots are presented in Figure 9. The most prevalent clinical symptoms were fever (81.73%), cough (65.41%) and dyspnea (51.50%) followed by fatigue (38.34%), sputum production (35.10%), anorexia (31.23%), chest tightness (25.62%), myalgia (24.91%), diarrhea (18.35%), headache (16.20%), sore throat (13.78%), dizziness (12.26%), pharyngalgia (12.12%), nausea (8.27%), vomiting (6.53%), abdominal pain (5.48%) and hemoptysis (3.17) in the severe patients.

Regarding the clinical manifestations, fever (OR = 1.68, 95% CI = 1.29–2.19, p = 0.0001, I^2^ = 54%), cough (OR = 1.41, 95% CI = 1.11–1.77, p = 0.004, I^2^ = 63%), fatigue (OR = 1.26, 95% CI = 1.03–1.55, p = 0.03, I^2^ = 36%), anorexia (OR = 2.38, 95% CI = 1.60–3.54, p < 0.0001, I^2^ = 0%), dyspnea (OR = 4.30, 95% CI = 2.98–6.22, p < 0.00001, I^2^ = 79%), chest tightness (OR = 2.11, 95% CI = 1.30–3.42, p = 0.002, I^2^ = 72%), hemoptysis (OR = 4.93, 95% CI = 2.43–10.02, p < 0.0001, I^2^ = 0), diarrhea (OR = 1.35, 95% CI = 1.03–1.78, p = 0.03, I^2^ = 30%) and abdominal pain (OR = 2.38, 95% CI = 1.25–4.52, p = 0.008, I^2^ = 0%) are significantly associated with the severity of COVID-19 cases compared to nonsevere cases whereas myalgia (OR = 1.30, p = 0.07), pharyngalgia (OR = 0.91, p = 0.87), nausea (OR = 1.26, p = 0.64), vomiting (OR = 1.48, p = 0.07), headache (OR = 1.19, p = 0.34), dizziness (OR = 1.40, p = 0.17) and sore throat (OR = 1.60, p = 0.12) are not associated with increased risk of severity of COVID-19 cases and sputum production is in the marginal line (OR = 1.35, 95% CI = 1.00–1.82, p = 0.05, I^2^ = 55%).

### 3.6 Sensitivity and publication bias

Publication bias, checked by Egger's regression test and Begg-Mazumdar's rank correlation, are presented in Table 3 and Supplementary Figure S1–S28. No publication bias was found in case of age, sex, comorbidities and clinical symptoms tested by both Egger's and Begg-Mazumdar's tests (p > 0.05). The sensitivity was analyzed for assessing the stability of the results obtained and the influence of each study by omitting each study one by one for age, sex, comorbidities and clinical symptoms (Supplementary Figure S29-56). No significant effect of any single study on the pooled results was found in the case of age, sex and comorbidities and clinical symptoms.

## 4 Discussion

The new novel coronavirus (SARS-CoV-2) is the seventh human coronavirus, the third type of zoonotic coronavirus, and has genetically similarity with SARS-CoV (79%) and MERS-CoV (50%) [75, 76]. SARS-CoV-2 receptor-binding domain (RBD) is nearly the same as the RBD of SARS-CoV [77]. COVID-19 is highly contagious, and WHO declared it a global pandemic. A total of 5,962,944 confirmed cases and 363,905 (6.10%) deaths were reported as of May 29, 2020, and COVID-19 has spread to 213 countries and territories across all continents [14]. Moreover, China, Europe, and America sufferer more, and now, the severity decreases in China. Although the number of COVID-19 cases continues to grow worldwide, no specific antiviral treatment has been confirmed to be effective against COVID-19. So, clinical demographical characteristics, clinical manifestation, comorbidities of COVID-19 patients are more important to early detection and isolation as well as minimize the spread of the disease, severity, and death rate. In this meta-analysis, we retrospectively analyzed clinical data from patients with COVID-19. So, we completed a systemic meta-analysis. In this meta-analysis, we retrieved 55 independent studies from January 1, 2020, to May 24, 2020, which reported age, sex, severity, comorbidity, clinical symptoms, and different outcomes on 10014 patients with COVID-19 distributed across four countries.

In our study, we observed that males are more likely to be infected by COVID-19 and going to severe conditions (OR = 2.41, p < 0.00001) than females. A similar finding was also reported earlier in some other studies [78, 79]. A study conducted in Spain reported that men are more vulnerable than women because of their irresponsible attitude toward the risk of COVID-19 pandemic [80]. Another Spanish study revealed that the severity and case fatality rate (CFR) are higher in males and old aged people [81]. Moreover, a higher resistance in females is observed, which might be due to female sex hormones, whereas men have lower resistance because of high expression ACE2 receptor to which coronavirus binds easily [82]. Studies also showed that ACE2 expression, decreased B cell and NK cell-specific transcripts, male hormones, and increased NF-κB inhibitor are responsible for the higher viral load in men [83, 84, 85]. According to the data published by Global Health 50/50 presents that men are dying at a more consistent rate than women [86]. Besides, the lifestyle of men, including smoking, leads to high viral load and high severity [87]. A systemic review and meta-analysis also suggested that current smokers are at greater risk than former or non-smokers [88]. One study reported a positive hazard ratio (HR) in the COVID-19 related deaths for current smokers (HR = 1.14, 95%Cl = 1.05–1.23) in a model adjusted for demographic age and sex, whereas a lower HR was found in a fully adjusted model (HR = 0.89, 95%Cl = 0.82–0.97) [89].

Elderly or older people in both sexes (≥50 years) are more susceptible to SARS-CoV-2, which may be associated with a higher frequency of severity (age≥50 years vs. age<50 years, RR = 3.36; 95% CI = 1.79–6.30, p = 0.0002). We did not find any significant association of age ≥65 years with COVID-19 severity. Although we did not find any association of age (≥65 years) with COVID-19 severity, the age ≥50 years group also included some patients with ≥65 years of age. It was thought that elderly or older people are more susceptible to severity for weak immunity and other organ dysfunction. Elderly or older people and a higher frequency of comorbidities patients are more susceptible to SARS-CoV-2 [78,90].

Among 10014 COVID-19 patients, 51.14% had at least one comorbidity in severe groups, and other most common comorbidities in severe cases are hypertension (36.47%), diabetes (21.19%), cardiovascular disease (18.76%), cerebrovascular disease (11.83%) and chronic kidney disease (10.63%). All the preexisting comorbidities are associated with the increased severity in the COVID-19 cases (p < 0.05) in our current meta-analysis. Any comorbidity is a crucial factor in poor prognosis. Diseases such as hypertension, diabetes, respiratory system disease, cardiovascular disease, and their susceptibility conditions are higher risk of severe illness or death [78, 91, 92, 93]. Some articles also reported an association of hypertension and other cardiovascular diseases with COVID-19 [94,95]. Innate immunity response, macrophage, and lymphocyte function are decreased in the presence of comorbidities, which may be more susceptible to the pathogenesis of COVID-19 [96]. A metabolic disorder, inflammation, and infection are induced by diabetes, whereas chronic liver disease was reported to be associated with COVID-19 [97,98]. The presence of respiratory diseases develops acute respiratory distress syndromes (ARDS). Furthermore, a study reported that diabetes, smoking, and heart disease were mainly responsible for MERS-CoV illness [99]. The expression of ACE2 receptors is increased in some comorbid conditions like hypertension and diabetes, and SARS-CoV-2 attacks cells through ACE2 receptors. Therefore, comorbidities increase the severity of COVID-19 cases [90].

We summarized 17 clinical symptoms in our meta-analysis among them we found the significant association of fever (81.73%, OR = 1.68, p = 0.0001), cough (65.41%, OR-1.41, p = 0.004), fatigue (38.34%, OR = 1.26, p = 0.03), anorexia (31.23%, OR = 2.38, p < 0.0001), dyspnea (51.50%, OR = 4.30, p < 0.00001), chest tightness (25.62%, OR = 2.11, p = 0.002), hemoptysis (3.17%, OR = 4.93, p < 0.0001), diarrhea (18.35%, OR = 1.35, p = 0.03), abdominal pain (5.48%, OR = 2.38, p = 0.008) with the severity of COVID-19 cases.

We observed no association of COVID-19 severity with myalgia (24.91%, OR = 1.30, p = 0.07), sputum production (35.10%, OR = 1.35, p = 0.05), pharyngalgia (12.12%, OR = 0.91, p = 0.87), nausea (8.27%, OR = 1.26, p = 0.64), vomiting (6.53%, OR = 1.48, p = 0.07), headache (16.20%, OR = 1.19, p = 0.34), dizziness (12.26%, OR = 1.40, p = 0.17), and sore throat (13.78%, OR = 1.60, p = 0.12). SARS-CoV-2 binds with the ACE-2 receptor, causing diffuse alveolar damage and lymphocytic infiltration in both lungs and may cause several respiratory tract symptoms [100]. Several clinical researchers found that the common clinical manifestations of COVID-19 patients are fever, cough, headache, fatigue, myalgia, nausea, diarrhea, and sputum [101]. Diarrhea has been found in the Middle East respiratory syndrome coronavirus (MERS-COV) patients (up to 30%) [102]. A recent study showed that SARS-CoV-2 was detected in stool samples of patients with abdominal symptoms [103]. There also found expression of SARS-CoV2 receptor in the GI tract that may be related to GI-related symptoms like diarrhea, nausea and vomiting [104]. Shortness of breath or dyspnea indicates an impaired function of the lung and oxygen deficiency. Therefore, while planning to pay great attention to patients with the respiratory system and dyspnea as the primary symptoms, more attention should also be given to patients with cough, fatigue, anorexia, chest tightness, hemoptysis, diarrhea, abdominal pain, headaches, dizziness, nausea, sputum production and vomiting [78, 79, 105, 106].

Nowadays, many articles have been published on epidemiologic and clinical characteristics, but variations in reporting descriptive data may lead to the misunderstanding of the clinical features of COVID-19. Besides, some meta-analysis is also published. However, these meta-analyses pooled a small number of studies (<30). This is the first meta-analysis with the various studies (55 citations) and the most detailed review and clear proof of the clinical characteristics of COVID-19 patients to date. The quality of the publications included in this study is high, the analysis is rigorous, comprehensive, and the conclusions drawn by this meta-analysis are highly credible. Although this is a novel meta-analysis, there were some limitations to our study. First, the studies included were retrospective. Second, the sample size (7–1590) has a considerable variation among the included studies, leading to high heterogeneity. Third, reports being restricted to China and a few other countries, and our goal is to use the findings of this study to predict patients in general, including other countries and races. Without this limitation, this study analyzed the risk factors for progression to critical illness in COVID-19 patients to help to assess patient status and identify critical patients early. Our findings provide valuable information regarding the association of age, sex, comorbidities and clinical symptoms with the severity of COVID-19. We hope this information will support health care professionals and decision-makers in the current global pandemic, and more caution, as well as better early intervention, should be taken to improve the prognosis for older patients with respiratory failure. Effective treatment measures should be taken according to age, sex, comorbidities and clinical symptoms as the severity of COVID-19 is associated with these parameters.

## 5 Conclusions

Nowadays, COVID-19 is an emerging infectious disease and led to a significant health concern globally. Our study found that male patients and elderly or older patients (age≥50 years) are at a higher risk of developing disease severity. Our study also suggests that the presence of at least one or combined comorbidities like hypertension, diabetes, cerebrovascular disease, cardiovascular diseases, respiratory disease, malignancy, chronic kidney disease and chronic liver disease increases the severity of COVID-19. The prevalence of most common clinical symptoms like fever, cough, fatigue, anorexia, dyspnea, chest tightness, hemoptysis, diarrhea and abdominal pain were significantly higher in severe patients, and these are associated with the disease severity. This meta-analysis will help health care providers make appropriate medical decisions for their patients based on age, sex, comorbidities and clinical symptoms.

## Declarations

### Author contribution statement

All authors listed have significantly contributed to the development and the writing of this article.

### Funding statement

This research did not receive any specific grant from funding agencies in the public, commercial, or not-for-profit sectors.

### Data availability statement

Data included in article/supplementary material.

### Competing interest statement

The authors declare no conflict of interest.

### Additional information

No additional information is available for this paper.

## Appendix A Supplementary data

Supplimentary materials_revised Supplimentary materials_revised
