# Supplementary material for: Impact of age, sex, comorbidities and clinical symptoms on the severity of COVID-19 cases: A meta-analysis with 55 studies and 10014 cases
Source: Heliyon. 2020 Dec 15;6(12):e05684. doi: 10.1016/j.heliyon.2020.e05684 (PMC7737518; doi:10.1016/j.heliyon.2020.e05684)
Supplement: Supplimentary materials_revised [file mmc1.docx]

**Impact of age, sex, comorbidities and clinical symptoms on the severity of COVID-19 cases: a meta-analysis with 55 studies and 10014 cases**

Md. Abdul Barek, Md. Abdul Aziz, Mohammad Safiqul Islam

**The file includes:**

**Supplemental Figures S1-S56**

**Supplemental Tables S1**


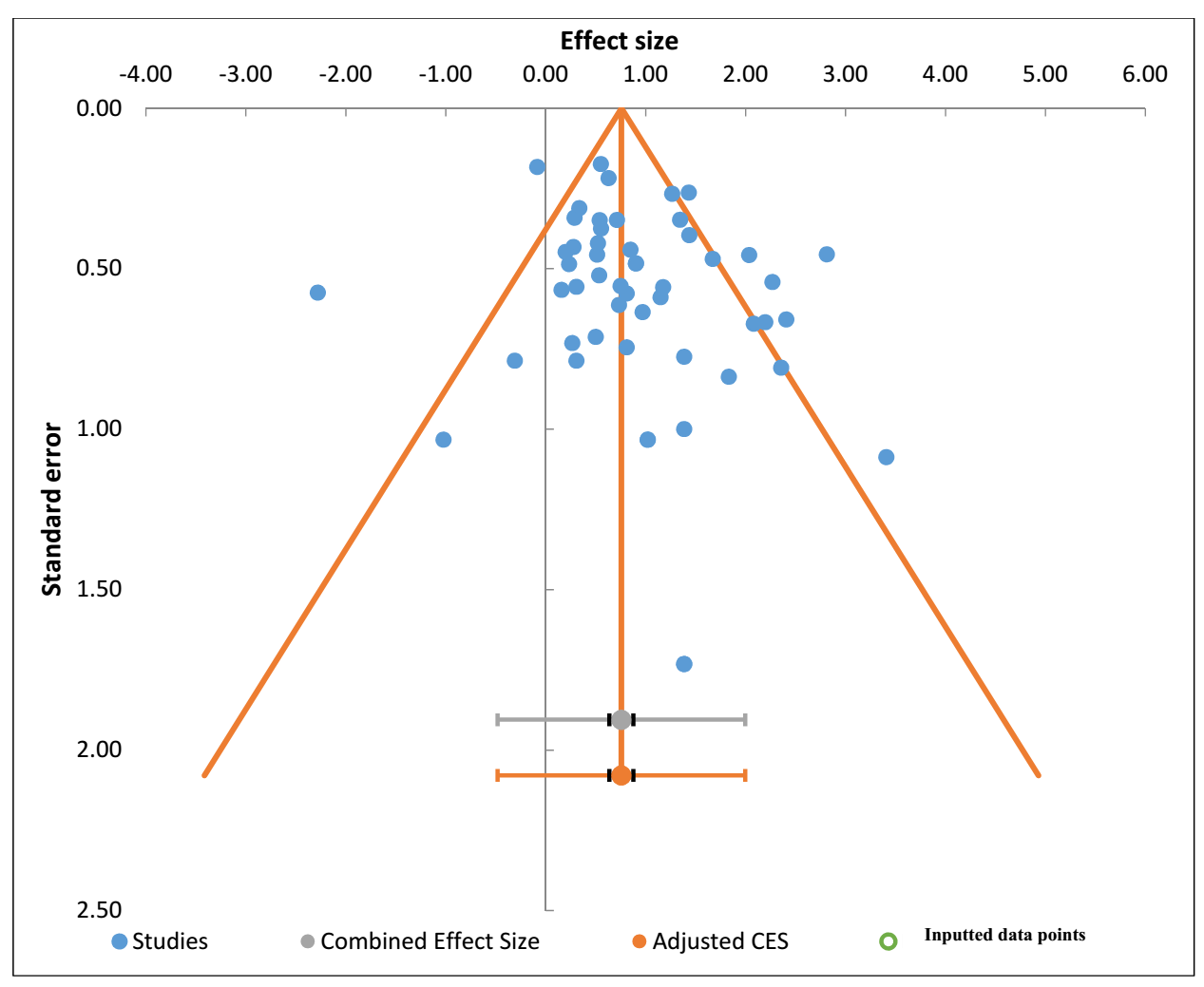


**Supplemental Figure S1**. Funnel plots for analysis of the effect of sex on the severity of the COVID-19 cases. To identify the possible publication bias funnel plot with combined effect size and adjusted combined effect size accompanying confidence and prediction intervals were generated. The odds ratios are plotted against the standard error for the indicated sex indicators.


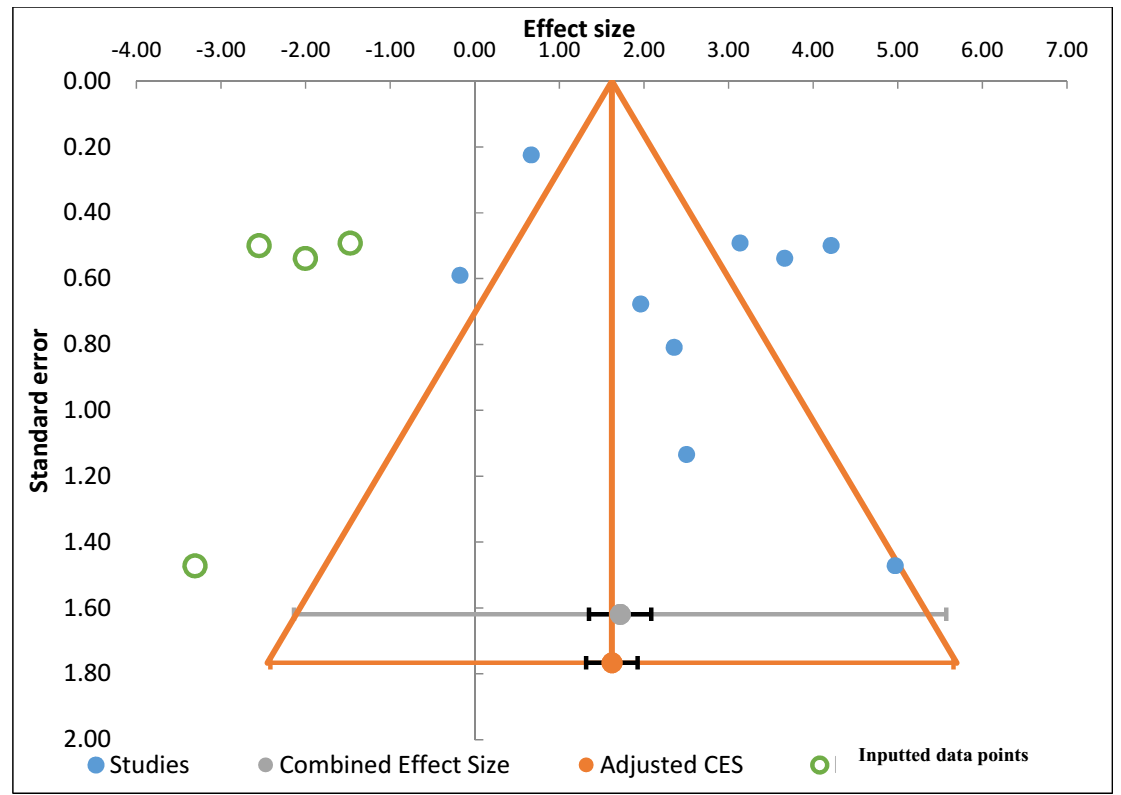


A


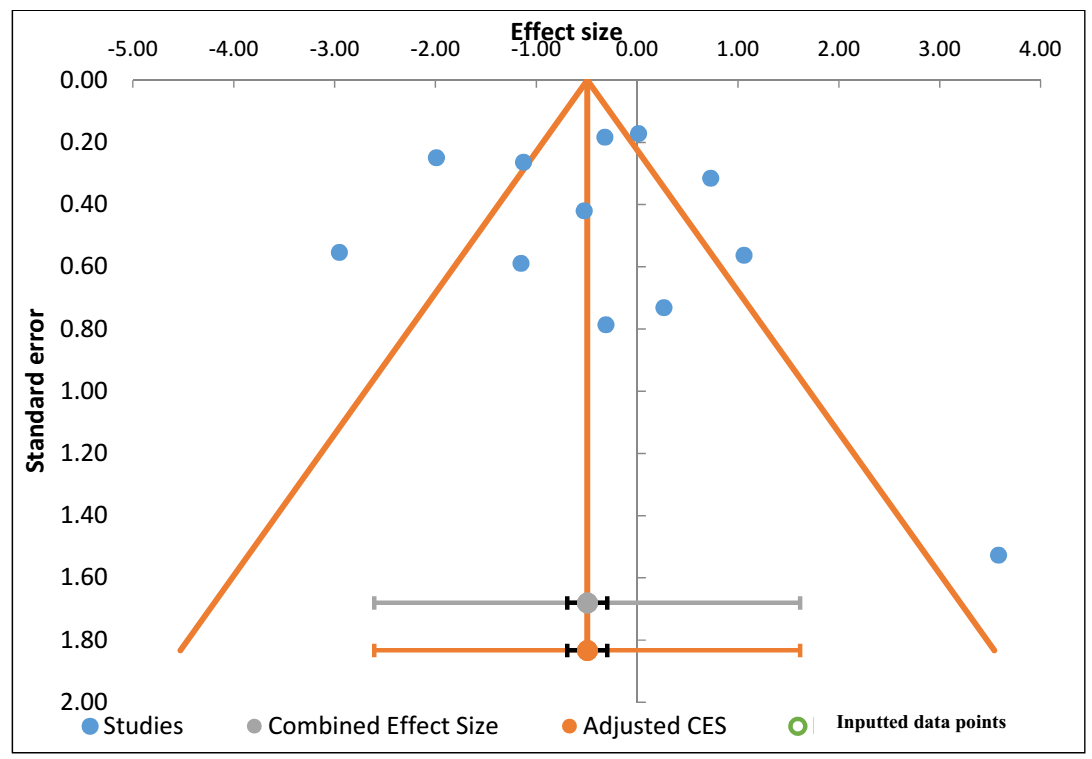


B

**Supplemental Figure S2**. Funnel plots for analysis of the effect of age on the severity of the COVID-19 cases: A. Age (≥50 vs. age<50), B. Age (≥65 vs. age<65). To identify the possible publication bias funnel plot with combined effect size and adjusted combined effect size accompanying confidence and prediction intervals were generated. The odds ratios are plotted against the standard error for the indicated age indicators


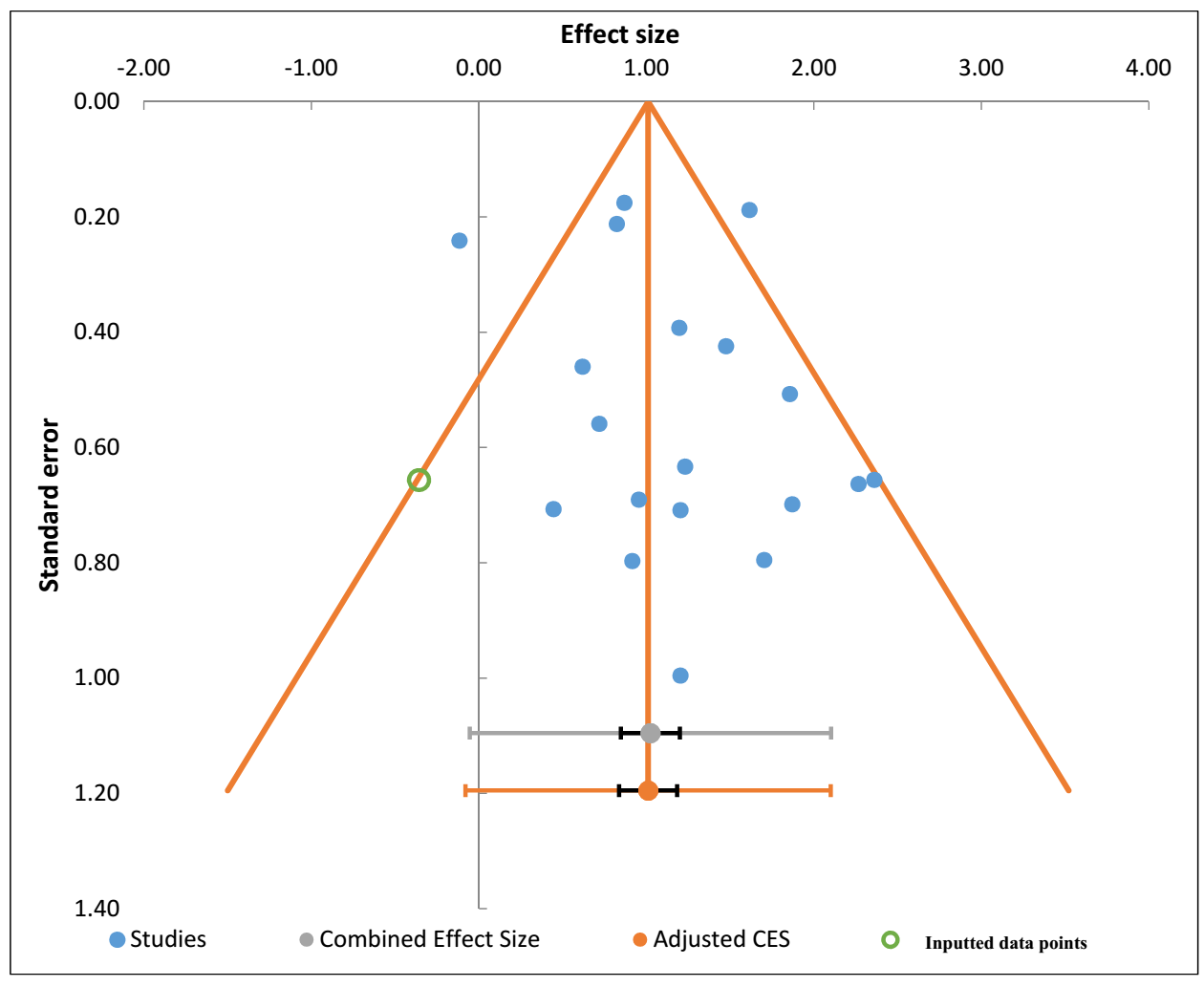


**Supplemental Figure S3**. Funnel plots for analysis of the effect of any comorbidity on the severity of the COVID-19 cases. To identify the possible publication bias funnel plot with combined effect size and adjusted combined effect size accompanying confidence and prediction intervals were generated. The odds ratios are plotted against the standard error for the indicated any comorbidity indicators


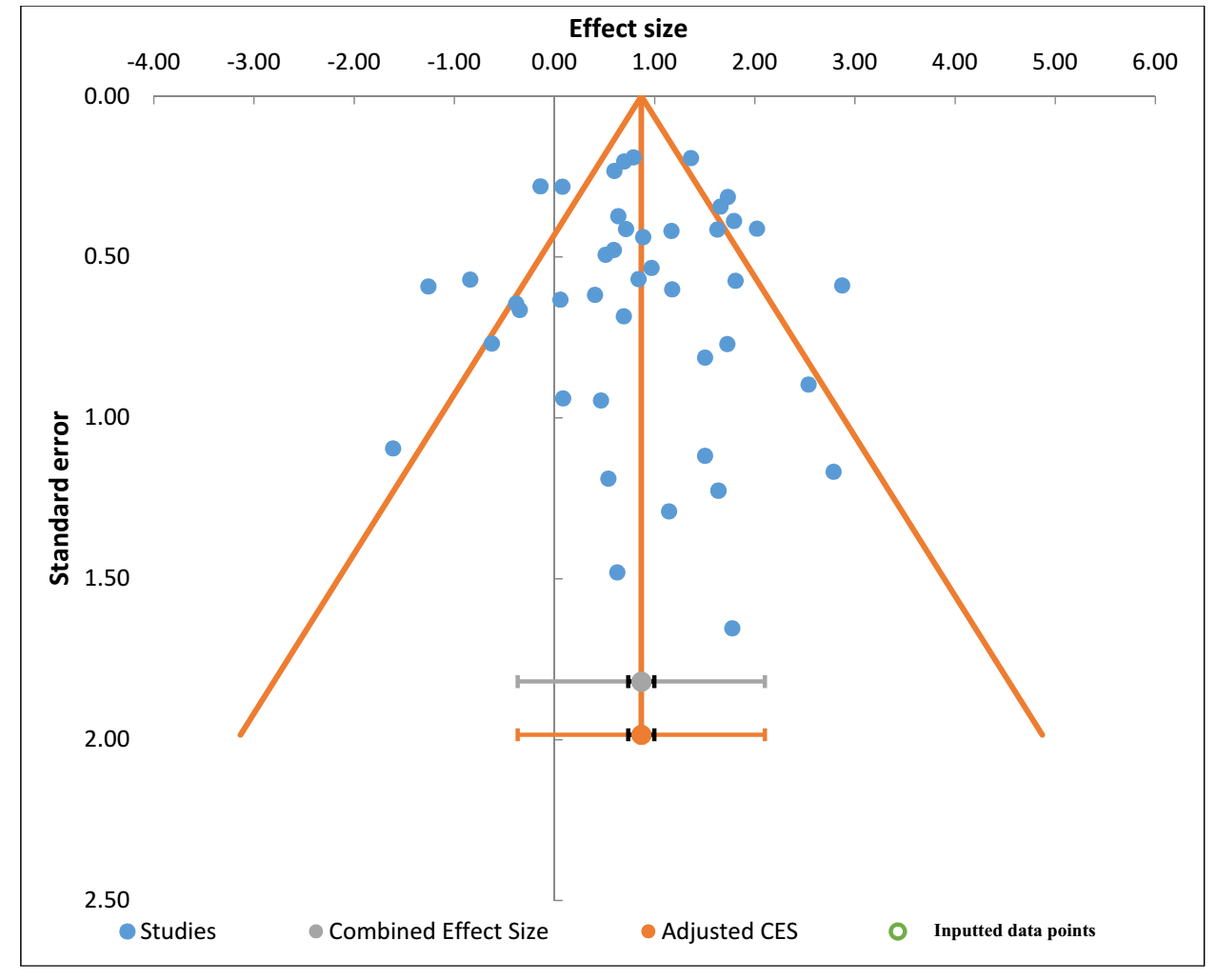


**Supplemental Figure S4**. Funnel plots for analysis of the effect of hypertension on the severity of the COVID-19 cases. To identify the possible publication bias funnel plot with combined effect size and adjusted combined effect size accompanying confidence and prediction intervals were generated. The odds ratios are plotted against the standard error for the indicated hypertension indicators.


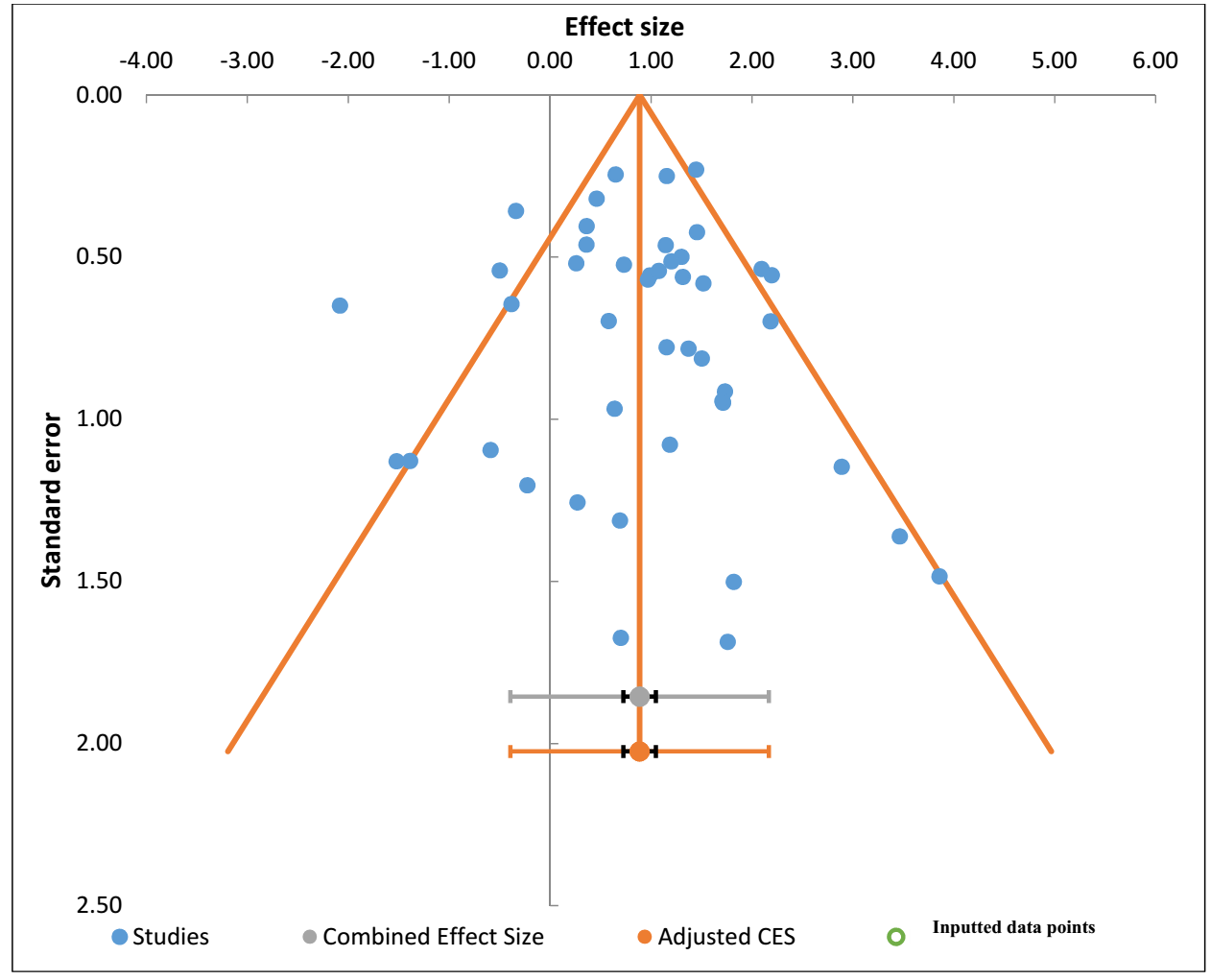


**Supplemental Figure S5**. Funnel plots for analysis of the effect of diabetes on the severity of the COVID-19 cases. To identify the possible publication bias funnel plot with combined effect size and adjusted combined effect size accompanying confidence and prediction intervals were generated. The odds ratios are plotted against the standard error for the indicated diabetes indicators.


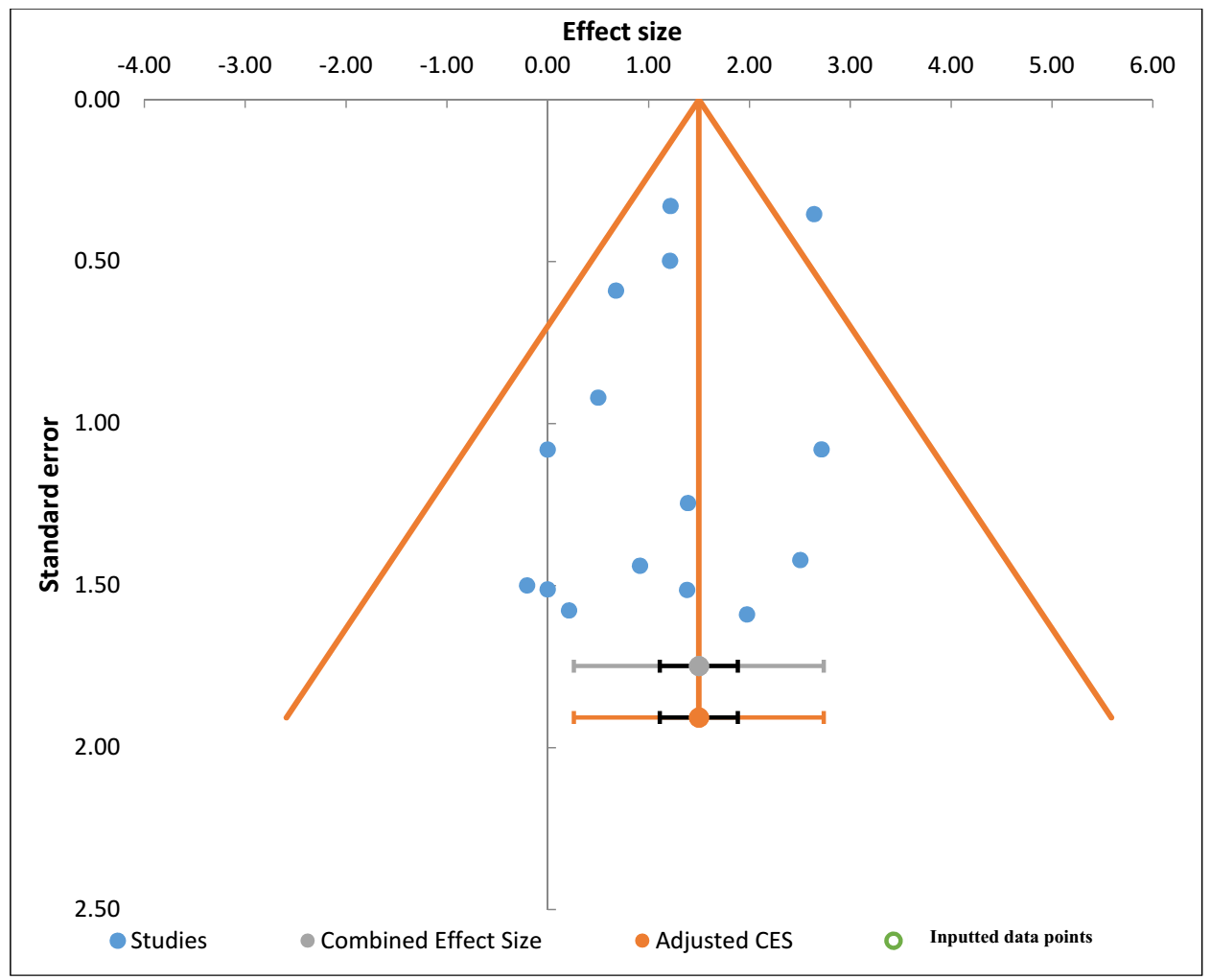


**Supplemental Figure S6**. Funnel plots for analysis of the effect of cerebrovascular disease on the severity of the COVID-19 cases. To identify the possible publication bias funnel plot with combined effect size and adjusted combined effect size accompanying confidence and prediction intervals were generated. The odds ratios are plotted against the standard error for the indicated cerebrovascular disease indicators.


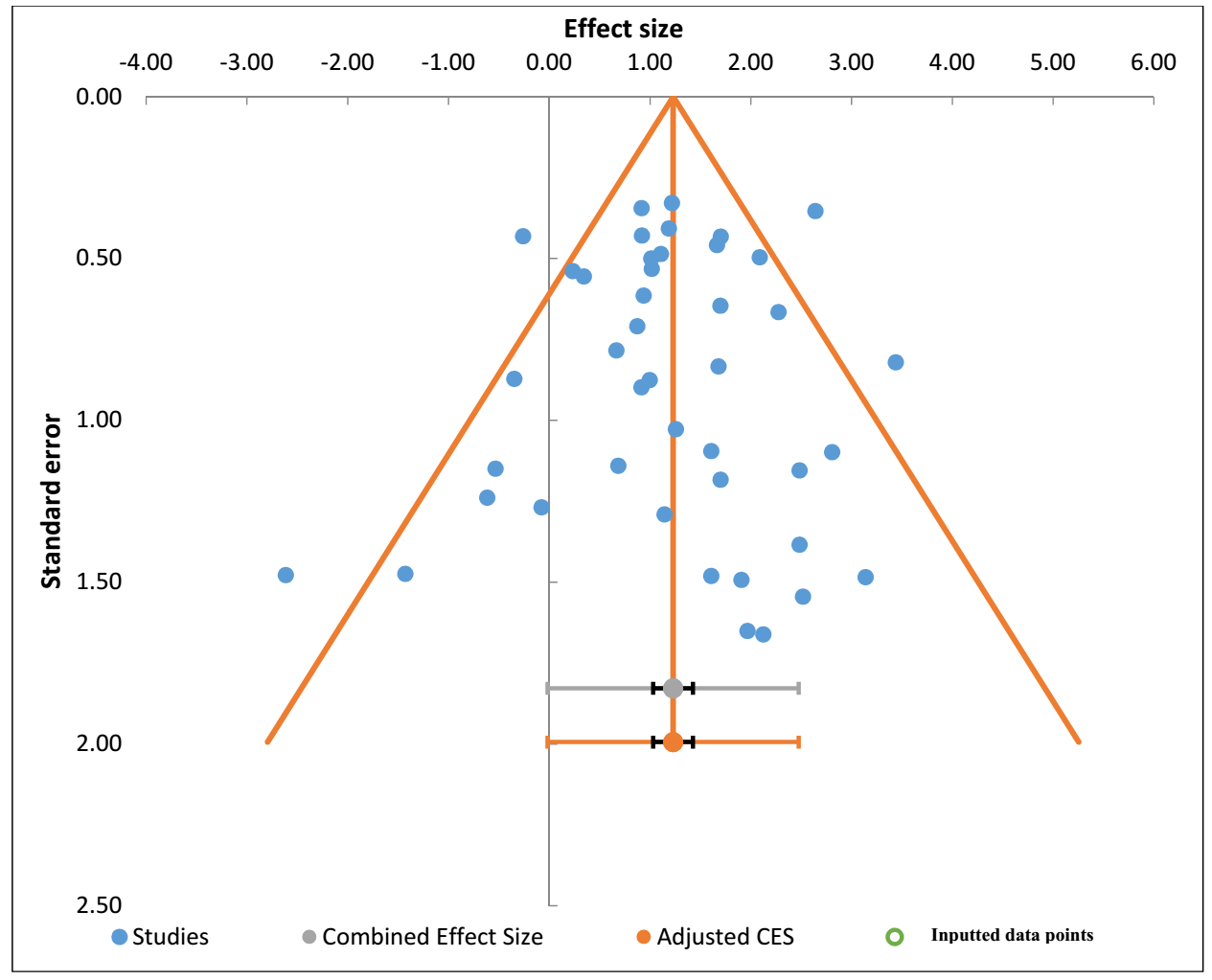


**Supplemental Figure S7.** Funnel plots for analysis of the effect of cardiovascular disease on the severity of the COVID-19 cases. To identify the possible publication bias funnel plot with combined effect size and adjusted combined effect size accompanying confidence and prediction intervals were generated. The odds ratios are plotted against the standard error for the indicated cardiovascular disease indicators.


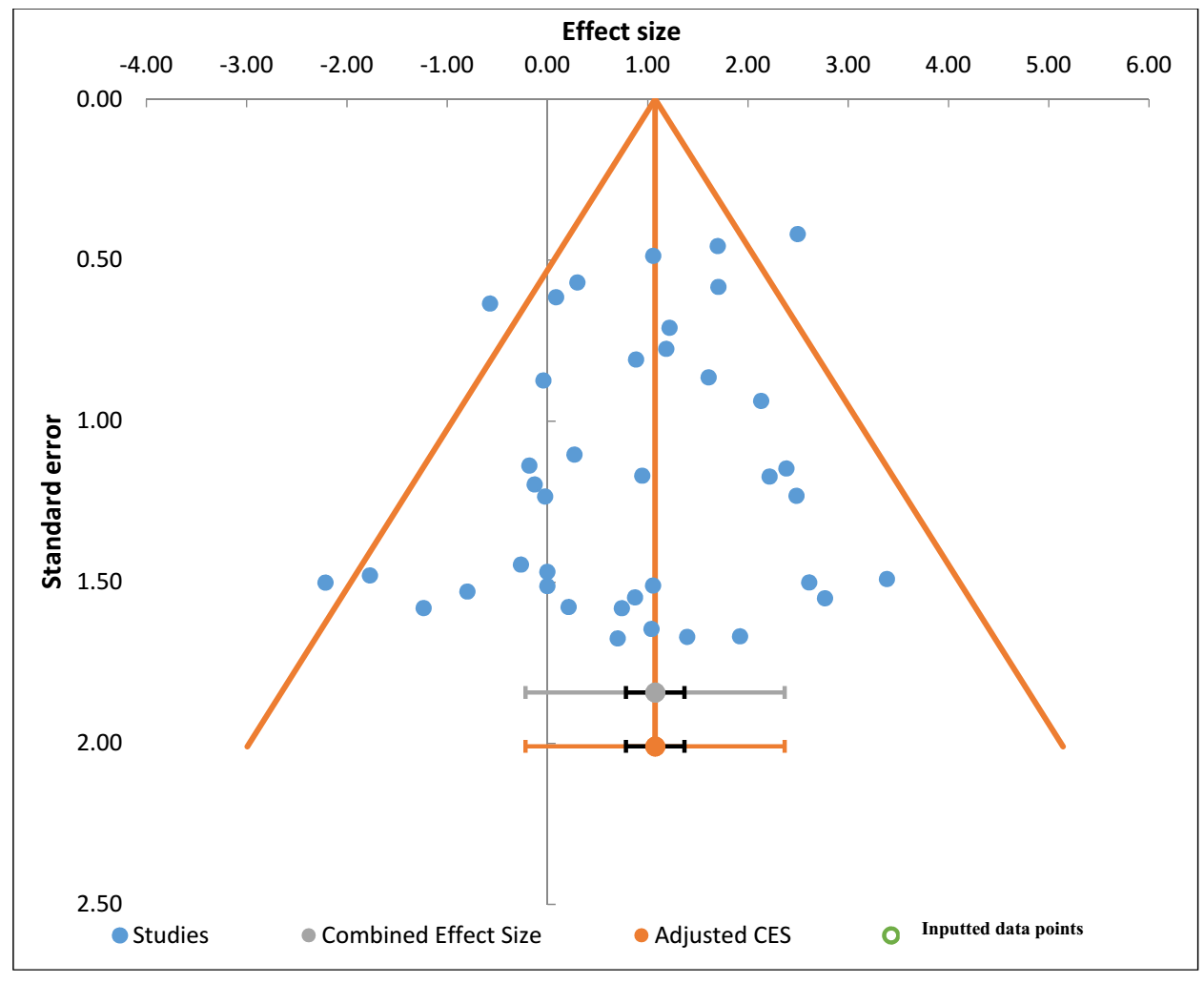


**Supplemental Figure S8**. Funnel plots for analysis of the effect of respiratory disease on the severity of the COVID-19 cases. To identify the possible publication bias funnel plot with combined effect size and adjusted combined effect size accompanying confidence and prediction intervals were generated. The odds ratios are plotted against the standard error for the indicated respiratory disease indicators.


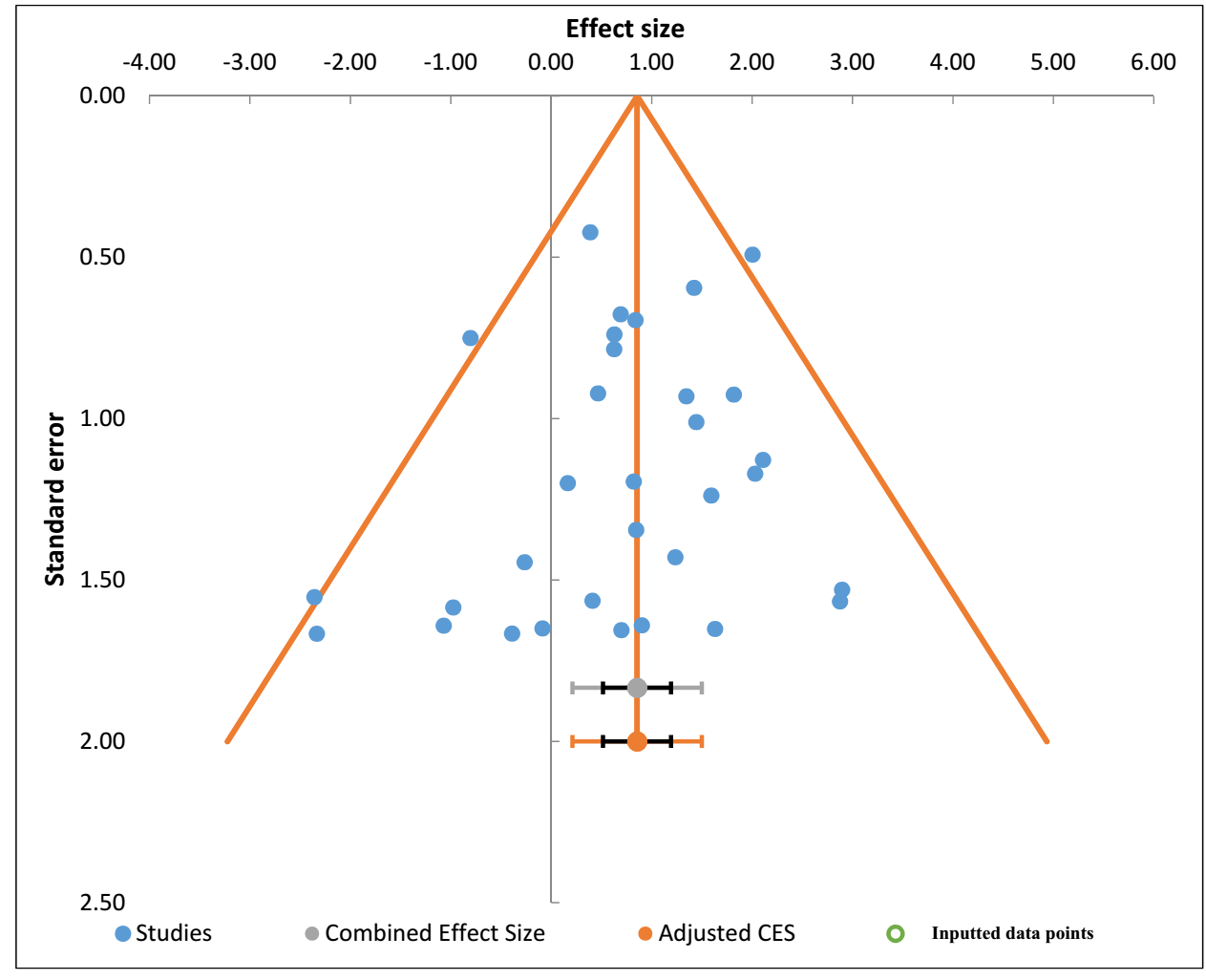


**Supplemental Figure S9**. Funnel plots for analysis of the effect of malignancy on the severity of the COVID-19 cases. To identify the possible publication bias funnel plot with combined effect size and adjusted combined effect size accompanying confidence and prediction intervals were generated. The odds ratios are plotted against the standard error for the indicated malignancy indicators.


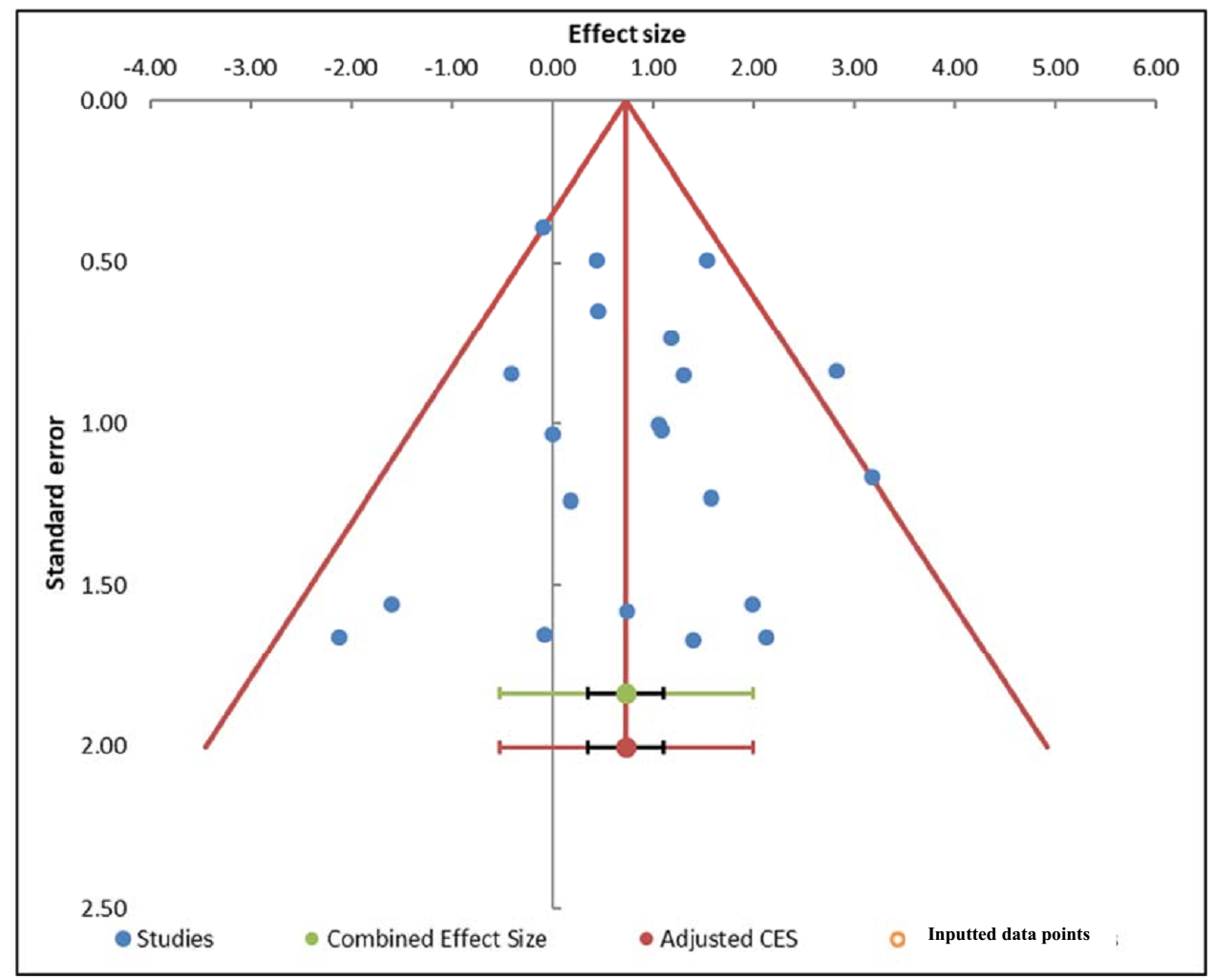


**Supplemental Figure S10.** Funnel plots for analysis of the effect of chronic kidney disease on the severity of the COVID-19 cases. To identify the possible publication bias funnel plot with combined effect size and adjusted combined effect size accompanying confidence and prediction intervals were generated. The odds ratios are plotted against the standard error for the indicated chronic kidney disease indicators.


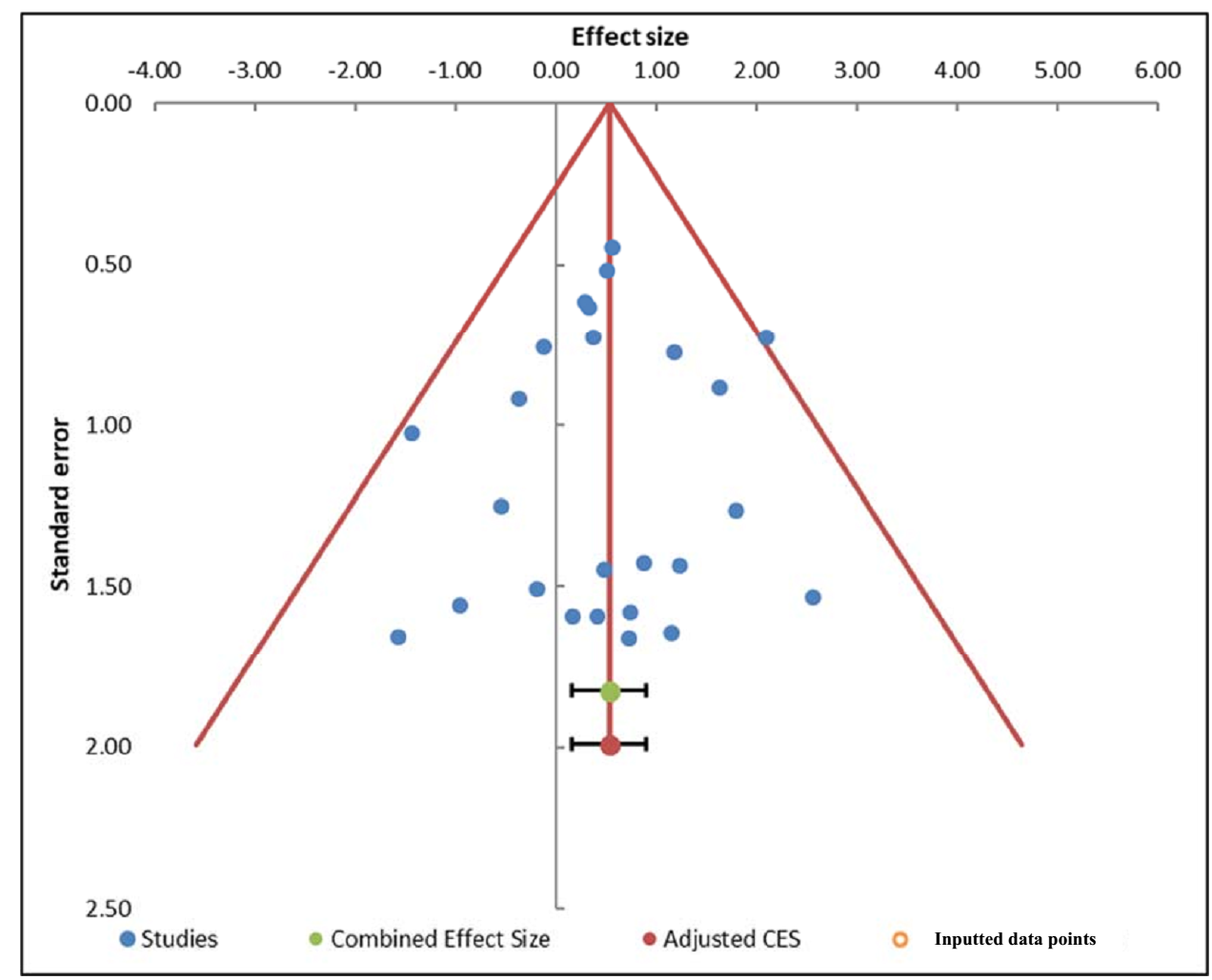


**Supplemental Figure S11.** Funnel plots for analysis of the effect of chronic liver disease on the severity of the COVID-19 cases. To identify the possible publication bias funnel plot with combined effect size and adjusted combined effect size accompanying confidence and prediction intervals were generated. The odds ratios are plotted against the standard error for the indicated chronic liver disease indicators.


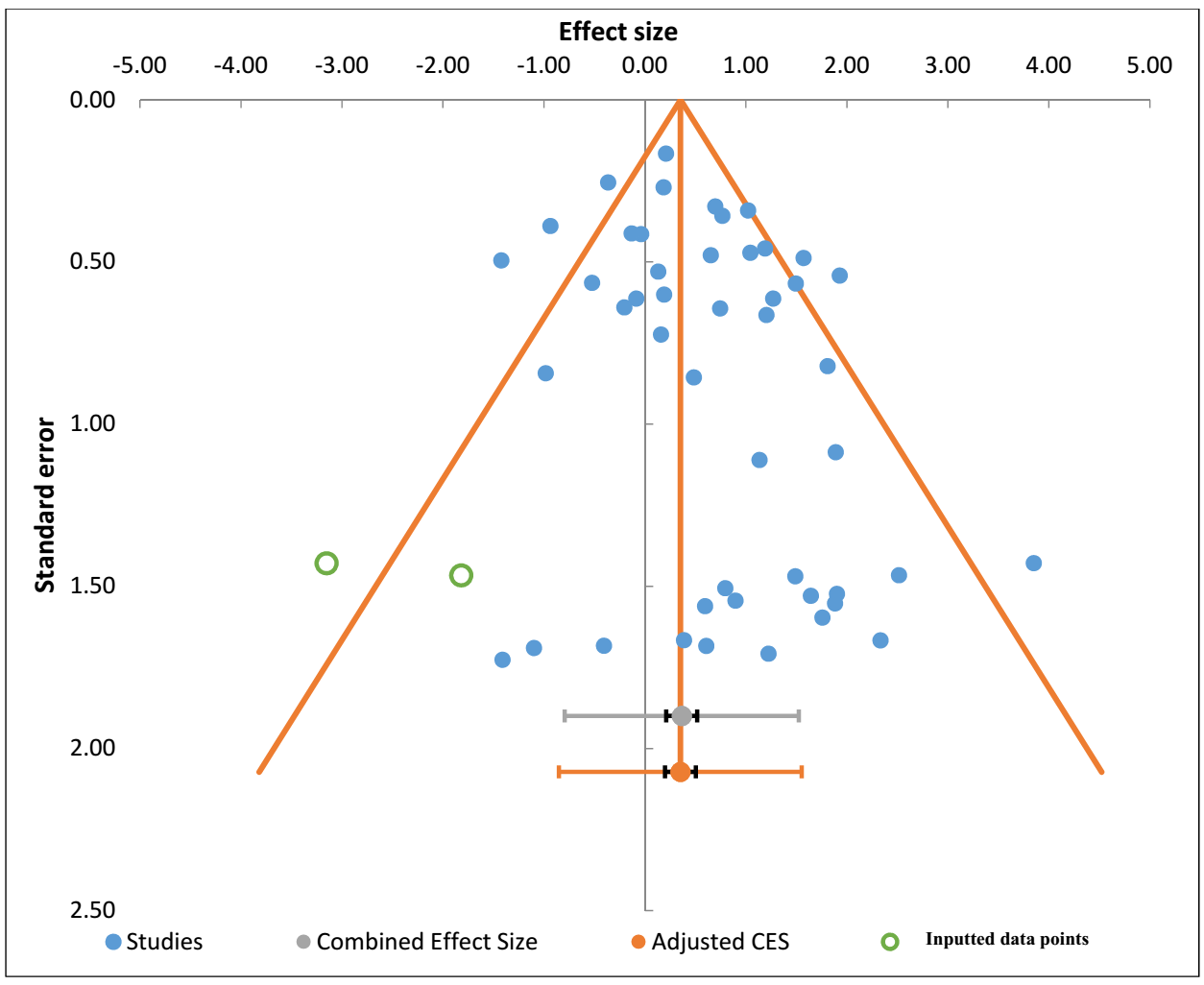


**Supplemental Figure S12.** Funnel plots for analysis of the effect of fever on the severity of the COVID-19 cases. To identify the possible publication bias funnel plot with combined effect size and adjusted combined effect size accompanying confidence and prediction intervals were generated. The odds ratios are plotted against the standard error for the indicated fever indicators.


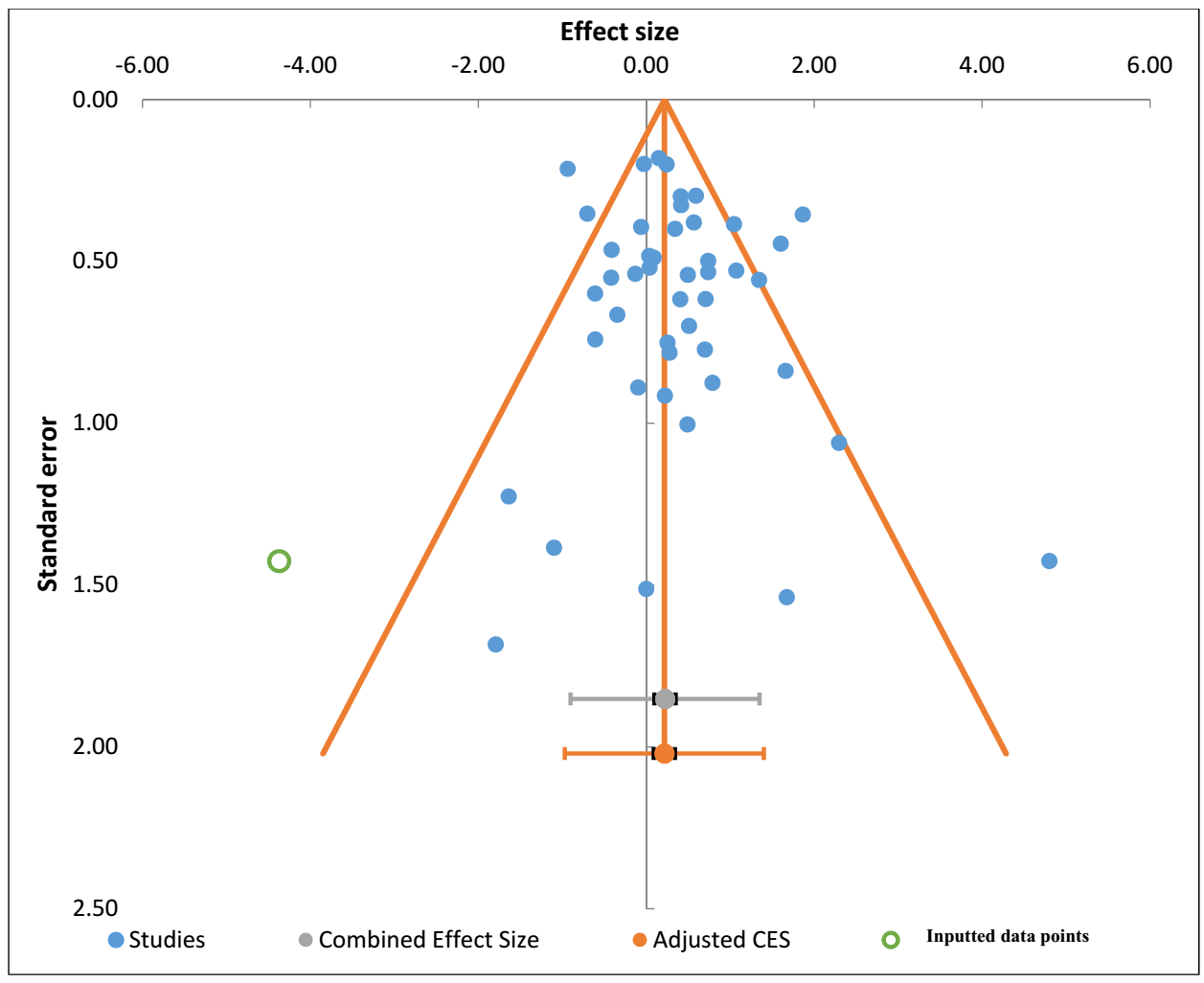


**Supplemental Figure S13.** Funnel plots for analysis of the effect of cough on the severity of the COVID-19 cases. To identify the possible publication bias funnel plot with combined effect size and adjusted combined effect size accompanying confidence and prediction intervals were generated. The odds ratios are plotted against the standard error for the indicated cough indicators.

**_Inputted data points_**


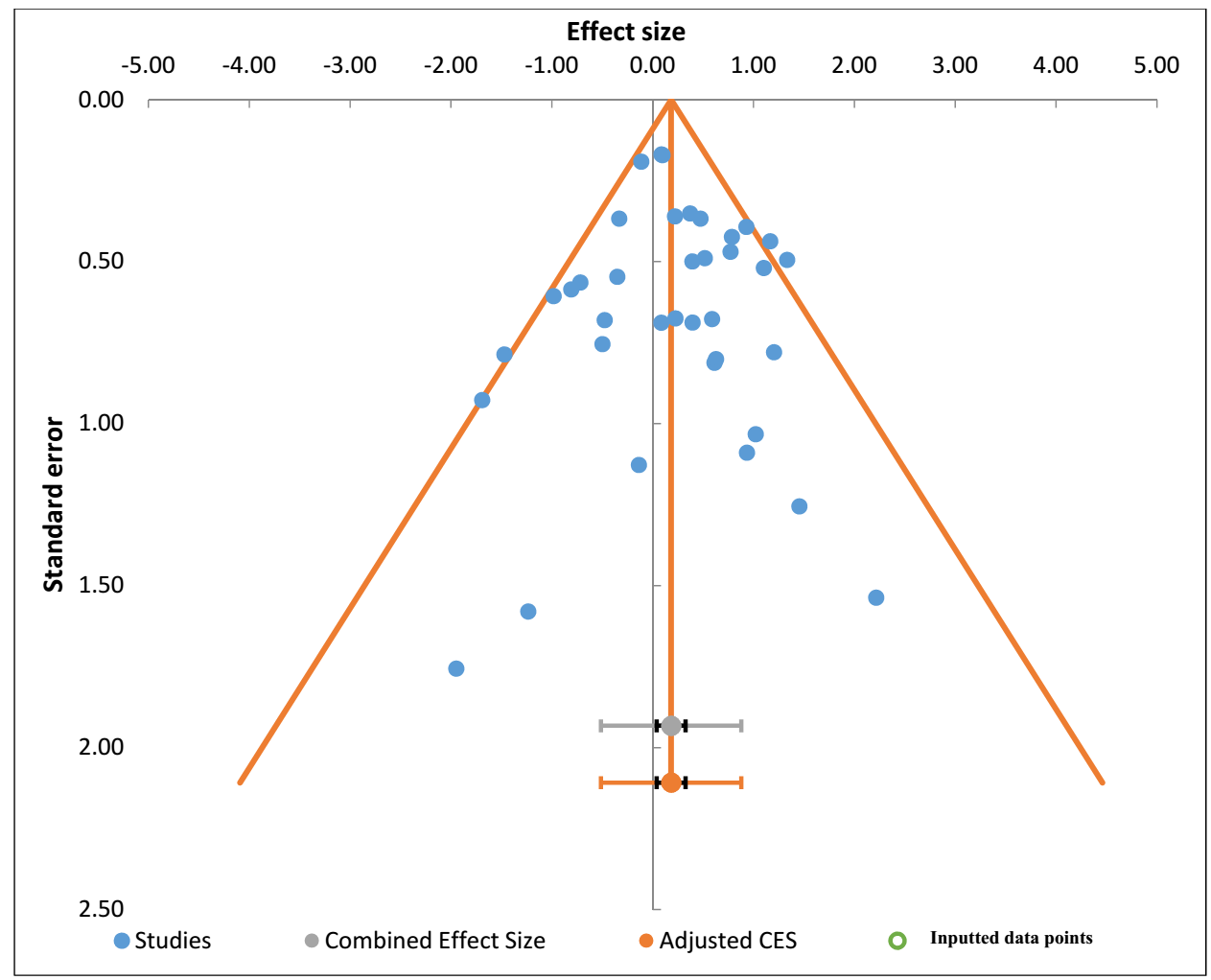


**Supplemental Figure S14.** Funnel plots for analysis of the effect of fatigue on the severity of the COVID-19 cases. To identify the possible publication bias funnel plot with combined effect size and adjusted combined effect size accompanying confidence and prediction intervals were generated. The odds ratios are plotted against the standard error for the indicated fatigue indicators.


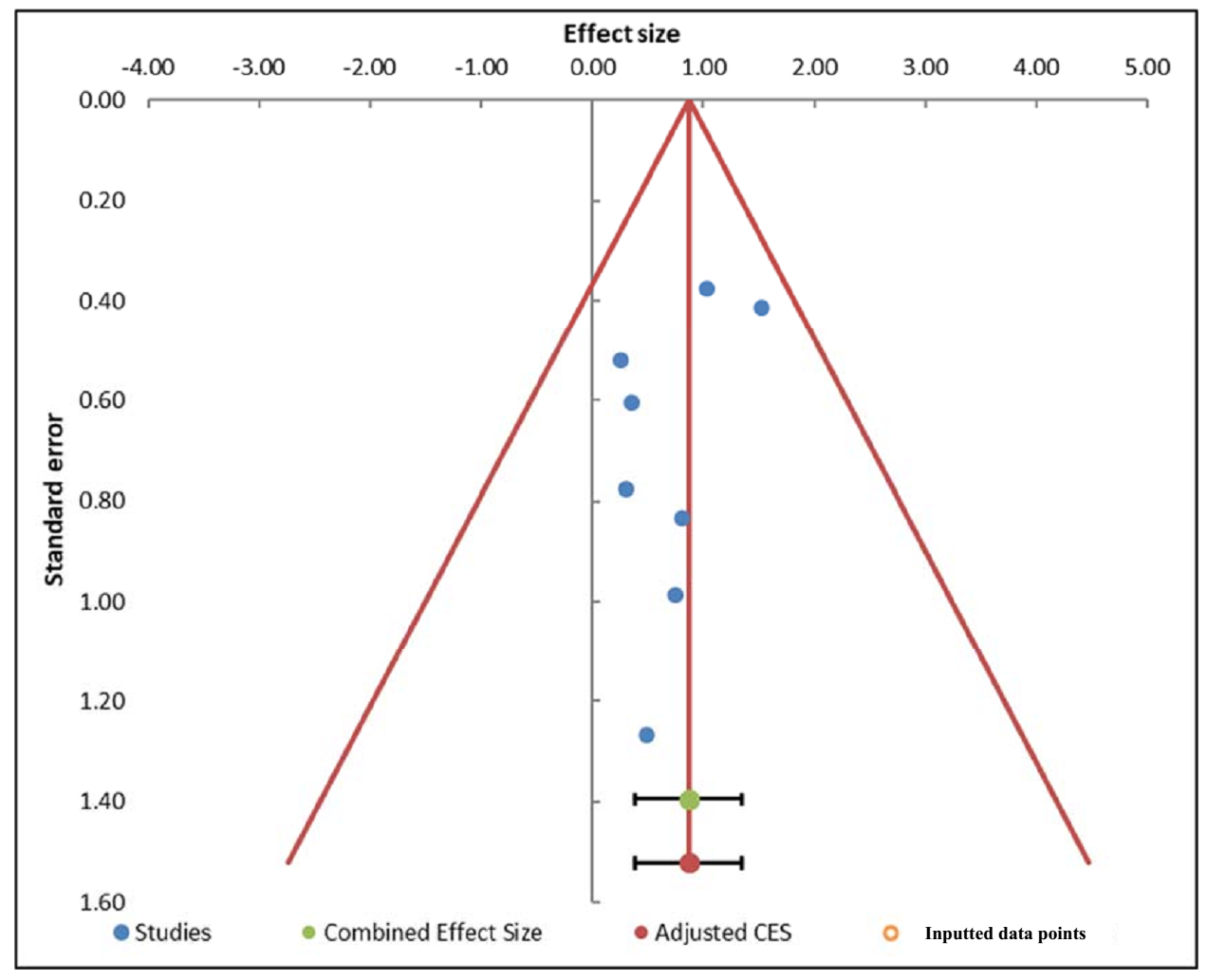


**Supplemental Figure S15.** Funnel plots for analysis of the effect of anorexia on the severity of the COVID-19 cases. To identify the possible publication bias funnel plot with combined effect size and adjusted combined effect size accompanying confidence and prediction intervals were generated. The odds ratios are plotted against the standard error for the indicated anorexia indicators.


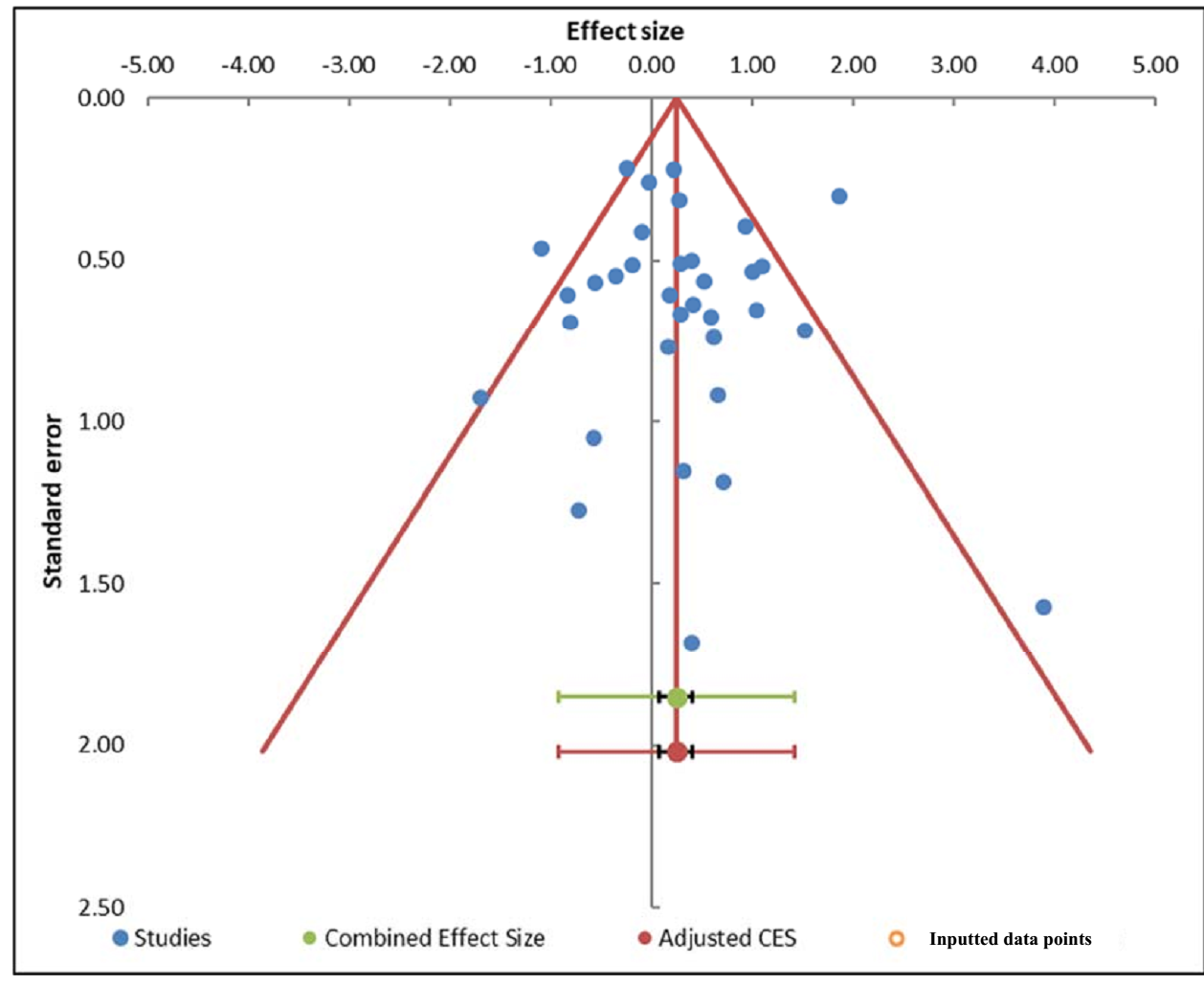


**Supplemental Figure S16.** Funnel plots for analysis of the effect of myalgia on the severity of the COVID-19 cases. To identify the possible publication bias funnel plot with combined effect size and adjusted combined effect size accompanying confidence and prediction intervals were generated. The odds ratios are plotted against the standard error for the indicated myalgia indicators.


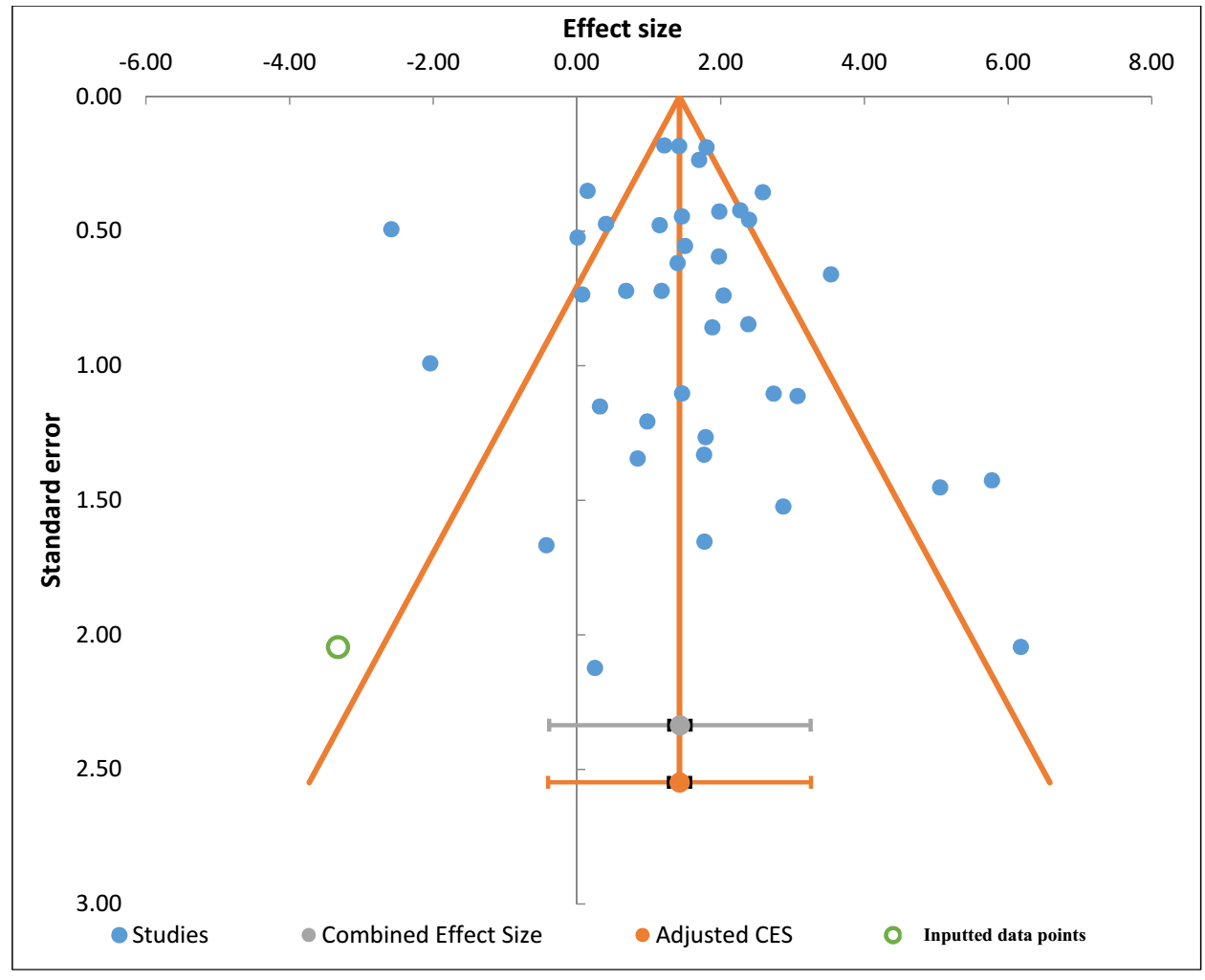


**Supplemental Figure S17.** Funnel plots for analysis of the effect of dyspnea on the severity of the COVID-19 cases. To identify the possible publication bias funnel plot with combined effect size and adjusted combined effect size accompanying confidence and prediction intervals were generated. The odds ratios are plotted against the standard error for the indicated dyspnea indicators.


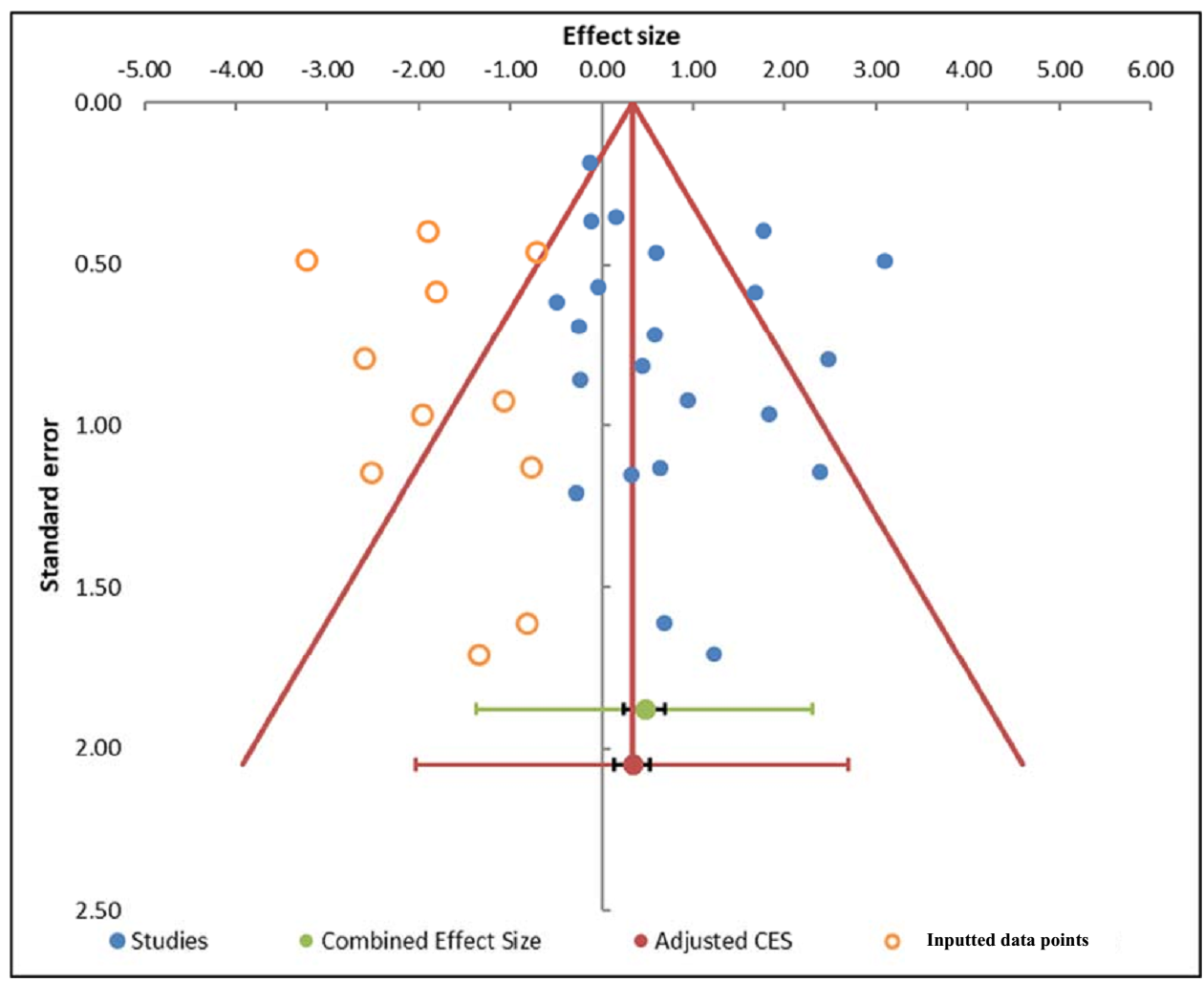


**Supplemental Figure S18.** Funnel plots for analysis of the effect of chest tightness on the severity of the COVID-19 cases. To identify the possible publication bias funnel plot with combined effect size and adjusted combined effect size accompanying confidence and prediction intervals were generated. The odds ratios are plotted against the standard error for the indicated chest tightness indicators.


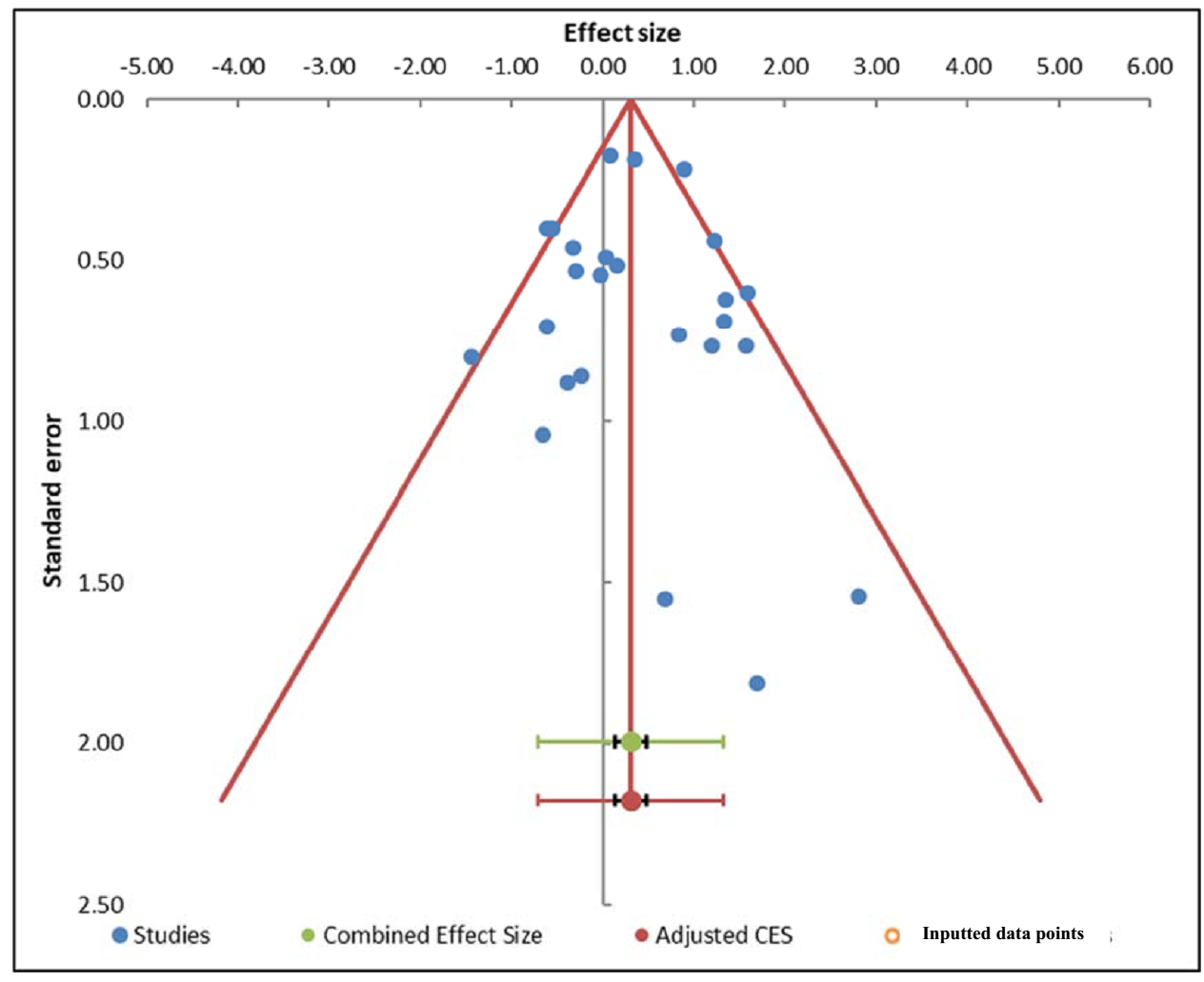


**Supplemental Figure S19**. Funnel plots for analysis of the effect of sputum production on the severity of the COVID-19 cases. To identify the possible publication bias funnel plot with combined effect size and adjusted combined effect size accompanying confidence and prediction intervals were generated. The odds ratios are plotted against the standard error for the indicated sputum production indicators.


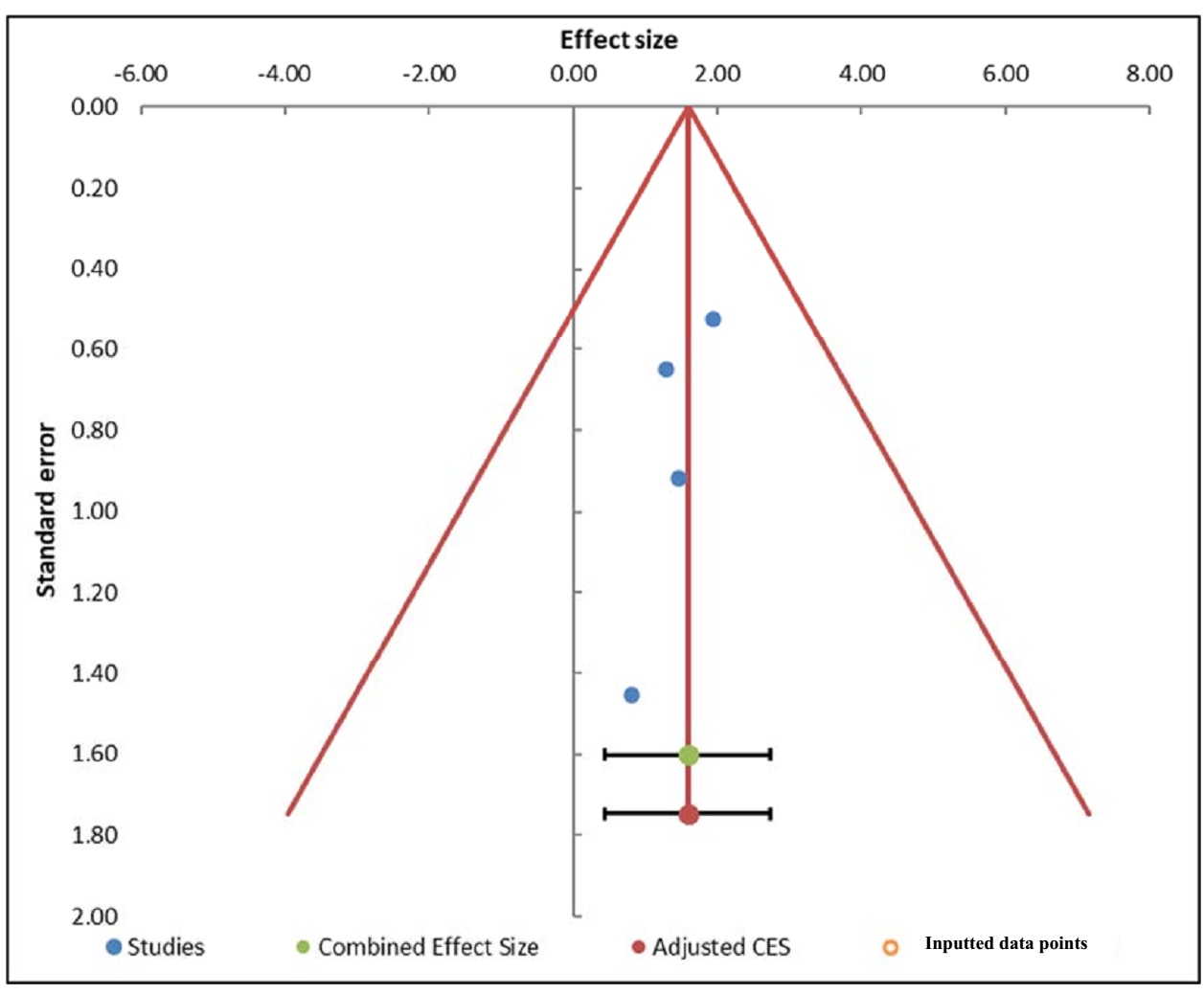


**Supplemental Figure S20.** Funnel plots for analysis of the effect of hemoptysis on the severity of the COVID-19 cases. To identify the possible publication bias funnel plot with combined effect size and adjusted combined effect size accompanying confidence and prediction intervals were generated. The odds ratios are plotted against the standard error for the indicated haemoptysis indicators.


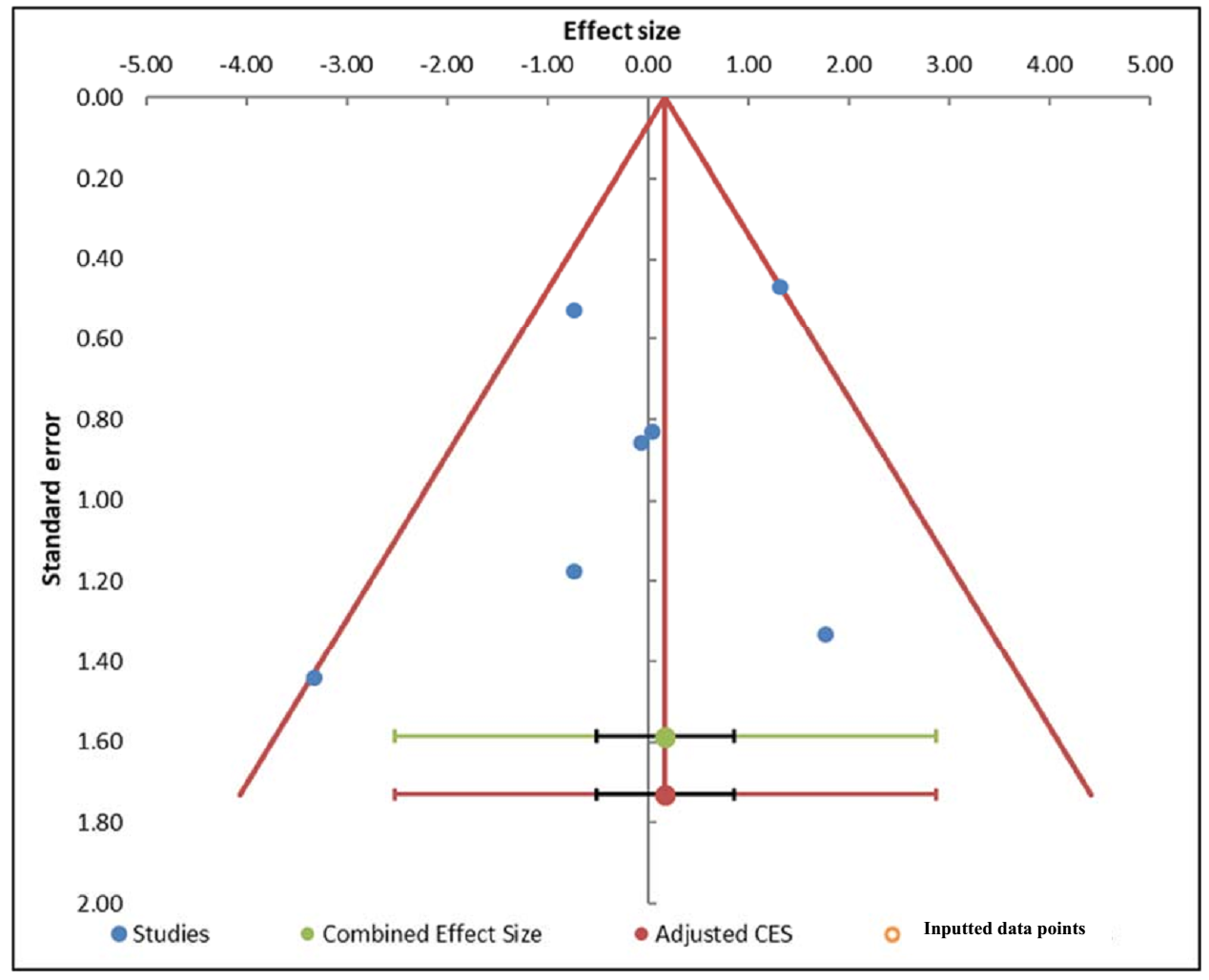


**Supplemental Figure S21.** Funnel plots for analysis of the effect of pharyngalgia on the severity of the COVID-19 cases. To identify the possible publication bias funnel plot with combined effect size and adjusted combined effect size accompanying confidence and prediction intervals were generated. The odds ratios are plotted against the standard error for the indicated pharyngalgia indicators.


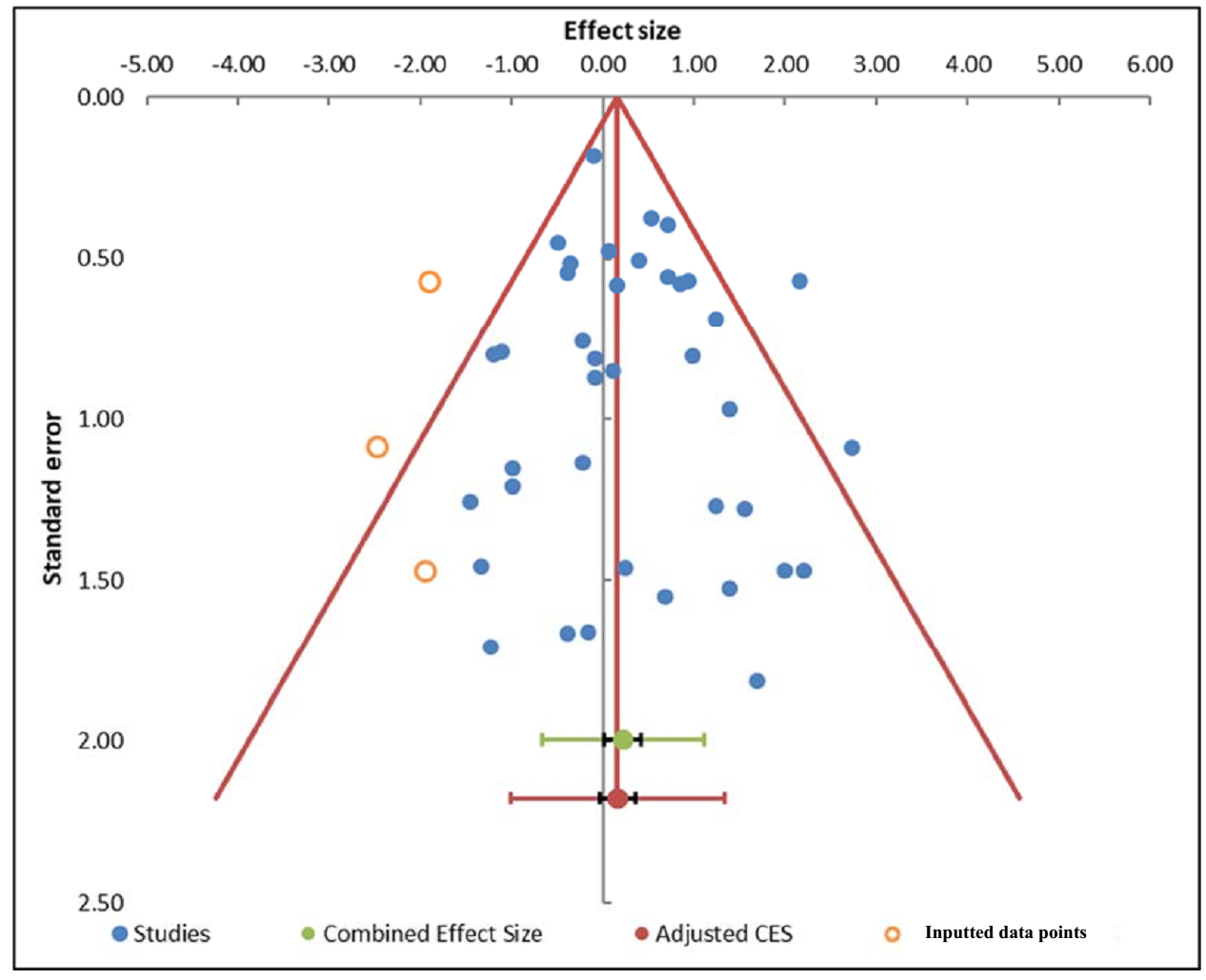


**Supplemental Figure S22.** Funnel plots for analysis of the effect of diarrhea on the severity of the COVID-19 cases. To identify the possible publication bias funnel plot with combined effect size and adjusted combined effect size accompanying confidence and prediction intervals were generated. The odds ratios are plotted against the standard error for the indicated diarrhea indicators.


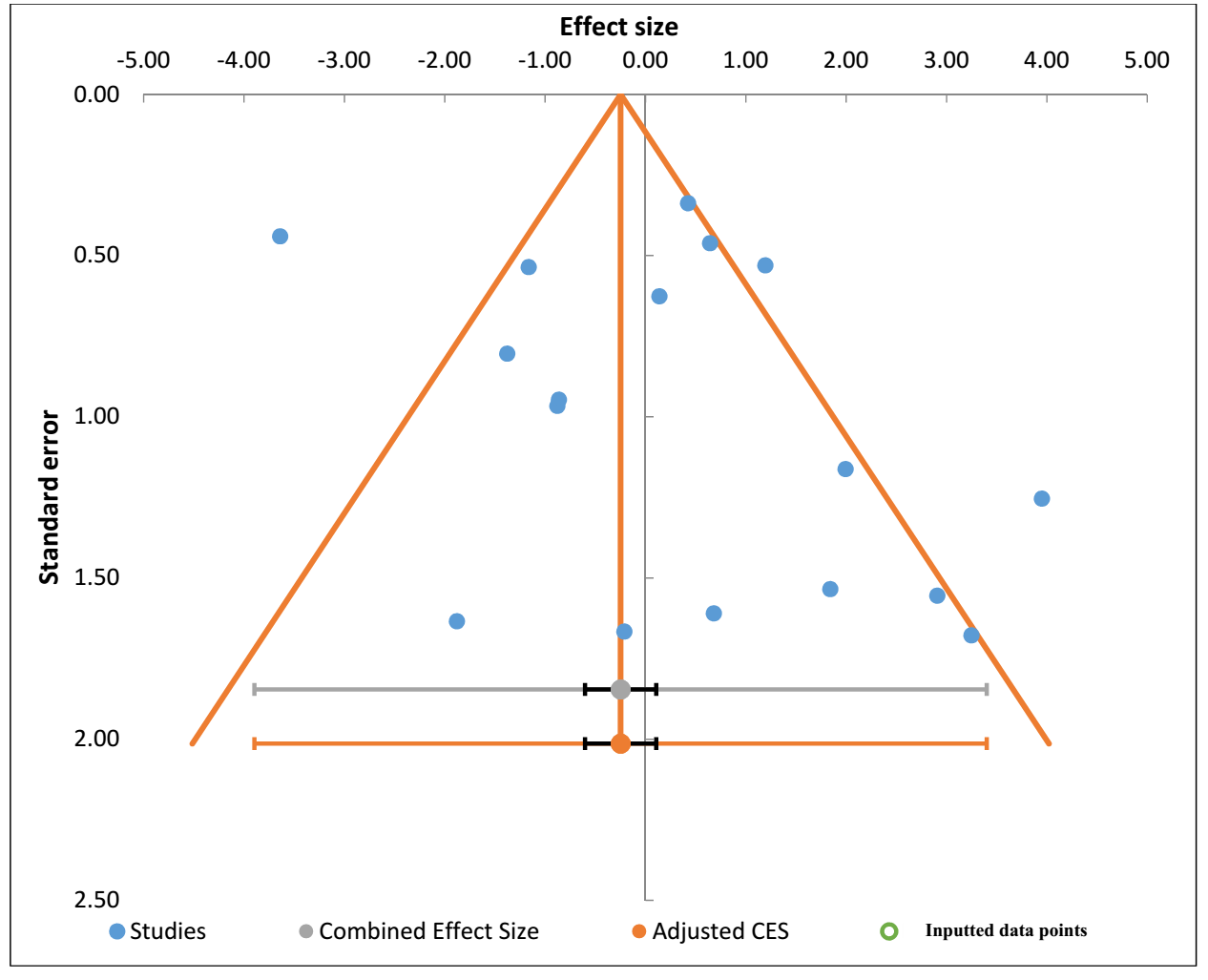


**Supplemental Figure S23.** Funnel plots for analysis of the effect of nausea on the severity of the COVID-19 cases. To identify the possible publication bias funnel plot with combined effect size and adjusted combined effect size accompanying confidence and prediction intervals were generated. The odds ratios are plotted against the standard error for the indicated nausea indicators.


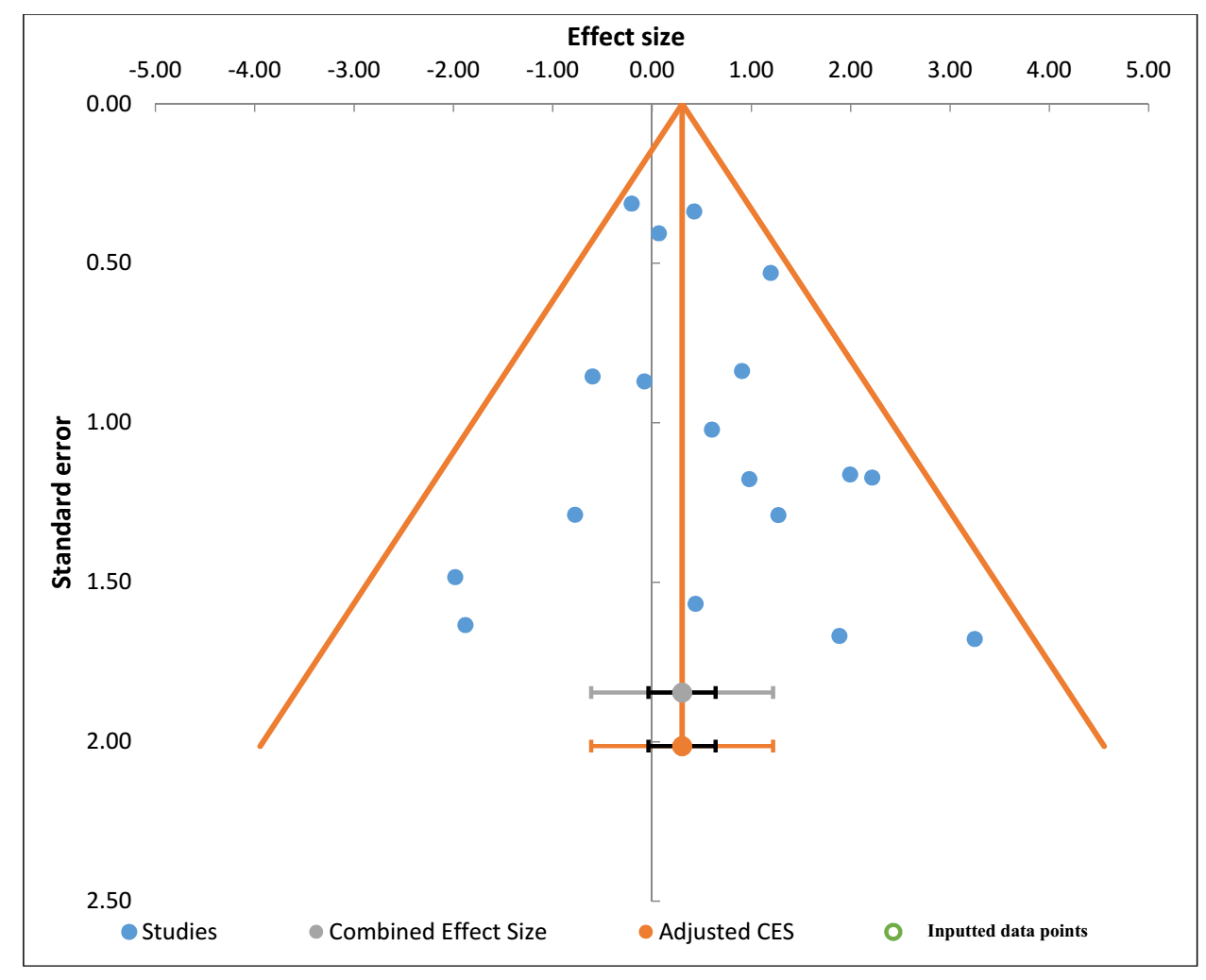


**_Inputted data points_**

**Supplemental Figure S24.** Funnel plots for analysis of the effect of vomiting on the severity of the COVID-19 cases. To identify the possible publication bias funnel plot with combined effect size and adjusted combined effect size accompanying confidence and prediction intervals were generated. The odds ratios are plotted against the standard error for the indicated vomiting indicators.


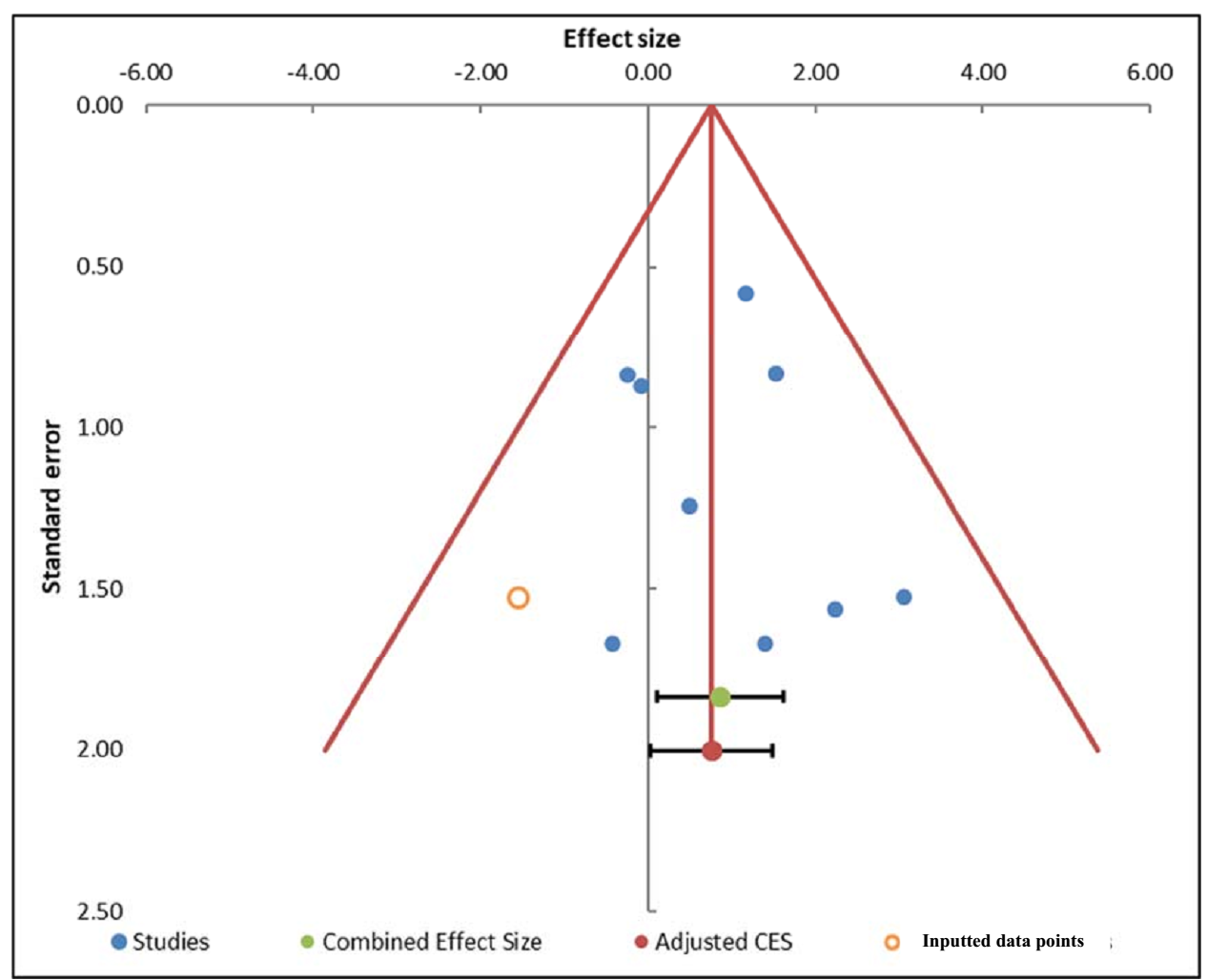


**Supplemental Figure S25**. Funnel plots for analysis of the effect of abdominal pain on the severity of the COVID-19 cases. To identify the possible publication bias funnel plot with combined effect size and adjusted combined effect size accompanying confidence and prediction intervals were generated. The odds ratios are plotted against the standard error for the indicated abdominal pain indicators.


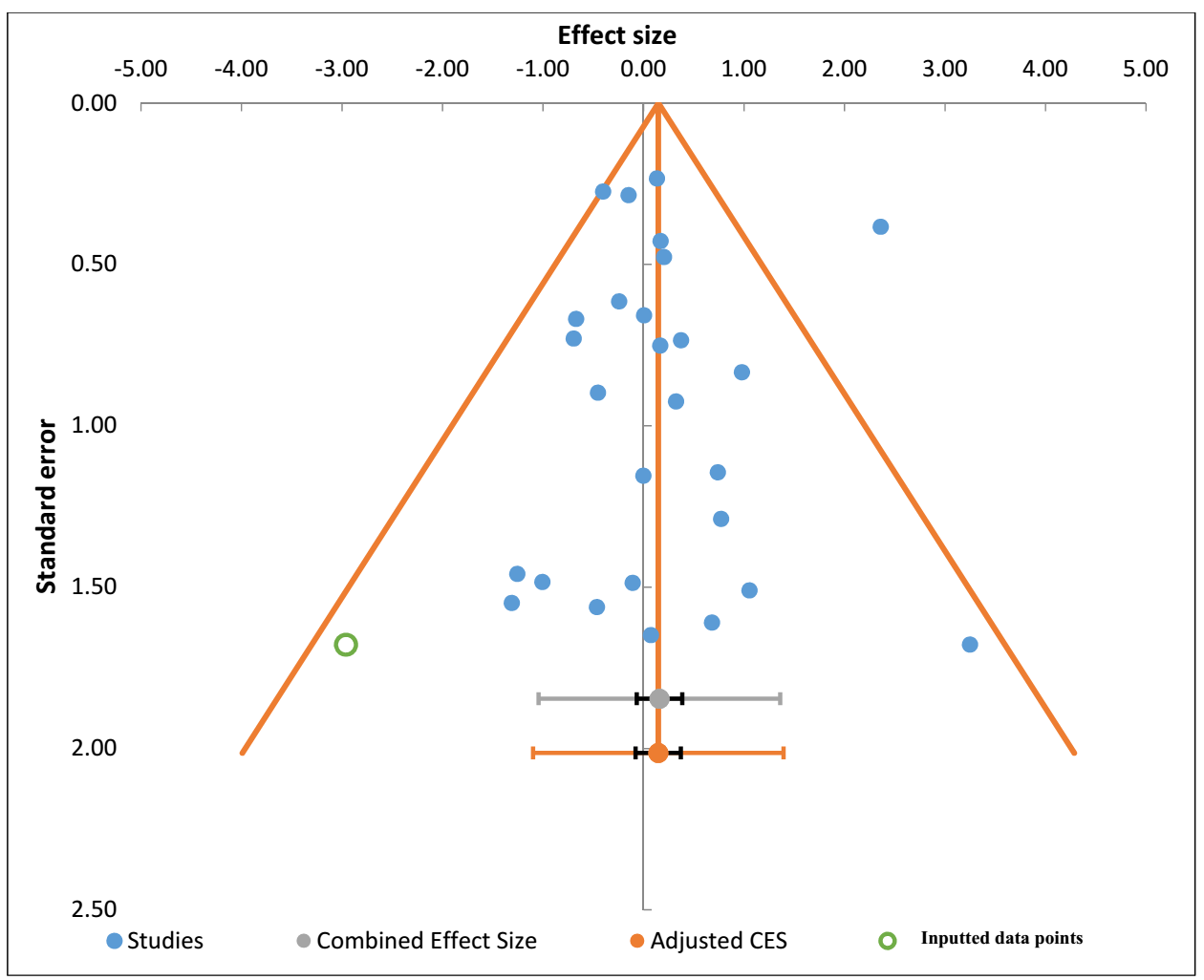


**Supplemental Figure S26.** Funnel plots for analysis of the effect of headache on the severity of the COVID-19 cases. To identify the possible publication bias funnel plot with combined effect size and adjusted combined effect size accompanying confidence and prediction intervals were generated. The odds ratios are plotted against the standard error for the indicated headache indicators.


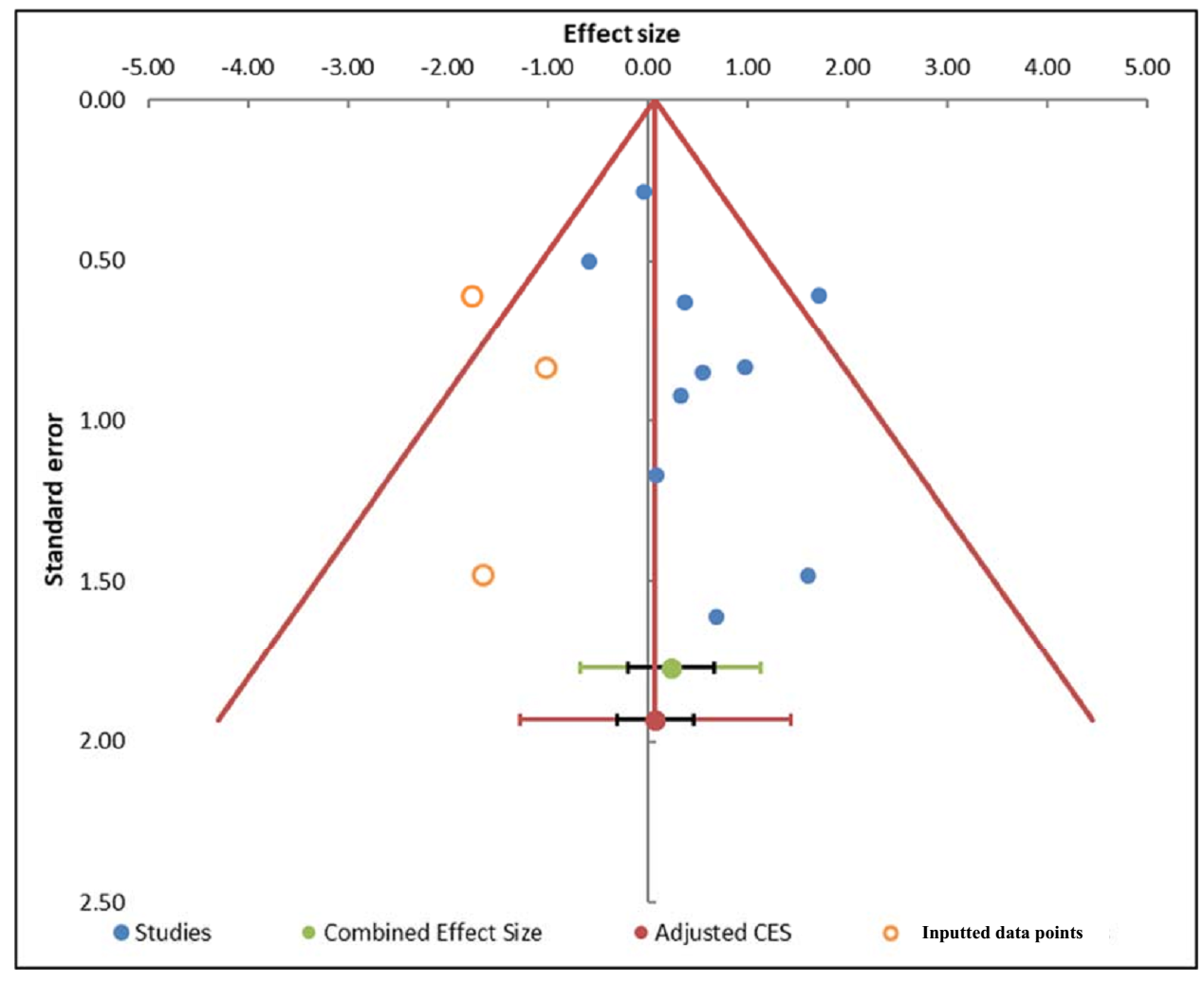


**Supplemental Figure S27.** Funnel plots for analysis of the effect of dizziness on the severity of the COVID-19 cases. To identify the possible publication bias funnel plot with combined effect size and adjusted combined effect size accompanying confidence and prediction intervals were generated. The odds ratios are plotted against the standard error for the indicated dizziness indicators.

**_Inputted data points_**


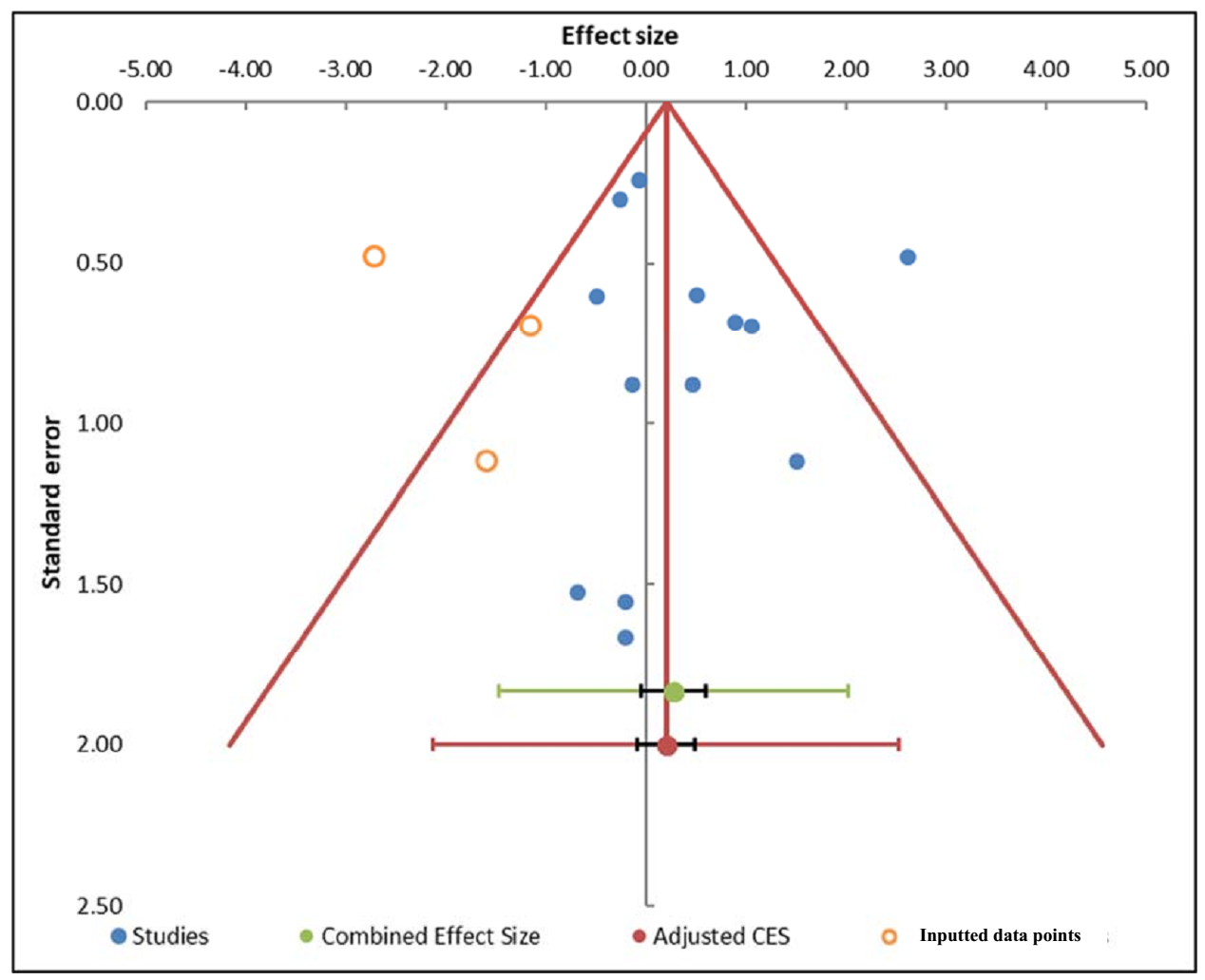


**Supplemental Figure S28.** Funnel plots for analysis of the effect of sore throat on the severity of the COVID-19 cases. To identify the possible publication bias funnel plot with combined effect size and adjusted combined effect size accompanying confidence and prediction intervals were generated. The odds ratios are plotted against the standard error for the indicated sore throat indicators.

**Supplemental Figure S29.** Sensitivity analysis for the association between sex and COVID-19 severity. In each panel, each specified study was omitted from the pooled analysis, and the effect on the total results was evaluated. Each circle and corresponding horizontal lines represent the effect size and 95% CI after the corresponding study was omitted.

**Supplemental Figure S30.** Sensitivity analysis for the association between age and COVID-19 severity. In each panel, each specified study was omitted from the pooled analysis, and the effect on the total results was evaluated. Each circle and corresponding horizontal lines represent the effect size and 95% CI after the corresponding study was omitted.

**Supplemental Figure S31.** Sensitivity analysis for the association between any comorbidity and COVID-19 severity. In each panel, each specified study was omitted from the pooled analysis, and the effect on the total results was evaluated. Each circle and corresponding horizontal lines represent the effect size and 95% CI after the corresponding study was omitted.

**Supplemental Figure S32.** Sensitivity analysis for the association between hypetension and COVID-19 severity. In each panel, each specified study was omitted from the pooled analysis, and the effect on the total results was evaluated. Each circle and corresponding horizontal lines represent the effect size and 95% CI after the corresponding study was omitted.

**Supplemental Figure S33.** Sensitivity analysis for the association between diabetes and COVID-19 severity. In each panel, each specified study was omitted from the pooled analysis, and the effect on the total results was evaluated. Each circle and corresponding horizontal lines represent the effect size and 95% CI after the corresponding study was omitted.

**Supplemental Figure S34.** Sensitivity analysis for the association between cerebrovascular disease and COVID-19 severity. In each panel, each specified study was omitted from the pooled analysis, and the effect on the total results was evaluated. Each circle and corresponding horizontal lines represent the effect size and 95% CI after the corresponding study was omitted.

**Supplemental Figure S35.** Sensitivity analysis for the association between cardiovascular disease and COVID-19 severity. In each panel, each specified study was omitted from the pooled analysis, and the effect on the total results was evaluated. Each circle and corresponding horizontal lines represent the effect size and 95% CI after the corresponding study was omitted.

**Supplemental Figure S36.** Sensitivity analysis for the association between Respiratory diseases and COVID-19 severity. In each panel, each specified study was omitted from the pooled analysis, and the effect on the total results was evaluated. Each circle and corresponding horizontal lines represent the effect size and 95% CI after the corresponding study was omitted.

**Supplemental Figure S37.** Sensitivity analysis for the association between malignancy and COVID-19 severity. In each panel, each specified study was omitted from the pooled analysis, and the effect on the total results was evaluated. Each circle and corresponding horizontal lines represent the effect size and 95% CI after the corresponding study was omitted.

**Supplemental Figure S38.** Sensitivity analysis for the association between chronic kidney disease and COVID-19 severity. In each panel, each specified study was omitted from the pooled analysis, and the effect on the total results was evaluated. Each circle and corresponding horizontal lines represent the effect size and 95% CI after the corresponding study was omitted.

**Supplemental Figure S39.** Sensitivity analysis for the association between chronic liver disease and COVID-19 severity. In each panel, each specified study was omitted from the pooled analysis, and the effect on the total results was evaluated. Each circle and corresponding horizontal lines represent the effect size and 95% CI after the corresponding study was omitted.

**Supplemental Figure S40.** Sensitivity analysis for the association between fever and COVID-19 severity. In each panel, each specified study was omitted from the pooled analysis, and the effect on the total results was evaluated. Each circle and corresponding horizontal lines represent the effect size and 95% CI after the corresponding study was omitted.

**Supplemental Figure S41.** Sensitivity analysis for the association between cough and COVID-19 severity. In each panel, each specified study was omitted from the pooled analysis, and the effect on the total results was evaluated. Each circle and corresponding horizontal lines represent the effect size and 95% CI after the corresponding study was omitted.

**Supplemental Figure S42.** Sensitivity analysis for the association between fatigue and COVID-19 severity. In each panel, each specified study was omitted from the pooled analysis, and the effect on the total results was evaluated. Each circle and corresponding horizontal lines represent the effect size and 95% CI after the corresponding study was omitted.

**Supplemental Figure S43.** Sensitivity analysis for the association between anorexia and COVID-19 severity. In each panel, each specified study was omitted from the pooled analysis, and the effect on the total results was evaluated. Each circle and corresponding horizontal lines represent the effect size and 95% CI after the corresponding study was omitted.

**Supplemental Figure S44.** Sensitivity analysis for the association between myalgia and COVID-19 severity. In each panel, each specified study was omitted from the pooled analysis, and the effect on the total results was evaluated. Each circle and corresponding horizontal lines represent the effect size and 95% CI after the corresponding study was omitted.

**Supplemental Figure S45.** Sensitivity analysis for the association between dyspnoea and COVID-19 severity. In each panel, each specified study was omitted from the pooled analysis, and the effect on the total results was evaluated. Each circle and corresponding horizontal lines represent the effect size and 95% CI after the corresponding study was omitted.

**Supplemental Figure S46.** Sensitivity analysis for the association between chest tightness and COVID-19 severity. In each panel, each specified study was omitted from the pooled analysis, and the effect on the total results was evaluated. Each circle and corresponding horizontal lines represent the effect size and 95% CI after the corresponding study was omitted.

**Supplemental Figure S47.** Sensitivity analysis for the association between sputum production and COVID-19 severity. In each panel, each specified study was omitted from the pooled analysis, and the effect on the total results was evaluated. Each circle and corresponding horizontal lines represent the effect size and 95% CI after the corresponding study was omitted.

**Supplemental Figure S48.** Sensitivity analysis for the association between haemoptysis and COVID-19 severity. In each panel, each specified study was omitted from the pooled analysis, and the effect on the total results was evaluated. Each circle and corresponding horizontal lines represent the effect size and 95% CI after the corresponding study was omitted.

**Supplemental Figure S49.** Sensitivity analysis for the association between pharyngalgia and COVID-19 severity. In each panel, each specified study was omitted from the pooled analysis, and the effect on the total results was evaluated. Each circle and corresponding horizontal lines represent the effect size and 95% CI after the corresponding study was omitted.

**Supplemental Figure S50.** Sensitivity analysis for the association between diarrhea and COVID-19 severity. In each panel, each specified study was omitted from the pooled analysis, and the effect on the total results was evaluated. Each circle and corresponding horizontal lines represent the effect size and 95% CI after the corresponding study was omitted.

**Supplemental Figure S51.** Sensitivity analysis for the association between nausea and COVID-19 severity. In each panel, each specified study was omitted from the pooled analysis, and the effect on the total results was evaluated. Each circle and corresponding horizontal lines represent the effect size and 95% CI after the corresponding study was omitted.

**Supplemental Figure S52.** Sensitivity analysis for the association between vomiting and COVID-19 severity. In each panel, each specified study was omitted from the pooled analysis, and the effect on the total results was evaluated. Each circle and corresponding horizontal lines represent the effect size and 95% CI after the corresponding study was omitted.

**Supplemental Figure S53.** Sensitivity analysis for the association between abdominal pain and COVID-19 severity. In each panel, each specified study was omitted from the pooled analysis, and the effect on the total results was evaluated. Each circle and corresponding horizontal lines represent the effect size and 95% CI after the corresponding study was omitted.

**Supplemental Figure S54.** Sensitivity analysis for the association between headache and COVID-19 severity. In each panel, each specified study was omitted from the pooled analysis, and the effect on the total results was evaluated. Each circle and corresponding horizontal lines represent the effect size and 95% CI after the corresponding study was omitted.

**Supplemental Figure S55.** Sensitivity analysis for the association between dizziness and COVID-19 severity. In each panel, each specified study was omitted from the pooled analysis, and the effect on the total results was evaluated. Each circle and corresponding horizontal lines represent the effect size and 95% CI after the corresponding study was omitted.

**Supplemental Figure S56.** Sensitivity analysis for the association between the sore throat and COVID-19 severity. In each panel, each specified study was omitted from the pooled analysis, and the effect on the total results was evaluated. Each circle and corresponding horizontal lines represent the effect size and 95% CI after the corresponding study was omitted.

**Supplementary Table S1.** Quality assessment of the included observational studies by NOS

| **Author** | **Year** | **Selection** | | | |  | **Comparability** | |  | **Outcome** | | | **Total Score** |
| --- | --- | --- | --- | --- | --- | --- | --- | --- | --- | --- | --- | --- | --- |
|  |  | 1 | 2 | 3 | 4 |  | 5A | 5B |  | 6 | 7 | 8 |  |
|  |  | Exposed cohort truly/somewhat representative | Nonexposed cohort drawn from the same community | Ascertainment of exposure | Outcome of interest not present at start |  | Cohorts adjusted for age | Cohorts adjusted for other important factor(s) |  | Quality of outcome assessment | Follow-up/  Observation period  Long enough for outcomes to occur | Adequacy of follow-up of cohorts |  |
| Aggarwal S | 2020 | * | * | * | * |  | * |  |  | * | * | - | 7 |
| Bi X | 2020 | * |  | * | * |  | * |  |  | * | * |  | 6 |
| Cai Q | 2020 | * | - | * | * |  |  | * |  | * | * | * | 7 |
| Cai Y | 2020 | * | - | * | * |  | * | * |  | * | * | * | 8 |
| Chen G | 2020 | * | * |  | * |  |  |  |  | * | * | * | 7 |
| Chen X | 2020 | * | * |  | * |  | * |  |  | * | * |  | 6 |
| Chen Q | 2020 | * | * | * | * |  |  |  |  | * | * |  | 6 |
| Chu J | 2020 | * | * | * | * |  |  |  |  | * | * | * | 7 |
| Colaneri M | 2020 | * |  | * | * |  |  | * |  | * | * | * | 7 |
| Deng Q | 2020 | * |  | * | * |  |  |  |  | * | * | * | 6 |
| Feng Y | 2020 | * | * | * | * |  |  | * |  | * | * | * | 8 |
| Ferguson J | 2020 | * | * | * | * |  |  | * |  | * | * |  | 7 |
| Gao Y | 2020 | * |  | * | * |  | * |  |  | * | * |  | 6 |
| Guan WJ | 2020 | * | * | * | * |  |  |  |  | * | * |  | 6 |
| He R | 2020 | * | * | * | * |  |  |  |  | * | * |  | 6 |
| Hong KS | 2020 | * |  | * | * |  |  |  |  | * | * | * | 6 |
| Huang C | 2020 | * | * | * | * |  |  |  |  | * | * |  | 6 |
| Huang Q | 2020 | * | * | * | * |  |  |  |  | * | * |  | 6 |
| Huang R | 2020 | * | * | * | * |  |  |  |  | * | * | * | 7 |
| Jiang Y | 2020 | * | * | * | * |  |  |  |  | * | * |  | 6 |
| Ketcham SW | 2020 | * | * | * | * |  |  | * |  | * | * |  | 7 |
| Lei S | 2020 | * |  | * | * |  |  | * |  | * | * |  | 6 |
| Li K | 2020 | * |  | * | * |  |  |  |  | * | * | * | 6 |
| Li S | 2020 | * |  | * | * |  |  | * |  | * | * |  | 6 |
| Li X | 2020 | * | * | * | * |  |  |  |  | * | * |  | 6 |
| Li YK | 2020 | * |  | * | * |  |  |  |  | * | * |  | 6 |
| Liang W | 2020 | * |  | * | * |  | * |  |  | * | * |  | 6 |
| Liu F | 2020 | * |  | * | * |  | * |  |  | * | * |  | 6 |
| Liu J | 2020 | * | * | * | * |  |  | * |  | * | * |  | 7 |
| Liu Z | 2020 | * | * | * | * |  |  |  |  | * | * |  | 6 |
| Lodigiani C | 2020 | * |  | * | * |  | * |  |  | * | * |  | 6 |
| Lv Z | 2020 | * |  | * | * |  | * |  |  | * | * |  | 6 |
| Lyu P | 2020 | * |  | * | * |  | * | * |  | * | * |  | 7 |
| Pan L | 2020 | * |  | * | * |  |  |  |  | * | * |  | 6 |
| Peng YD | 2020 | * |  | * | * |  |  | * |  | * | * |  | 6 |
| Pereira MR | 2020 | * |  | * | * |  | * |  |  | * | * |  | 6 |
| Shi Y | 2020 | * |  | * | * |  | * |  |  | * | * |  | 6 |
| Sun L | 2020 | * |  | * | * |  |  | * |  | * | * |  | 6 |
| Tian S | 2020 | * |  | * | * |  |  |  |  | * | * | * | 6 |
| Wan S | 2020 | * | * | * | * |  |  |  |  | * | * |  | 6 |
| Wang D | 2020 | * |  | * | * |  |  | * |  | * | * | * | 7 |
| Wang R | 2020 | * |  | * | * |  | * |  |  | * | * | * | 7 |
| Wang F | 2020 | * |  | * | * |  |  |  |  | * | * | * | 6 |
| Wu J | 2020 | * | * | * | * |  |  |  |  | * | * |  | 6 |
| Xie H | 2020 | * |  | * | * |  |  |  |  | * | * | * | 6 |
| Xie J | 2020 | * |  | * | * |  |  |  |  | * | * |  | 6 |
| Xiong F | 2020 | * |  | * | * |  | * |  |  | * | * |  | 6 |
| Yang AP | 2020 | * | * | * | * |  | * |  |  | * | * |  | 7 |
| Yang P | 2020 | * |  | * | * |  |  | * |  | * | * |  | 6 |
| Yang Y | 2020 | * | * | * | * |  |  |  |  | * | * |  | 6 |
| Yao Q | 2020 | * | * | * | * |  |  |  |  | * | * |  | 6 |
| Yu X | 2020 | * |  | * | * |  | * |  |  | * | * |  | 6 |
| Zhang JJ | 2020 | * | * | * | * |  |  |  |  | * | * |  | 6 |
| Zheng S | 2020 | * |  | * | * |  | * |  |  | * | * |  | 6 |
| Zhou Y | 2020 | * | * | * | * |  |  | * |  | * | * |  | 7 |

NOS=Newcastle Otawa Scale; in this scale score ranges between 0-9 where “0-3” indicates low quality, “4-5” indicates moderate quality and score of ≥6 indicates high quality study
